# Identification of a uniquely expanded V1R (ORA) gene family in the Japanese grenadier anchovy (*Coilia nasus*)

**DOI:** 10.1007/s00227-016-2896-9

**Published:** 2016-05-02

**Authors:** Guoli Zhu, Wenqiao Tang, Liangjiang Wang, Cong Wang, Xiaomei Wang

**Affiliations:** College of Fisheries and Life Science, Shanghai Ocean University, Shanghai, China; Department of Genetics and Biochemistry, Clemson University, Clemson, South Carolina USA

## Abstract

A number of studies have suggested that olfaction plays an important role in fish migration. Fish use several distinct families of olfactory receptors to detect environmental odorants, including MORs (main olfactory receptors), V1Rs (vomeronasal type-1 receptors), V2Rs (vomeronasal type-2 receptors), TAARs (trace amine-associated receptors), and FPRs (formyl peptide receptors). The V1Rs have been reported to detect pheromones, and a pheromone hypothesis for the spawning migration of anadromous fish has been proposed. Examining whether *Coilia nasus* relies on V1R-mediated olfaction for spawning migration is important for understanding the molecular basis of spawning migration behavior. Here, we explored the V1R gene family in anadromous *C. nasus*. Six V1R genes previously reported in other teleost fish were successfully identified. Interestingly, we detected the largest V1R repertoire in teleost fish from *C. nasus* and identified a species-specific expansion event of V1R3 gene that has previously been detected as single-copy genes in other teleost fish. The V1R loci were found to be populated with repetitive sequences, especially in the expanded V1R3 genes. Additionally, the divergence of V1R3 genetic structures in different populations of *C. nasus* indicates the copy number variation (CNV) in V1R3 gene among individuals of *C. nasus*. Most of the putative *C. nasus* V1R genes were expressed primarily in the olfactory epithelium, consistent with the role of the gene products as functional olfactory receptors. Significant differences in the expression levels of V1R genes were detected between the anadromous and non-anadromous *C. nasus*. This study represents a first step in the elucidation of the olfactory communication system of *C. nasus* at the molecular level. Our results indicate that some V1R genes may be involved in the spawning migration of *C. nasus*, and the study provides new insights into the spawning migration and genome evolution of *C. nasus*.

**Electronic supplementary material:**

The online version of this article (doi:10.1007/s00227-016-2896-9) contains supplementary material, which is available to authorized users.

## Introduction

The Japanese grenadier anchovy *Coilia nasus* is a famous small-sized commercial fish that is widely distributed in the coastal waters of China, the middle and lower reaches of the Yangtze River, Ariake Bay of Japan, and the western coastal waters of the Korean Peninsula (Whitehead et al. 1998; Zhu et al. 2014; Yuan et al. 1980). Anadromous *C. nasus* lives in the sea until it reaches sexual maturity and then performs ocean–river migration behavior to the exorheic rivers through long-distance swimming to spawn (Whitehead et al. 1998; Zhu et al. 2014; Yuan et al. 1980). However, the molecular mechanisms of spawning migration of anadromous *C. nasus* have not been clarified yet.

Olfactory hypotheses in fish migration propose that the spawning migration of fish is mediated by olfactory cues. Indeed, migratory fish lose the ability to perform accurate migrations following alterations to their olfactory epithelia (McBride et al. 1964; Tarrant 1966; Jahn 1967; Doving et al. 1985; Yano and Nakamura 1992; Barbin et al. 1998). Strong olfactory responses to natal stream water have also been found in migratory sockeye salmon through blood oxygenation level-dependent functional magnetic resonance imaging (Bandoh et al. 2011). Some olfactory receptor genes have been demonstrated to be differentially expressed during the different life stages of the wild anadromous Atlantic salmon, but not in the non-anadromous population (Johnstone et al. 2011). Thus, a functional olfactory ability is essential for migration to their natal streams. Therefore, we hypothesized that olfaction also played an important role in the spawning migration of *C. nasus*.

Pheromones play essential roles in many intraspecies communications, such as mating preferences, individual recognition and aggression. Based on previous research, two different olfactory hypotheses have been proposed for salmon imprinting and homing: One imprinting hypothesis was developed in Coho salmon (*Oncorhynchus kisutch*) (Wisby and Hasler 1954), while the second is the pheromone hypothesis developed in Arctic char (*Salvelinus alpinus*) and Atlantic salmon (*Salmo salar*) (Nordeng 1971, 1977). The pheromone hypothesis states that juvenile salmon release population-specific odors in a stream to guide the homing adults in the return to their natal waters during their migration. Furthermore, a mixture of sulfated steroids has been shown to function as a migratory pheromone; these steroids are released by stream-dwelling larval lamprey to guide adults to spawning streams (Sorensen et al. 2005). Therefore, if olfaction plays an important role in the spawning migration of *C. nasus*, some pheromones may be used as the olfactory properties detected by *C. nasus* migrants to guide them during anadromous migration.

Vertebrate species distinguish a large number of chemical cues in the environment through the olfactory receptors (ORs) (Laberge and Hara 2001; Hino et al. 2009; Fuss and Ray 2009; Zhu et al. 2015). The ORs are G protein-coupled receptors with seven transmembrane domains that are encoded by corresponding chemosensory receptor genes (Kaupp 2010; Zhang and Firestein 2009; Korsching 2009). In mammals, two organs function as the main olfactory organ and the vomeronasal organ to perform olfactory functions. Five types of olfactory receptors derived from evolutionarily distinct multigene families to detect chemical cues in the outer environment have been detected in vertebrates, including the main olfactory receptors (MORs) (Buck and Axel 1991), vomeronasal type-1 receptors (V1Rs), vomeronasal type-2 receptors (V2Rs) (Ryba and Tirindelli 1997), trace amine-associated receptors (TAARs) (Liberles and Buck 2006), and formyl peptide receptors (Riviere et al. 2009). Because fish do not possess a vomeronasal system, their corresponding ORs are expressed in the olfactory epithelium of the nasal cavity (Pfister and Rodriguez 2005; Cao et al. 1998; Asano-Miyoshi et al. 2000). V1Rs and V2Rs in fish were named following mammalian nomenclature when they were first identified. Recently, the orthologous V1R genes in fish have been named ORAs (Saraiva and Korsching 2007; Johnstone et al. 2008), while the V2R orthologs have been named OlfCs (Alioto and Ngai 2006).

V1R (ORA) is a fish olfactory receptor gene family that was recently identified (Saraiva and Korsching 2007). The fish V1R gene family is small, with only six members; all of them are highly conserved in the analyzed teleost fish species, with the exception of *Takifugu rubripes* and *Tetraodon nigroviridis* in which V1R2 is absent (Pfister and Rodriguez 2005; Saraiva and Korsching 2007; Shi and Zhang 2007; Pfister et al. 2007). Studies have demonstrated the sequence conservation of orthologous V1R genes among salmonid species and between distantly related rockfish species (Johansson and Banks 2011; Johnson and Banks 2011). Therefore, the V1R gene family is unique compared with the MOR, V2R, and TAAR gene families, which have experienced extensive lineage-specific expansions (Saraiva and Korsching 2007; Niimura and Nei 2005; Hashiguchi et al. 2008; Hussain et al. 2009; Nei et al. 2008). The existence of the orthologous fish V1R1 gene in elephant sharks (*Callorhinchus milii*) and an orthologous gene of the fish V1R2 in frogs (*Xenopus tropicalis*) indicates that the V1R genes have been maintained throughout the evolutionary history of aquatic vertebrates (Grus and Zhang 2009). The strong sequence conservation of V1Rs suggests their functional significance and implies that they can distinguish evolutionarily conserved chemicals, such as reproductive pheromones (Saraiva and Korsching 2007; Johnson and Banks 2011). Indeed, V1Rs in mammals can detect low molecular weight molecules such as steroids (Boschat et al. 2002; Del Punta et al. 2002; Isogai et al. 2011). V1R1 (ORA1), which is a zebrafish olfactory receptor that is ancestral to all mammalian V1R genes (Saraiva and Korsching 2007), has been proven to recognize 4-hydroxyphenylacetic acid, which is a putative reproductive pheromone used to elicit increases in oviposition frequency in zebrafish mating pairs, with high specificity and sensitivity (Behrens et al. 2014). Therefore, the V1Rs are the putative pheromone receptors.

In order to understand the feasible relationship between olfaction and spawning migration behavior, it is worthwhile to investigate the putative pheromone V1R receptors in *C. nasus*. Previously, we have reported the de novo transcriptomes of the olfactory epithelium in *C. nasus* (Zhu et al. 2014) and that work will be helpful for this study.

In this study, we described the V1R gene family of *C. nasus* through data mining and phylogenetic analysis. Six intact V1R gene members that are orthologous to the known V1R genes were identified. Additionally, we identified a species-specific expansion event of V1R3 gene subfamily, which have previously been reported to exist as a single copy in other teleost fish. We used real-time relative quantitative PCR to detect the expression profiles in the tissues of all the members of the V1R gene family in *C. nasus*. Furthermore, we investigated this gene family’s potential role in the migratory behavior of the anadromous and non-anadromous forms of *C. nasus* by analyzing the expression levels of all the V1R genes in olfactory rosettes in both forms. Based on our results, we propose that some V1R genes play an important role in the spawning migration behavior of *C. nasus*.

## Materials and methods

### Ethics statement

This work was approved by the Institutional Animal Care and Use Committee of Shanghai Ocean University and was performed following the Guidelines on the Care and Use of Animals for Scientific Purposes set by the Institutional Animal Care and Use Committee of Shanghai Ocean University.

### Fish and tissue samples

The anadromous *C. nasus* migrants used in this study were captured in early May 2014 from the Jingjiang section of the Yangtze River in Jingjiang, Jiangsu Province, on the east coast of China when the fish were migrating upstream along the river. The fish were captured with the help of fisherman with fishing license No. SuChuanBu (2011) JMF217 and a special fishing license for *C. nasus* in the Yangtze River (No. SuChuanBu 2014 ZX-M025) permitted by the Jiangsu Provincial Oceanic and Fishery Bureau. Non-anadromous *C. nasus* individuals from Poyanghu Lake in Jiujiang, Jiangxi Province, in the southeast of China were captured in late March 2014, before the anadromous *C. nasus* migrated to the lake. These fish were also captured with the assistance of fisherman owning a fishing license (No. 0400051) permitted by the Jiangxi Provincial Department of Agriculture.

Immediately after capture, live fish from the wild waters were buried in medical ice bags with a temperature of −20 °C until loss of consciousness was achieved. The average time to loss of consciousness for the fish was estimated to be 3 min. Then, the captured fish were rapidly dissected on ice and examined for anatomical characteristics of the gonadal development phase (Xu et al. 2011, 2012). The olfactory rosettes of *C. nasus* in phase III and other tissues used in this work were collected. To prevent RNA degradation, all of the operations on *C. nasus* were finished within 10 min of loss of consciousness. All efforts were made to minimize suffering. The sampled tissues for RNA experiments were stored in RNALater (Ambion, USA), kept at 4 °C overnight and transported to Shanghai Ocean University at −20 °C.

Muscle tissue for DNA experiments from the fresh-caught fish was fixed in 100 % ethanol and stored at 4 °C. The muscle tissue from *C. nasus* from the waters of Zhoushan and Taihu Lakes was transported to the fish specimen room in Shanghai Ocean University, and the muscle tissue from the *C. nasus* of Dongtinghu Lake was provided by Dr. Dong Liu from Shanghai Ocean University. Genomic DNA from the muscle tissue of one *C. nasus* was extracted using the Genomic DNA Purification Kit (Lifefeng, China) and subsequently stored at −20 °C in water.

### PCR and DNA sequencing

**Fig. 1:**
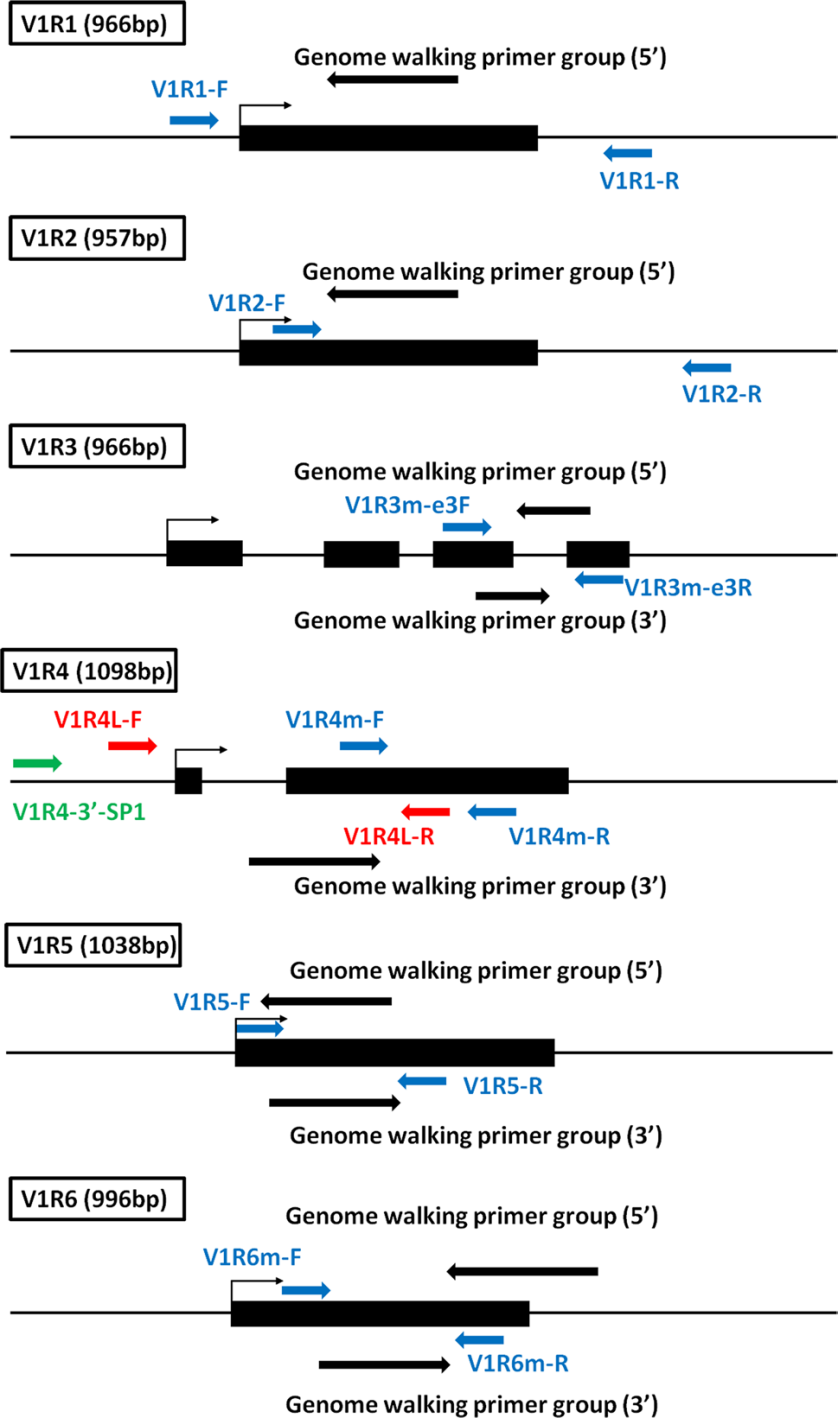
Schematic figures showing the (approximate) position of the different primers or genome walking primer groups used in the isolation of V1R genes. Predicted exons and introns of V1R genes are represented, respectively, by *solid black boxes* and *lines*. The names of V1R genes and lengths of their coding regions are shown in the *upper left*. The transcriptional direction is indicated by *small arrows*. The orientation and position of the primers used for genome walking and normal PCR are indicated by *large arrows* with different colors

The primers used to amplify the complete open reading frame of the V1R1 and V1R2 genes from the genomic DNA of anadromous *C. nasus* from the Jingjiang section of the Yangtze River were designed according to the transcripts produced by transcriptome sequencing (Zhu et al. 2014) (Fig. 1; Supplementary Table S1; Supplementary Text S2). The internal regions of the V1R3 and V1R6 genes were amplified with primers from the literature (Ota et al. 2012). PCR was performed in an Eppendorf Mastercycler (Eppendorf, Germany) with approximately 50 ng of genomic DNA, 1U of DNA Taq plus polymerase (including dNTP and buffer, Tiangen, Shanghai), and 5 pmol of each primer in a total volume of 25 µl. The PCRs were performed as follows: initial denaturation for 5 min at 95 °C, then 30 cycles of denaturation for 45 s at 95 °C, annealing for 45 s at the corresponding temperature, and extension for 1 min 30 s at 72 °C, followed by a 10-min additional extension at 72 °C and storage at 4 °C. The PCR products were subjected to electrophoresis, the target DNA bands were isolated and purified, and then the obtained DNA fragments were ligated into T-vectors and transformed into Escherichia coli (DH5α). The clones were identified by PCR with primers M13-34 and RV-M, and the positive clones were sequenced. V1R4 and V1R5 sequences (not intact) were obtained directly from the transcriptome data.

The 5′-and 3′-flanking sequences of the V1R1, V1R2, V1R3, V1R5, and V1R6 genes were obtained using the Genome Walking kit (Takara, Japan) with primers designed based on the obtained sequences. The V1R4 flanking sequence was obtained through a round of the genome walking reaction, followed by a PCR using the above product as the template and primers from the literature (Ota et al. 2012); then, genome walking and PCR were performed to gain the 3′- region of the V1R4 gene. Finally, the DNA bands produced were purified, cloned, and sequenced. The obtained sequences were edited using BIOEDIT 6.0.7 (Hall 1999) and annotated with TBLASTX in the NCBI database (http://www.ncbi.nlm.nih.gov/). The primers used are shown in Table 1.

**Table 1:** V1R (ORA) genes identified in *C. nasus* (pseudogenes are in brackets)

Species	V1R1	V1R2	V1R3	V1R4	V1R5	V1R6	Total	References
*Danio rerio*	1	1	1	1	1	1	6	Pfister and Rodriguez (2005), Saraiva and Korsching (2007) and Shi and Zhang (2007)
*Salmo salar*	1	1	2	1	2	(1)	7 (1)	Johnstone et al. (2012)
*Oryzias latipes*	1	1	1	1	1	1	6	Pfister and Rodriguez (2005), Saraiva and Korsching (2007) and Shi and Zhang (2007)
*Tetraodon nigroviridis*	1	0	1	1	1	1	5	Pfister and Rodriguez (2005), Saraiva and Korsching (2007) and Shi and Zhang (2007)
*Takifugu rubripes*	1	0	1	1	1	1	5	Pfister and Rodriguez (2005) and Saraiva and Korsching (2007)
*Gasterosteus aculeatus*	1	1	1	1	1	1	6	Saraiva and Korsching (2007) and Shi and Zhang (2007)
*Haplochromis chilotes*	1	1	1	1	1	1	6	Ota et al. (2012)
*Coilia nasus*	1	1	6 (1)	1	1	1	11	This study

### 5′ UTR sequence motif

A conserved sequence motif in the internal sequences of V1R1–V1R2 with the length of about 75 bp has been identified in the already investigated teleost fish (Pfister et al. 2007; Ota et al. 2012). The approximately 75-bp sequence of the V1R1 5′-UTR homology region of *D. rerio*, *G. aculeatus*, *O. latipes*, *T. nigroviridis*, *T. rubripes*, *S. salar*, *H. chilotes* and the corresponding sequence from *C. nasus* were aligned with the software Clustal X 2.0 for initial identification (Thompson et al. 1997). A logo was produced using the sequence logo software (http://weblogo.berkeley.edu/logo.cgi).

### Phylogenetic analysis

The sequences of V1R genes from *D. rerio*, *G. aculeatus*, *O. latipes*, *T. nigroviridis*, and *T. rubripes* were downloaded from the published literature (Saraiva and Korsching 2007). Then, TBLASTN searches were conducted on the genomes of the above fish in the ENSEMBL genome browser (http://www.ensembl.org/index.html). The V1R sequences from *S. salar* and *H. chilotes* were collected from published papers (Ota et al. 2012; Johnstone et al. 2012). The MEGA 5.05 software was used to perform the alignment of amino acid sequences, calculation of distances, and construction of neighbor-joining trees (Tamura et al. 2011).

### V1R genes in different populations of *C. nasus*

Following the amplification of the internal region of the V1R3 gene with degenerate oligos obtained from the published literature (Ota et al. 2012), we obtained a sequence with repeat sequences. Through blasting in NCBI, we found that these repeats were inserted into the second intron of the V1R3 gene. Therefore, we designed a pair of primers flanking the microsatellite to amplify the genomic DNA from the different populations of *C. nasus* (Supplementary Table S3) through PCR; the populations were collected from the Jingjiang section of the Yangtze River, the seawaters of Zhoushan, Taihu Lake, Poyanghu Lake, and Dongtinghu Lake. The population from Jingjiang represents the anadromous type of *C. nasus*, the population from Zhoushan represents the marine type of *C. nasus*, the population from Taihu Lake represents the landlocked type of *C. nasus*, and the populations from Poyanghuhu Lake and Dongtinghu Lake represent the non-anadromous type (freshwater resident) of *C. nasus*. Four fish from each population were used to represent their population in the study.

### Relative real-time quantitative PCR

To investigate the expression profiles of the V1R genes in tissues, the following tissues were extracted from three anadromous adult *C. nasus* and pooled for RNA extraction, respectively: the olfactory sensory organ (including the female olfactory rosette and male olfactory rosette), liver, heart, gill, muscle, ovary, testis, eye, and stomach. To investigate the differential expression profiles of the V1R genes between anadromous and non-anadromous *C. nasus*, the olfactory rosettes from five wild female anadromous *C. nasus* captured from the Jingjiang section of the Yangtze River and five wild female non-anadromous *C. nasus* captured from Poyanghu Lake were dissected and, respectively, pooled for further RNA extraction.

Total RNA was extracted from the above tissues with the Trizol reagent (Invitrogen, USA) following the user manual. cDNA was produced with the First-Strand cDNA synthesis Kit (Takara, Japan). To prevent cross-amplification, the PCR primers were designed based on the regions of the V1R genes that did not possess any appreciable sequence identity to one another (Supplementary Table S4). From our transcriptome data, we identified three transcripts of the V1R3 gene and named them Unigene12966_All (V1R3-1), CL5470.Contig1_All (V1R3-2), and Unigene116116_All (V1R3-3). Two of them (V1R3-1 and V1R3-2) were used as templates to design qPCR primers.

Relative expression qPCR was performed using a Bio-Rad PTC-200 real-time PCR instrument (Bio-Rad, Germany) in a reaction mixture with a final volume of 50 μl including 25 μl SYBR Premix Ex Taq (Takara, Japan), 1 μl of each primer (10 μM), 4 μl template cDNA, and 19 μl DEPC water under the following conditions: 95 °C for 30 s and 40 cycles of 95 °C for 5 s, 60 °C for 30 s, and 72 °C for 32 s. Each gene was analyzed in triplicate. The GAPDH gene was used as the internal control for normalization (Wang et al. 2016). A no-template control sample with nuclease-free water was used to detect contamination and the degree of dimer formation; no contamination or dimer formation was detected. The relative transcript level in each tissue was calculated using the comparative 2^−∆∆CT^ method (Livak and Schmittgen. 2001). All of the Ct values were normalized to the internal control gene in the same tissues. Then, in order to describe the relative expression levels of V1R genes in all the ten mentioned tissues of *C. nasus* more clearly and directly, the relative expression levels of V1R genes in different issues were presented as fold changes by comparing their normalized Ct values relative to the normalized Ct values of the same genes in the tissue with the lowest normalized Ct value. For investigation into the differential expression profiles of the V1R genes, the V1R genes in the olfactory rosettes of *C. nasus* from the Jingjiang section of the Yangtze River (JJOR) were presented as fold changes compared to the normalized Ct values of the same genes in the olfactory rosettes of *C. nasus* from Poyanghu Lake (PYOR). All of the target genes, the internal control gene, and the no-template control were evaluated in triplicate to prevent technical error. The Student’s *t* test was used to determine significant differences among the treatments at *p* < 0.05. All of the data were expressed as mean ± standard deviation (S.D.) in this study.

## Results and discussion

### Identification of six orthologous V1R-like genes from other teleost fish in *C. nasus*

According to the methods mentioned above, all the six V1R genes reported in other fish (Saraiva and Korsching 2007; Pfister et al. 2007; Ota et al. 2012; Johnstone et al. 2012) were identified in *C. nasus* (Supplementary Text S5, Supplementary Text S6 and Supplementary Fig. S7). The nucleotide sequences were deposited in GenBank (GenBank ID: KP260935–KP260940).

### Genetic structures of the V1R genes

**Fig. 2:**
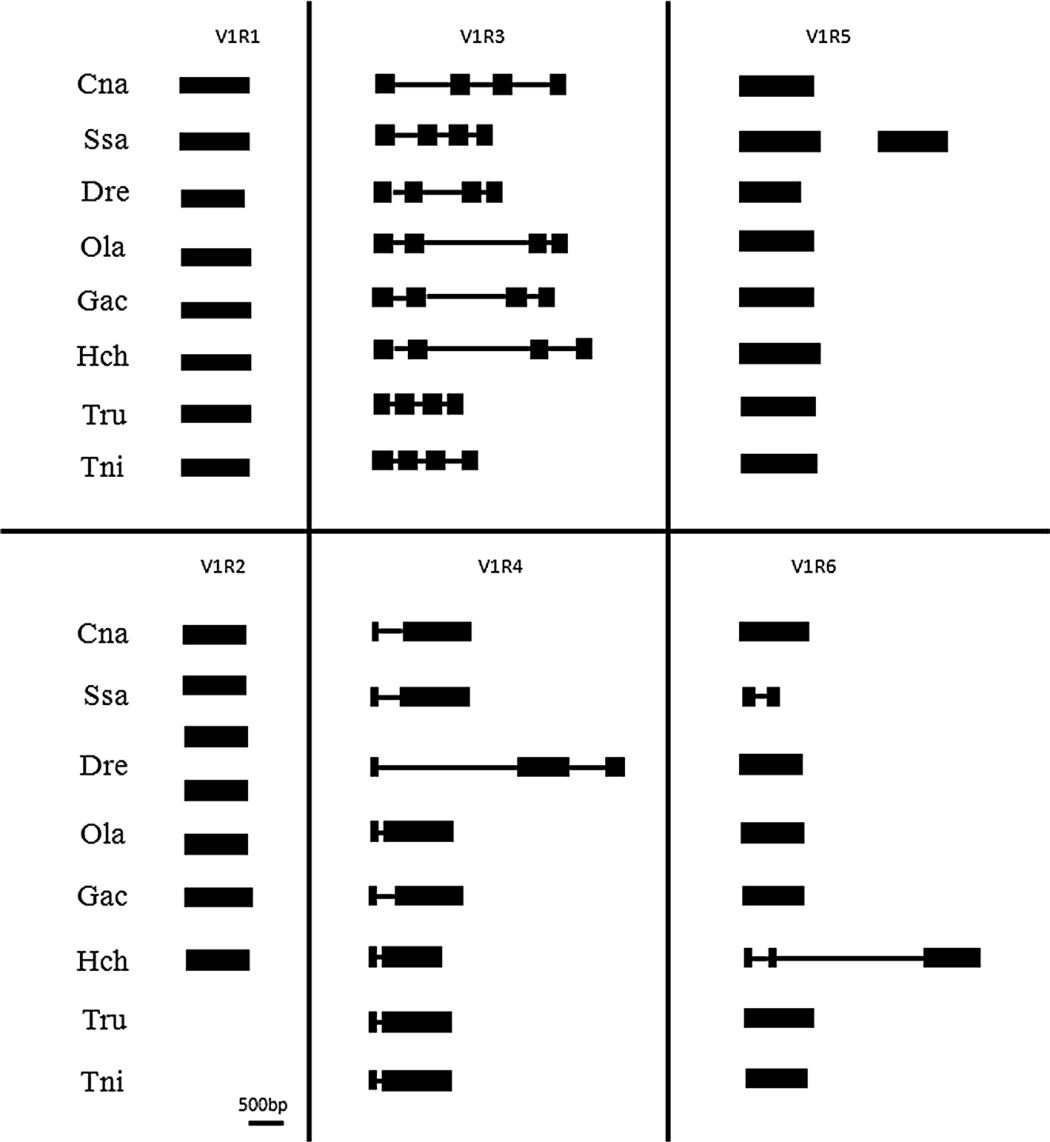
Schematic representation of the *Coilia nasus* (Cna), *Salmo salar* (Ssa), *Danio rerio* (Dre), *Oryzias latipes* (Ola), *Gasterosteus aculeatus* (Gac), *Takifugu rubripes* (Tru), *Tetraodon nigroviridis* (Tni), and *Haplochromis chilotes* (Hch) V1R gene structures (Saraiva and Korsching 2007; Ota et al. 2012; Johnstone et al. 2012). The *black boxes* represent the exons, and the *black lines* represent the introns

The genetic structures of the V1R genes in *C. nasus* were identified using TBLASTN search analysis in the NCBI database (http://www.ncbi.nlm.nih.gov/). The V1R1, V1R2, V1R5, and V1R6 genes possessed only one exon without an intron, the V1R3 gene had four exons with three introns, and the V1R4 gene had two exons with one intron between them; these results were in accordance with other published results(Saraiva and Korsching 2007; Pfister et al. 2007; Ota et al. 2012; Johnstone et al. 2012). However, the intron length of the V1R3 and V1R4 genes differed from the published results from other species (Fig. 2) (Saraiva and Korsching 2007; Pfister et al. 2007; Ota et al. 2012; Johnstone et al. 2012). The detection of highly conserved genetic structures may imply that the V1R genes play an important role in fish (Saraiva and Korsching 2007).

### A conserved region in the internal sequences of V1R1–V1R2 in teleost fish

**Fig. 3:**
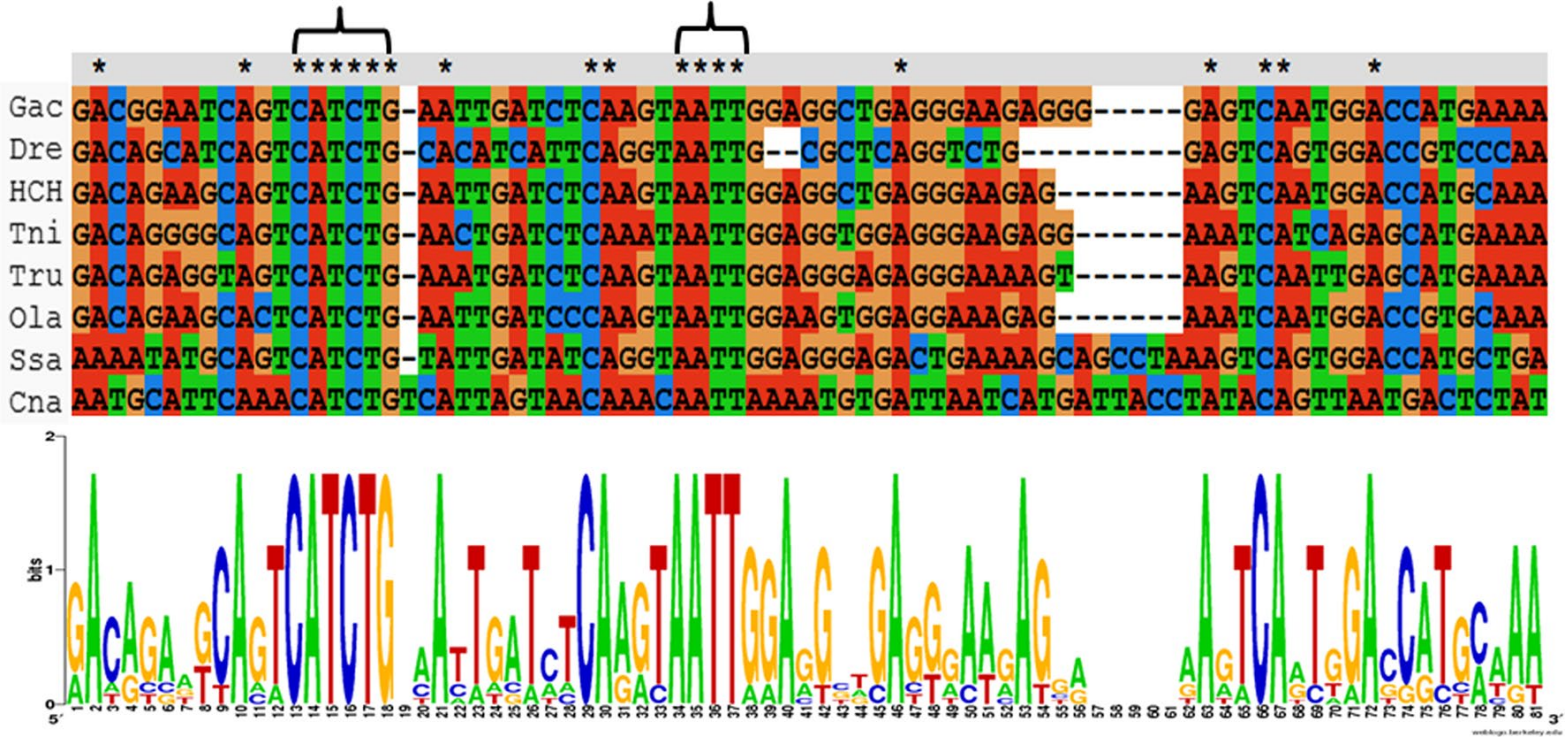
Sequence alignment of the conserved noncoding sequence located in the internal region of the V1R1–V1R2 coding region from *Coilia nasus* (Cna), *Salmo salar* (Ssa), *Danio rerio* (Dre), *Oryzias latipes* (Ola), *Gasterosteus aculeatus* (Gac), *Takifugu rubripes* (Tru), *Tetraodon nigroviridis* (Tni), and *Haplochromis chilotes* (Hch). Two highly conserved motifs are indicated by *brackets*

The V1R genes in mice are characterized by an unusual conservation in noncoding sequences between family members, including the transcribed and non-transcribed regions (Lane et al. 2002). In the 5′-UTR (untranslated region) of V1R1 and V1R2, we identified a conserved segment of 74 bp located approximately 270 bp upstream of the coding sequence of the V1R2 gene (Fig. 3). In the published results, the conserved region is usually located adjacent to the V1R1 genes in the V1R1 5′-UTR (Pfister et al. 2007; Ota et al. 2012). However, in *C. nasus*, the conserved region was located upstream of the V1R2 gene instead of V1R1. The conserved region may indicate the need for a transcriptional or translational regulatory element that acts on fish V1R1 genes and possibly also on V1R2 (Pfister et al. 2007). The genomic organization of the fish V1R1 and V1R2 genes is “head to head,” with an approximately 1- to 3-kb DNA sequence separating the coding sequences of the two genes (Saraiva and Korsching 2007; Pfister et al. 2007; Ota et al. 2012; Johnstone et al. 2012). This arrangement results in a short space between the two UTRs and two promoters whose transcriptional direction is inverted, rendering it easily amenable to genetic manipulation (Pfister et al. 2007). This conservation implies the importance of these two motifs for the transcription of V1R2 or V1R1 (Pfister et al. 2007) and will be an interesting topic for future research. Interestingly, TAATTG is the binding site of the LIM-homeodomain protein Lhx2 in mice, which regulates the expression of the olfactory receptor genes (Hirota and Mombaerts. 2004). The unique conserved motifs CATCTG and AATT may have occurred as a result of the difference in the structure of the LIM-homeodomain protein Lhx2 in *C. nasus*, thereby contributing to differences in the binding site sequences (Ota et al. 2012).

### An expansion of the V1R gene repertoire in *C. nasus*

The V1R gene family is highly conserved in the analyzed teleost fish, where a maximum of seven members have been identified (Pfister and Rodriguez 2005; Saraiva and Korsching 2007; Shi and Zhang 2007; Pfister et al. 2007). However, an expansion event of V1R genes was detected in the genome of *C. nasus*.

**Fig. 4:**
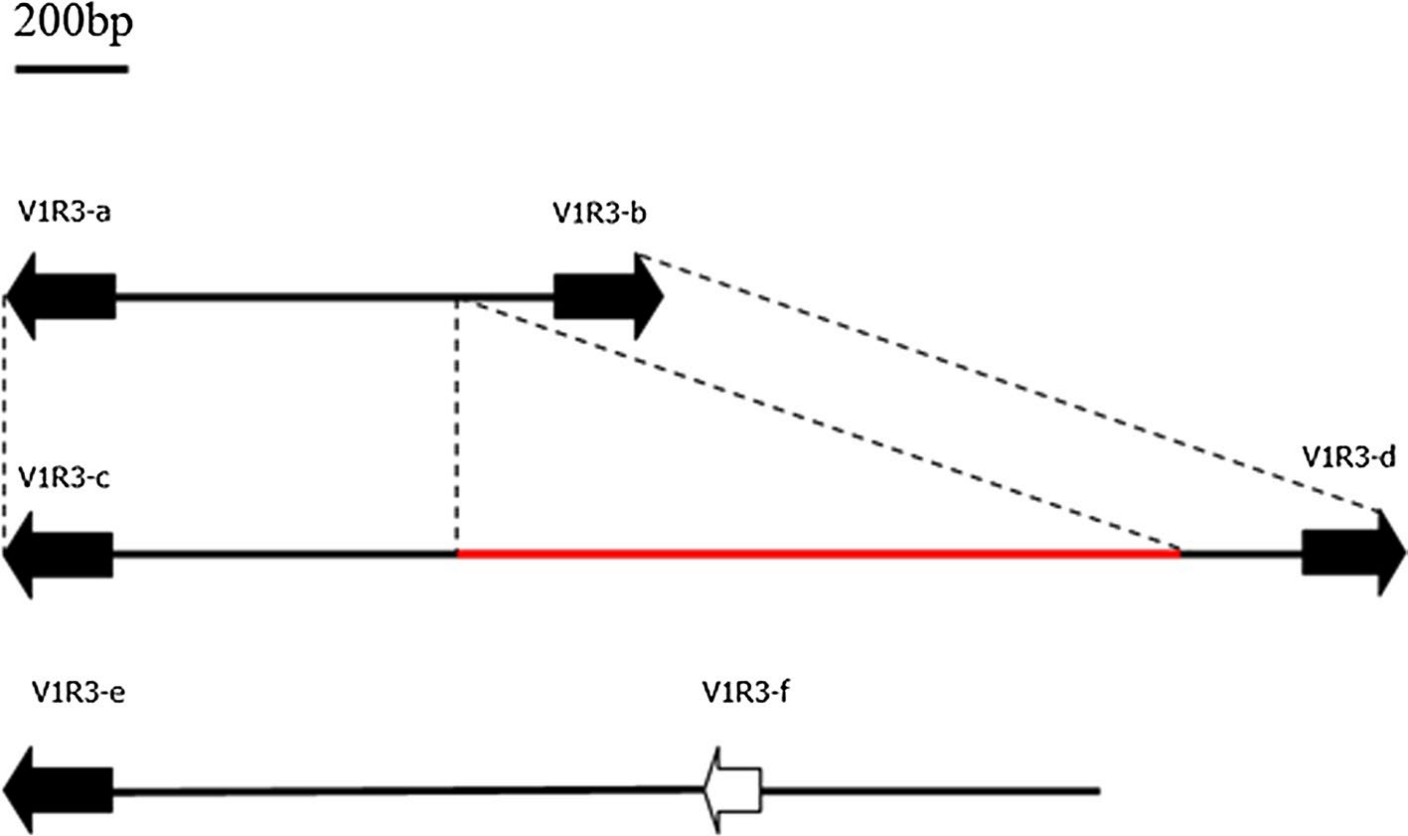
Genomic arrangement of the V1R3 genes in the genome of *Coilia nasus*. Putative functional genes are represented by the *black filled rectangles*, pseudogenes are represented by the *white rectangles*, and the *thick line* represents the intergenic distance between two members of a gene pair. All sequences are drawn to scale

Intriguingly, we identified four different sequences containing regions that were annotated as the 3′-regions of the V1R3 gene (V1R3-3000-7, V1R3-3000-12, V1R3-4000-33, and V1R3-4000-26) from amplification in this study (Supplementary Text S8). Using TBLASTN in the NCBI database (http://www.ncbi.nlm.nih.gov/), two sequences (V1R3-3000-7 and V1R3-3000-12) were identified that contained V1R3 genes (V1R3-a and V1R3-b in V1R3-3000-7 and V1R3-c and V1R3-d in V1R3-3000-12); these genes were arranged in a “tail-to-tail” genomic organization (Fig. 4). In contrast, one V1R3 copy (V1R3-g) was identified in VIR3-4000-26. No stop codon was found inside the CDS (coding sequence) region of the V1R3 fragments in these sequences. Therefore, we identified them as relaxed functional V1R3 genes. In the V1R3-4000-33 sequence, we found two V1R3 genes (V1R3-e and V1R3-f) arranged in a “tail-to-head” genomic organization (Fig. 4, Supplementary Text S8). V1R3-e was identified as a putative functional gene with no stop codon exists inside the CDS region, while the V1R3-f was identified as a pseudogene due to a premature stop codon in its CDS region.

One sequence containing regions that were annotated as the 5′-region of the V1R3 genes was identified from amplification (V1R3-2000-24) (Supplementary Text S8). The V1R3-2000-24, V1R3-Internal, and V1R3-3000-7 sequences were spliced together via sequence assembly with the BIOEDIT 6.0.7 software (Hall 1999).

Based on these results, six putative functional V1R3 genes and one putative pseudogene were identified (Table 1). Therefore, we may predict that some genomic clusters of V1R3 genes exist in pairs in the genome of *C. nasus*. We detected three transcripts of V1R3 through manual searching of the transcriptome data from the *C. nasus* epithelium (Zhu et al. 2014) (Supplementary Text S2); this finding was in accordance with the existence of multiple copies of the V1R3 genes in the genome of *C. nasus*. Moreover, the sequences with the names Unigene55097_All and Unigene18101_All from the transcriptome data were annotated as the 3′-regions of the V1R3 gene (Supplementary Text S2). By aligning these transcript sequences to the identified DNA sequences mentioned above, we found that Unigene55097_All was highly similar with V1R3-b and V1R3-d, while Unigene18101_All was highly similar with V1R3-a, V1R3-c, and V1R3-e.

A novel insight into fish migratory genes may be gained from this result. According to previous reports, there is only one copy of the V1R3 and V1R5 genes in the genome of species that have been examined to date, such as *Danio rerio*, *Oryzias latipes*, *Gasterosteus aculeatus*, *T. rubripes*, *T. nigroviridis*, and *Haplochromis chilotes* (Pfister and Rodriguez 2005; Saraiva and Korsching 2007; Pfister et al. 2007; Ota et al. 2012), which are non-migratory fish. However, the exception is the genome of *S. salar*, which contains two copies of the V1R3 and V1R5 genes. V1R3-a, V1R3-b, V1R5a, and V1R5b were identified through data mining from the genome (Johnstone et al. 2012). It is possible that the lack of known V1R3 paralogs in the examined species may be due to a lower experimental effort in those comparative studies. If the results are correct, however, species-specific gene expansions may be used in the study of species-specific recognition behavior.

Interestingly, both *S. salar* and *C. nasus* have the ability to perform spawning migration behavior. The presence of the same expansion allows us to predict that the V1R3 gene has a relationship with the orientating function during the spawning migration. Some members of the expanded V1R3 genes may have gained novel functions that facilitate the ability of *C. nasus* to adapt to the long-distance spawning migration.

Additionally, when aligning the sequences of V1R3-3000-7 (V1R3-3-a/V1R3-3-b) and V1R3-3000-12 (V1R3-3-c/V1R3-3-d), we found that the two sequences were similar with high identity (just 5-bp difference), with a indel of 1325-bp sequence in the UTR regions (Supplementary Fig. S9). Among the 5-bp differences, four were located in the UTR regions and one in the CDS region. Local gene duplications might have been occurred in the genome of *C. nasus* to give rise to V1R3-3-a/V1R3-3-b and V1R3-3-c/V1R3-3-d.

Based on these results, we may conclude that the ancestor of these fish harbored one V1R3 gene, because nearly all of the species that have been investigated possess only one V1R3 gene (Saraiva and Korsching 2007). Therefore, we hypothesized that expansion events of the V1R3 genes may have occurred during the evolutionary history of *C. nasus*. These events may have resulted from local genome duplications.

### Phylogenetic analysis of orthologous V1R genes among teleost fish

**Fig. 5:**
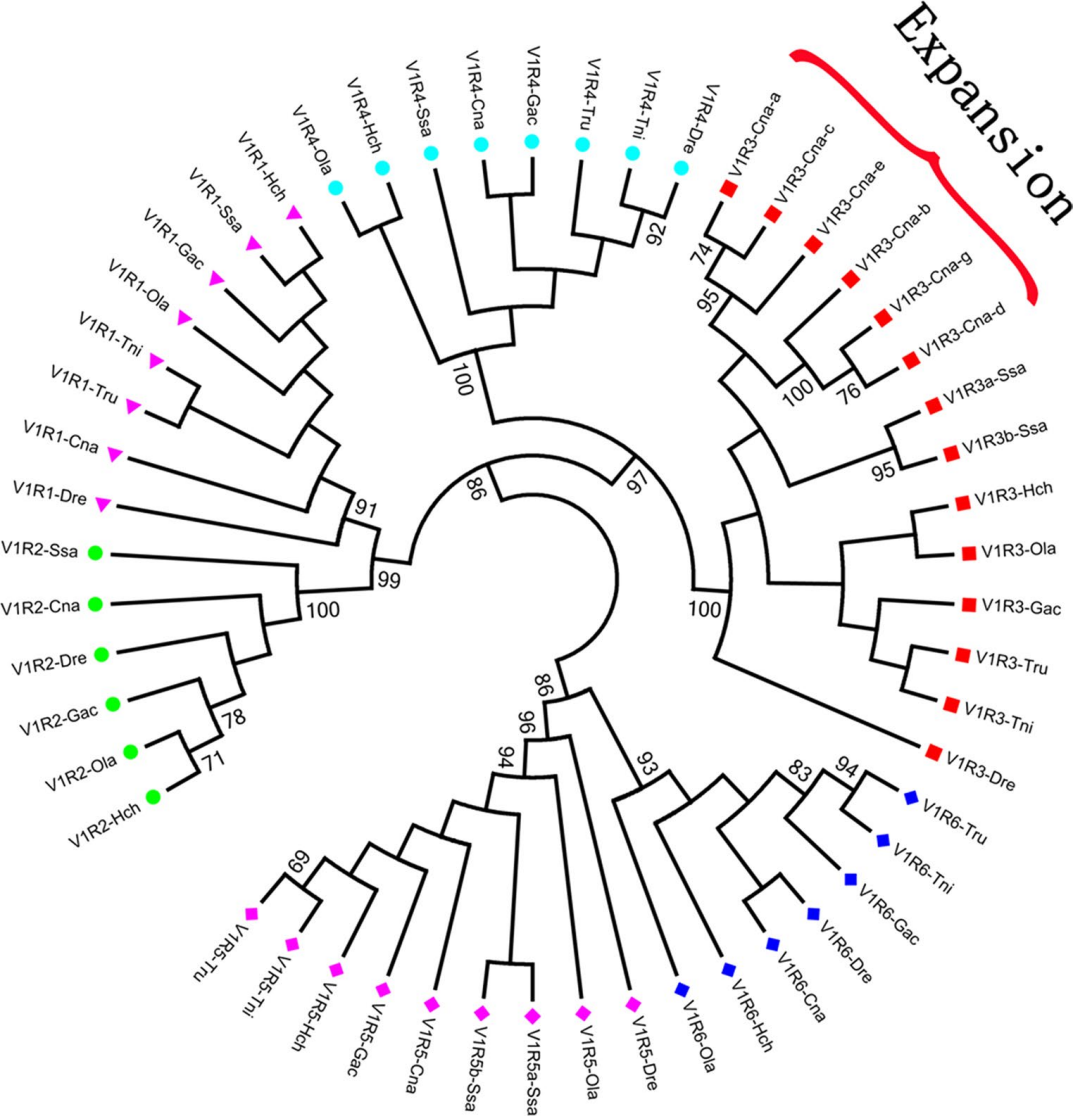
Neighbor-joining tree of previously identified teleost V1R receptors. This tree was constructed with the MEGA 5.0 software using amino acid sequences that were predicted and translated from V1R nucleotide sequences. The taxon names of different V1Rs in every species are shown in different colors. Bootstrap probabilities are indicated at major branch points. The *red bracket* shows the expansion of the V1R3 gene in *Coilia nasus*. Species abbreviations include Ola, *Oryzias latipes*; Gac, *Gasterosteus aculeatus*; Tru, *Takifugu rubripes*; Tni, *Tetraodon nigroviridis*; Dre, *Danio rerio*; Hch, *Haplochromis chilotes*; Ssa, *Salmo salar*; and Cna, *Coilia nasus*

To investigate the functions of the identified V1R genes, we aligned the putative amino acid sequences of all of the V1R receptors from the above-mentioned fish and all of the V1R genes identified in this study to construct a phylogenetic tree. Figure 5 shows an unrooted neighbor-joining tree constructed from all of the putatively functional teleost V1R genes.

Based on this phylogenetic tree, the V1R receptors from teleost fish were subdivided into three pairs: V1R1–V1R2, V1R3–V1R4, and V1R5–V1R6. The neighbor-joining tree suggested the monophyly of each V1R gene family through near-maximal bootstrap probabilities. Orthologs of the six V1R genes could be unambiguously identified (Saraiva and Korsching 2007; Ota et al. 2012). This result confirmed that the V1R genes of *C. nasus* isolated in the present work are all orthologs of the six corresponding known teleost V1R genes. From the tree, we can predict that the genesis of the three clades probably preceded the genesis of the V1R gene pairs (Saraiva and Korsching 2007).

### Repetitive elements in the V1R Loci in *C. nasus*

By aligning the copies with the Repeatmasker online software (http://www.repeatmasker.org/), the simple repeat sequences (ACAC)n and (TGTTAA)n were detected between V1R3-a and V1R3-b. Moreover, the simple repeat sequences (ACAC)n and (TGTTAA)n and (AATAG)n were detected between V1R3-c and V1R3-d, while (ACAC)n, and (TGTTAA)n and (TCTC)n were detected in the flanking region of V1R3-e. Finally, the simple repeat sequence (AGCGGC)n was found in the flanking region of the V1R4 gene.

A copy of SINE (short interspersed nucleotide element), a type of retrotransposon widely distributed in various eukaryotic genomes (Kramerov and Vassetzky 2011), was found to be inserted into the 3′-flanking region of the V1R6 genes. Through the sequence annotation in the NCBI dataset, we found that the SINE copy belonged to the tRNA-derived Cn-SINEs, which are a SINE family that was previously isolated from *C. nasus* by our laboratory (Liu et al. 2012).

### Polymorphism of V1R3 gene in different populations of *C. nasus*

Different products in the different populations of *C. nasus* described above were detected via PCR with the V1R3IF and V1R3IR primers (Supplementary Table S3; Supplementary Fig. S10; Supplementary Text S11). Interestingly, a deletion in the V1R3 genes was found in some of the individuals from the populations of Poyanghu Lake and Dongtinghu Lake.

When some of the bands were purified and ligated into vectors for sequencing, some interesting results were obtained (Supplementary Text S11). Several insertions were found in the DNA sequence alignments of the populations from Dongtinghu Lake and Poyanghu Lake, including (GAGTCACACTACCAGTGCTGCCAAGGT)n and (TACCAGTGCTGCTAAGGTGCGTCAC)n. The numbers of the repeats varied in different populations of *C. nasus* (Supplementary Text S11). However, Supplementary Fig. S10 shows that 75 % of the individuals from the marine and anadromous populations possessed two bands, while 70 % of the individuals from the landlocked and non-anadromous populations possessed a single band. While there is not enough evidence to definitively use this marker to discriminate between populations (Zhu et al. 2014), this finding represents an interesting trend that may be used to address this problem.

These above results may indicate that the degree of differentiation of the populations of Poyanghu Lake and Dongtinghu Lake from the Jingjiang population was higher compared to the Taihu Lake population and Zhoushan population. One explanation may be that the differentiation of the landlocked *C. nasus* and marine *C. nasus* from the anadromous *C. nasus* occurred later than the divergence of non-anadromous *C. nasus* from anadromous *C. nasus*.

In addition, copy number variation (CNV) in V1R3 gene among individuals of *C. nasus* is also indicated, given the polymorphism of V1R3 gene in the different populations of *C. nasus* (Supplementary Fig. S10; Supplementary Text S11). CNVs, which are interspersed widely in the genome, make great contribution to genome evolution and population-based genetic variation (Conrad et al. 2010; Redon et al. 2006; Reymond et al. 2007; Sebat et al. 2004; Iafrate et al. 2004; Nozawa et al. 2007). The variation in V1R3 gene copy number is likely to be accounted for by olfactory requirements for the *C. nasus* to adapt to their specific living environments. This result may suggest an interesting pattern of local adaptation by CNV of *C. nasus*.

### Expression profiles of the putative *C. nasus* V1R genes in different tissues

**Fig. 6:**
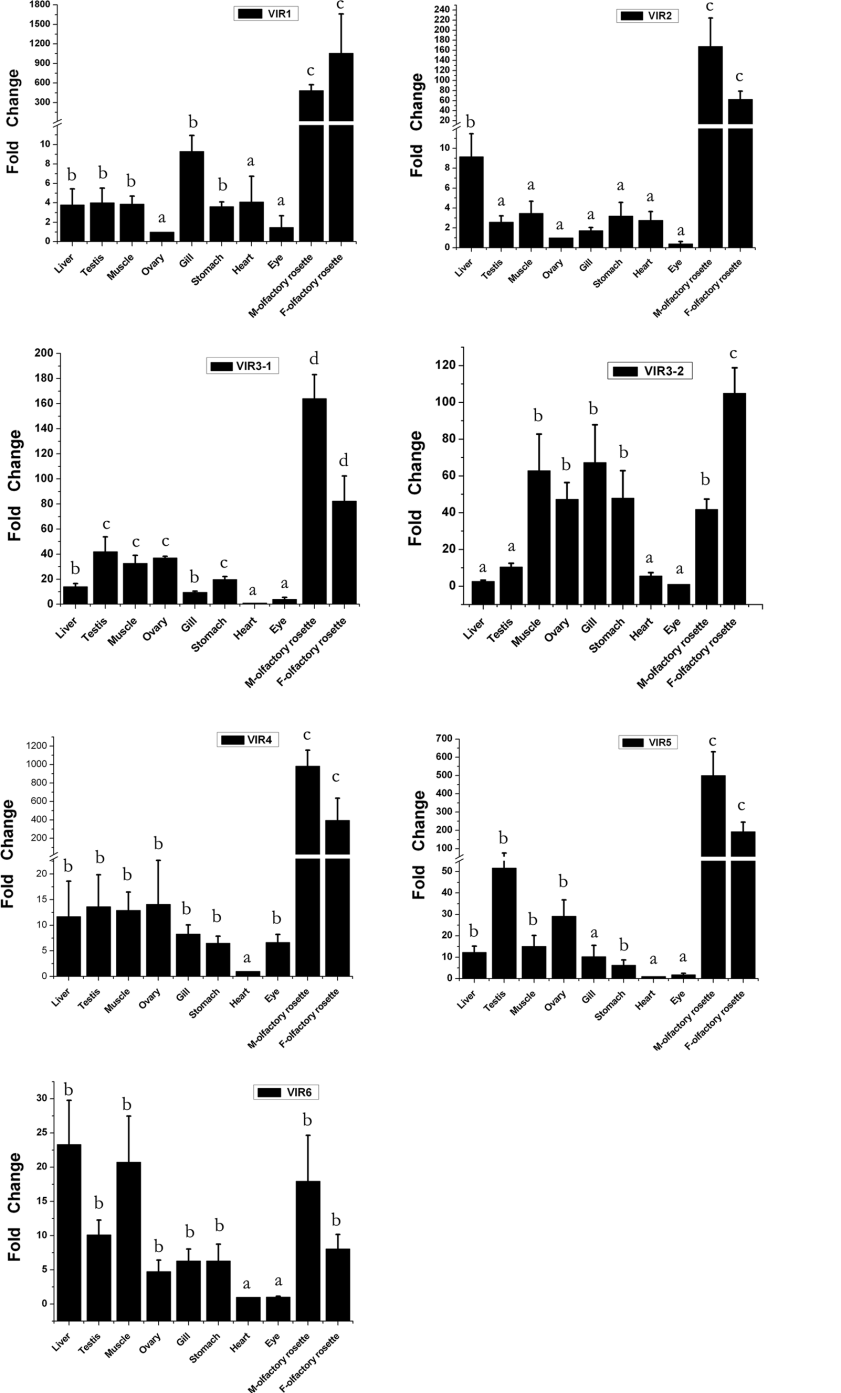
Expression levels of *Coilia nasus* V1R genes determined by qPCR in different tissues from the anadromous form collected from the Yangtze River. The relative expression indicates the level of V1R gene transcripts normalized to the internal GAPDH standard. The relative expression levels of all the V1R genes were presented as fold changes through comparing their normalized Ct values in different tissues relative to the normalized Ct values of the same genes in tissues with the lowest normalized Ct value. The *bars* with different letters (a, b, c) indicate statistically significant differences (*p* ≤ 0.05) between mean expression levels

Any functional olfactory receptor is expected to be specifically expressed in the olfactory receptor neurons situated in the olfactory epithelium (Buck and Axel 1991; Johnstone et al. 2009). To investigate the functions of the identified *C. nasus* V1Rs, we examined the expression profiles of the V1R transcripts in various adult tissues from anadromous *C. nasus* migrants collected from the Yangtze River in Jingjing, Jiangsu Province, through qPCR (Fig. 6, Supplementary Table S4).

Five V1R genes (V1R1, V1R2, V1R3-1, V1R4, and V1R5) were primarily expressed in the female and male olfactory rosettes compared to other tissues (Fig. 6); this result was consistent with their assignment as functional olfactory receptors (Buck and Axel 1991; Johnstone et al. 2009). The V1R gene expressions were also detected in other tissues, although most of them have a relatively lower expression level compared to olfactory rosettes, suggesting that these genes may also have non-olfactory functions in other tissues of *C. nasus*.

In addition, it is worth noting that five V1R genes (V1R2, V1R3-1, V1R4, V1R5, and V1R6) were expressed at a higher level in the male olfactory rosettes than in the female rosette, while two V1R genes (V1R1 and V1R3-2) showed a higher expression level in the female rosettes than in the male rosettes. The sex-biased expression differences suggest that such V1Rs may be putative receptors of chemical cues used for sex-specific behaviors.

### Possible contribution of V1R-mediated olfaction to spawning migration behavior in *C. nasus*

In spring every year, mature anadromous *C. nasus* migrants undergo the long-distance migration from the ocean coast to the exorheic rivers to spawn in the affiliated lakes (Zhu et al. 2014; Yuan et al. 1980). A pheromone hypothesis has been proposed for *S. alpinus* and *S. salar* that assumes that the juvenile salmon in a stream releases population-specific odors that guide homing adults (Nordeng 1971, 1977). We hypothesized that pheromones might be used as odorant cues by the *C. nasus* migrants to guide their travels during anadromous migration. Therefore, we investigated gene expression levels between the anadromous fish collected from the Jingjiang section of the Yangtze River and the non-anadromous population of *C. nasus* collected from Poyanghu Lake using qPCR analysis.

**Fig. 7:**
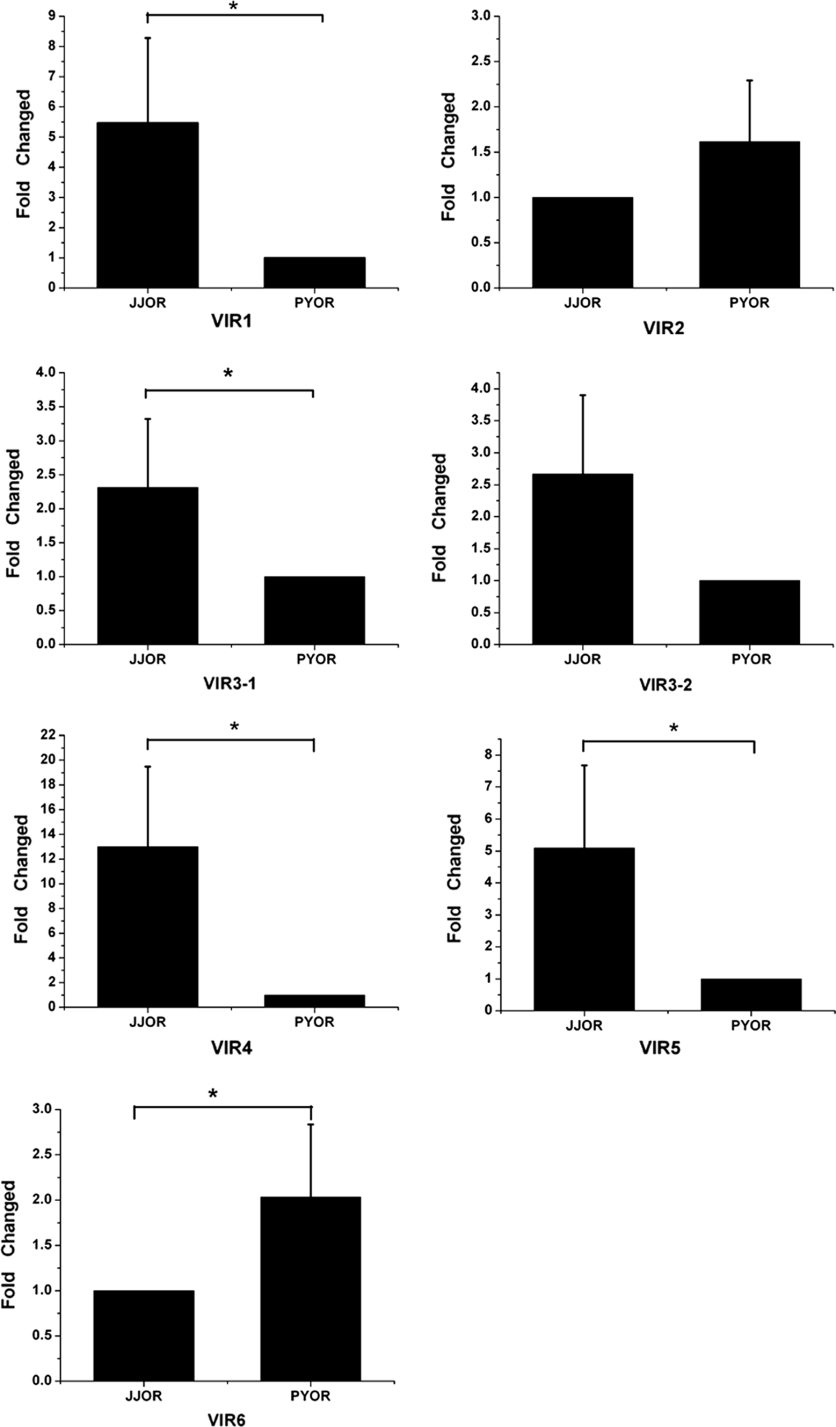
qPCR analysis of the differential mRNA expression levels of V1R genes in anadromous and non-anadromous *Coilia nasus*. JJOR represents the olfactory rosettes from the anadromous migrants collected from the Yangtze River, and PYOR represents the olfactory rosettes from non-anadromous migrants collected from Poyanghu Lake. The results were normalized to the internal GAPDH standard and averaged. The average expression level of each V1R gene in JJOR or PYOR was normalized to 1.0 for graphing. Differences are considered to be statistically significant at the *p* < 0.05 level and are marked “*”

Five genes (V1R1, V1R3-1, V1R3-2, V1R4, and V1R5) were upregulated in the olfactory rosettes of anadromous *C. nasus* compared to the non-anadromous *C. nasus* (Fig. 7), while two V1R genes (V1R2 and V1R6) were downregulated in anadromous *C. nasus* compared to non-anadromous *C. nasus*.

These expression patterns indicate the possibility of a physiological change in the olfactory system of anadromous fish compared with non-anadromous fish. During the spawning migration, anadromous fish may detect odorant cues in the river through their olfaction systems. The detection of these cues may demand an increase in the expression level of a certain set of receptors in order to bind and discriminate all of the odorants in the waters. In contrast, non-anadromous adults may not need it.

Thus, the upregulated V1R1, V1R3-1, V1R3-2, V1R4, and V1R5 genes may be involved in the spawning migration of *C. nasus* and should receive more attention in future investigations into migration-related genes.

The V1Rs were predicted to detect pheromones (Boschat et al. 2002; Del Punta et al. 2002; Isogai et al. 2011; Behrens et al. 2014). Therefore, the pheromone receptors may be involved in the spawning migration of *C. nasus*. Our present results on V1R evolution are thus compatible with the hypothesis that *C. nasus* larvae in the freshwater lakes adjacent to the Yangtze River release pheromones to guide anadromous *C. nasus* adults to those lakes to spawn via migration up the Yangtze River.

Based on these results, we propose that the larval *C.nasus* in the fresh lakes adjacent to the Yangtze River release pheromones to guide anadromous *C. nasus* adults to those lakes to spawn via migration up the Yangtze River.

## Conclusion

This study represents a first step toward elucidating the olfactory communication system of *C. nasus* at the genetic and molecular levels. Altogether, we have identified the whole family of V1R olfactory receptor genes in *C. nasus* that are highly conserved in fish species. We also detected the expansion events of the V1R3 genes in *C. nasus*. To date, *C. nasus*, which has the ability to perform anadromous migration, possesses the largest V1R repertoire in teleost fish. The discovery of the same V1R3 gene expansion event in *C. nasus* and *S. salar* provides us with a novel insight into research of migratory fish. Moreover, the divergence of V1R3 genetic structures in different populations of *C. nasus* indicates the CNV in V1R3 gene among individuals of *C. nasus*. Besides, we identified three V1R genes that are differentially expressed between anadromous and non-anadromous *C. nasus*. Therefore, we hypothesize that these V1R genes play an important role in the detection of olfactory cues from river water during the spawning migration of anadromous *C. nasus*.

## Electronic supplementary material

Below is the link to the electronic supplementary material.

Supplementary Table S1. PCR primers used for the isolation of the six V1R genes of *Coilia nasus* (PDF 337 kb)

Supplementary Text S2. Transcript sequences of V1Rs in the *Coilia nasus* transcriptomes (PDF 227 kb)

Supplementary Table S3. PCR primers used to detect polymorphisms in the V1R genes in different populations of *Coilia nasus* (PDF 315 kb)

Supplementary Table S4. Primers used for real-time quantitative PCR of the V1R genes of the anadromous and non-anadromous forms of *Coilia nasus* (PDF 354 kb)

Supplementary Text S5. Nucleotide sequences of V1Rs in *Coilia nasus* (PDF 317 kb)

Supplementary Text S6. Amino acid sequences of V1Rs in *Coilia nasus* (PDF 302 kb)

Supplementary Fig. S7. Amino acid structures of V1Rs in *Coilia nasus* (PDF 1029 kb)

Supplementary Text S8. Nucleotide sequences of V1R3 genes in *Coilia nasus* (PDF 227 kb)

Supplementary Fig. S9. The alignment of V1R3-3000-7 (V1R3-3-a/V1R3-3-b) and V1R3-3000-12 (V1R3-3-c/V1R3-3-d) (PDF 1309 kb)

Supplementary Fig. S10. Polymorphism in V1R genes from different populations of *Coilia nasus* collected from the Jingjiang section of the Yangtze River, the waters of Zhoushan, the Taihu Lake, the Poyanghu Lake, and the Dongtinghu Lake. The M represents the DNA marker. Lanes 1–10 are from the Jingjiang population, 11–20 are from the Zhoushan population, 21–30 are from the Taihu Lake population, 31–40 are from the Poyanghu Lake population, and 41–50 are from the Dongtinghu lake population. Lane 0 is the negative control with sterile water used as the template (PDF 479 kb)

Supplementary Text S11. Nucleotide sequences of the internal region of the V1R3 gene in the populations from Jingjiang, Zhoushan, Taihu Lake, Poyanghu Lake, and Dongtinghu Lake (PDF 302 kb)
