# Supplementary material for: Identification of a uniquely expanded V1R (ORA) gene family in the Japanese grenadier anchovy (Coilia nasus)
Source: Mar Biol. 2016 May 2;163:126. doi: 10.1007/s00227-016-2896-9 (PMC4853444; doi:10.1007/s00227-016-2896-9)
Supplement: Supplementary file 1 — Supplementary Table S1. PCR primers used for the isolation of the six V1R genes of Coilia nasus (PDF 337 kb) [file 227_2016_2896_MOESM1_ESM.pdf]

Electronic Supplementary Material

Identification of a uniquely expanded V1R (ORA) gene family in the Japanese grenadier anchovy (*Coilia nasus*)

Guoli Zhu[a], Wenqiao Tang[a *], Liangjiang Wang[b], Cong Wang[a], Xiaomei Wang[a]

[a] College of Fisheries and Life Science, Shanghai Ocean University, Shanghai, China

[b] Department of Genetics and Biochemistry, Clemson University, Clemson, South Carolina, United States of America

* Corresponding author: College of Fisheries and Life Science, Shanghai Ocean University, Shanghai, China; phone: + 86-21-61900425; Email: wqtang@shou.edu.cn

Supplementary Table S1. PCR primers used for the isolation of the six V1R genes of *Coilia* nasus.

Oligo Name	Sequence (5' to 3')
**V1R1-F**	TGTTATTTGTAAGTTGCTGCCTATGTGGA
**V1R1-R**	AATTTTATTATTGTGGACCGGGTTTTGAT
**V1R1-5'-SP1**	CACCGCTCCGTTAGCATCCACTGAAA
**V1R1-5'-SP2**	ACCACCATCAAGTTAGCAAAAGCCAG
**V1R1-5'-SP3**	CAGTGAGAGATAGATAGAGCAGCCCG
**V1R2-F**	CTGGTTTGGGCATTTTGGGAA
**V1R2-R**	CCTTGGAGGGTTGGTGGTTTG
**V1R2-5'-SP1**	ATAACAAGATGAGAACCACACCCGCA
**V1R2-5'-SP2**	GCATAAGAGCCTGGAAGACACTGAGC
**V1R2-5'-SP3**	GAGGGCACGGGAAATACGGTAGGAGT
**V1R3m-e3F**	CTGGGCTGTRTSTGGAACTT
**V1R3m-e4R**	GGNGCCTCKGTTGTAGTTGA
**V1R3-5'-SP1**	TGGTGCCCCAGGAGGAGATGAAGAG

**V1R3-5'-SP2**	CACCCTCCTGATGACGGGCACCTC
**V1R3-5'-SP3**	TGTGGGCGTAGAGTGTCAGCAGCGAA
**V1R3-3'-SP1**	TGGGTTCGCTGCTGACACTCTA
**V1R3-3'-SP2**	TGCCCGTCATCAGGAGGGTGCCAG
**V1R3-3'-SP3**	TGCTCTTCATCTCCTCCTGGGGC
**V1R4-3'-SP1**	CTGACTGTTAGTGAGTTACACCTGCC
**V1R4m-F**	CMGAGTCTTCATGCTGCTGTG
**V1R4m-R**	CTGTYGTGGTTGTARTACGTCAC
**V1R4L-F**	CACCTGCCAGATATGGCTAACAGAGA
**V1R4L-R**	GTACAGCTGATCACCATGAGCTCCAC
**V1R4-3'-SP2**	CATCTCACACCCCTCTCCCATTCTTT
**V1R4-3'-SP3**	TTGAGTGTGCGGATTGTCCTGTCTGT
**V1R4-3'-SP4**	TGGGAGTTCCCGACTGAGGAGCAG
**V1R5-F**	AGAATGGATGCAGAGGGGTGGGT
**V1R5-R**	CTGATGGGCAGTGGGAGAAGGAA
**V1R5-3'-SP1**	CGAATCTTCCGCTTCTGTGCCGACCT

**V1R5-3'-SP2**	ATCTTCTCCACCCTCTTCATCAGCGT
**V1R5-3'-SP3**	AGCATCCCGCATCTCATCTATGTCAC
**V1R5-5'-SP1**	GAACACGCTGATGAAGAGGGTGGAGA
**V1R5-5'-SP2**	ACAGGTCGGCACAGAAGCGGAAGATT
**V1R5-5'-SP3**	ACAGCCAGATTGAGGAATAGAGCGTC
**V1R6m-F**	GTCMTCATCAGCATCTTCCG
**V1R6m-R**	AAGGTCCAGTYCACCWGGAA
**V1R6-3'-SP1**	CTCATCAGCATCTTCCGCTACCAG
**V1R6-3'-SP2**	TGCCACTGTTCAACCACCTCTAC
**V1R6-3'-SP3**	CAACCTGCTGCCCTTGCTCATCG
**V1R6-5'-SP1**	TTTCTTGCCCACAGGTCAGACAGTAG
**V1R6-5'-SP2**	GGTGGTGATGAAGAACTCCATCTCAG
**V1R6-5'-SP3**	ACTTGTAGAGGTGGTTGAACAGTGGG
