# Supplementary material for: Identification of a uniquely expanded V1R (ORA) gene family in the Japanese grenadier anchovy (Coilia nasus)
Source: Mar Biol. 2016 May 2;163:126. doi: 10.1007/s00227-016-2896-9 (PMC4853444; doi:10.1007/s00227-016-2896-9)
Supplement: Supplementary file 2 — Supplementary Text S2. Transcript sequences of V1Rs in the Coilia nasus transcriptomes (PDF 227 kb) [file 227_2016_2896_MOESM2_ESM.pdf]

Electronic Supplementary Material

Identification of a uniquely expanded V1R (ORA) gene family in the Japanese grenadier anchovy (*Coilia nasus*)

Guoli Zhu[a], Wenqiao Tang [a *], Liangjiang Wang [b], Cong Wang[a], Xiaomei Wang[a]

[a] College of Fisheries and Life Science, Shanghai Ocean University, Shanghai, China

[b] Department of Genetics and Biochemistry, Clemson University, Clemson, South Carolina, United States of America

* Corresponding author: College of Fisheries and Life Science, Shanghai Ocean University, Shanghai, China; phone: + 86-21-61900425; Email: wqtang@shou.edu.cn

Supplementary Text S2. Transcript sequences of V1Rs in the *Coilia nasus* transcriptomes.

>Unigene18832_All V1R4 (transcriptome data)

CTGACTGTTAGTGAGTTACACCTGCCAGATATGGCTAACAGAG
ACTCAAACAAGCCCAGCTGATGGAGAGATGTCTGAGGTGCTCA
CGGTAGATGCCATTTTGTTTGGGCTTCTGGTTTTCTCAGGAATC
GTAGGGAACATTCTGGTCATATATGTGGTCTTCGAGTCGGCCA
CGGAGAATTCCTACCGCCACCTCCCTCCCTCCGACGCCATCCT
AGTCAACCTGTCGCTGGCCAACCTGCTGACGTCGCTGTTCCGC
ACCGTGCCCATCTTCATCTCGGACCTGGGCCTGGAGGTGAGCC
TGGCGCCGGGCTGGTGCCGGCTCTTCAT

>CL5470.Contig2_All V1R5-1 (transcriptome data)

GTACATTGCAACCCAACCAGCTGAAGAATGGATGCAGAGGGG
TGGGTCGAGTCTTTTGCCAGGGCTACTATGTGCTTATTGGGTAT
AGTGGGCAACAACTGGCTAGCTTTTAGTTCATTCCCAAGATCC
AAGTCACAGCTGAAGACTAATGACGCTCTATTCGTCAATCTGG
CTGTGTCCAACCTCATCACTAACTACATGGTAGATCTGCCCGA
CACCATGGCAGATTTTGCGGGCCGCTGGTTCATGGGCCTGACC
TACTGTCGAATCTTCCGCTTCTGTGCCGACCTGTCAGAGACCA
GCAGCATCTTCTCCACCCTCTTCATCAGCGTGTTCTGGTACCAG
AAGCTGGTGGGCTCCCTGAAGCGTGGAGGGGCCCCGGTCCGCT
TGGACAACCTGCGTCTAGTCGCTGCCCTTCTGGGAGGAAGCTG
GATGGTGGCTATCGTCTTCAGCATCCCGCATCTCATCTATGTCA
CGATAGAGGAAGGAGATGAACCTGACTGTGTGGATGACTTTCC
TTCTCCCACTGCCCATCAGATCTACGAGATCCTGTACTTGAGCT
TGGCAAATGCCGTTCCTATCACTGGCATCGTCTTCGCCAGCATC
CAGATCGTGGTCACCCTTCTAAAAAATCAGCAACGCATCAGGG
CAACAGGAGCTGGAACAGGTGAGCCTTCACCAGACAAGCCTC
AGGACACTGCAGATACAACTAGTCCAGGTCAAGCTCAAGCAC
AGCATCCAGATCGTGGTCACCCTTCTAAAAAATCAGCAACGCA
TCAGGGCAACAGGAGCTGGAACAGGTGAGCCTTCACCAGACA
AGCCTCAGGACACTGCAGATACAACTAGTCCAGGTCAAGCTCA
AGCA

>CL5470.Contig1_All V1R5-2 (transcriptome data)

GTACATTGCAACCCAACCAGCTGAAGAATGGATGCAGAGGGG
TGGGTCGAGTCTTTTGCCAGGGCTACTATGTGCTTATTGGGTAT
AGTGGGCAACAACTGGCTAGCTTTTAGTTCATTCCCAAGATCC
AAGTCACAGCTGAAGACTAATGACGCTCTATTCGTCAATCTGG
CTGTGTCCAACCTCATCACTAACTACATGGTAGATCTGCCCGA
CACCATGGCAGATTTTGCGGGCCGCTGGTTCATGGGCCTGACC
TACTGTCGAATCTTCCGCTTCTGTGCCGACCTGTCAGAGACCA
GCAGCATCTTCTCCACCCTCTTCATCAGCGTGTTCTGGTACCAG
AAGCTGGTGGGCTCCCTGAAGCGTGGAGGGGCCCCGGTCCGCT
TGGACAACCTGCGTCTAGTCGCTGCCCTTCTGGGAGGAAGCTG
GATGGTGGCTATCGTCTTCAGCATCCCGCATCTCATCTATGTCA
CGATAGAGGAAGGAGATGAACCTGACTGTGTGGATGACTTTCC
TTCTCCCACTGCCCATCAGATCTACGAGATCCTGTACTTGAGCT
TGGCAAATGCCGTTCCTATCACTGGCATCGTCTTCGCCAGCATC
CAGATCGTGGTCACCCTTCTAAAAAATCAGCAACGCATCAGGG
CAACAGGAGCTGGAACAGGTGAGCCTTCACCAGACAAGCCTC
AGGACACTGCAGATACAACTAGTCCAGGTCAAGCTCAAGCAC
AGCAAGGCTCTAATTCTCCATCAGCTCAAGGTCCACCCAGCAG
TGCACCAGTGGTTGCTGTCCAGCAGCAAGCACAGGCAGCACCC
AAAGCACAGGCGAAGGGCAGCCCTGGTGCTGGCGGCCTGGTG
CGGGCGGCCAAGAGTGTGGTGGCTGTGGCCTCCGTGTTTCTAG
TGTGCTGGGTGACCCATCTGCTCCTCCGCATCAGCAGCAACGT
GAAGACGTCCAAGATCGTGGTGGAGGTGGCCAGCTACATCGC
AGCGTCCTACACCAGCATCATCCCCTACATCTTTCTGCACGGTG
TGAAGAAACTCACCTGCAACTGTAGACGGTGAGCTGGAAATG
GTCAGAGCCTGTGGATCACTGACATTCAGCTTGTAGAACAGTG
TTCATTATACAACACTGAGCAGAATTTAAGGTTTATGTAATAT
ATACACATGTACAGTCATGTGTACAGTTGTGTTCATAAGTTTAC
ATACCCTGGCAG

>Unigene12966_All V1R3-1 (transcriptome data)

AGGTGATTCTGGCTCTAATCATGCTCTTCATCTCCTCCTGGGGC
ACCAGTATAATCTCGGTCAACTACTTCAACTATAATCGCGGCA
CATCTACGGAGTTTCTGTTGGTCATCGCTCGCTTCACCAACATC
ACCTTCATTGCGCTGTCACCCATTGTCCTCGCAGTAGGACACG
GACGCCTGCGAGCTGTTCTGAAGTCCCTGCTCACTCACTGACA
GCTGGACATGTCTGAAGGCCTGAAGGGCTGTGGCACTGCATAT
AGTCAGCGTATTCGTTACCATAAATGCAGATAAGCTCACATCT
CAAAACCACCAAAAACTGTCCTTTGGAAACCTTTGAGTGGAGA
TCTTGAGCTGTTTTCAACGCTGAACACATATGCACACACGCAC
GCACGCACGCA

>Unigene55097_All V1R3-2 (transcriptome data)

AGAGGGTGCCGGTGGAGAGGCGTGCTGCCAAGGTGATCCTGG
CCTTAAACATGCTATTCATCACCTCCTGGGGTACCAGTATGATC
TCTATCAACTACTTTAACTACAACCGTGGTCCATCCACGGAGT
ATATGCTGGTCATTGCTCGCTTCGCCAACACCGCGTTCATCGCC
TTCTCCCCTTTTGTGCTAGCGGTCGGACACAGGCATATCAGAG
GTGTACTCAGGTCTCTTCTTATTAACTGAACAAAACCACCGGA
CATTATTGCCCTACAGAGGCATTATTGCCCTACAGAGGCAAAG
TGCAGTCACTACACGAACACTGAAACTCAGAAAAATGCCTTCA
AAGTTGTCTTACTGCCCAGTCAAACATGTTGT

>Unigene116116_All V1R3-3 (transcriptome data)

TCGCATCGCTCCCATGTCCGTGACCAGAGGACCTTCCAAATTC
TTCTTCCTCATCTTCGGCCTGATTTGGTTCCTCAATCTGCTCTAC
TCCATCCCTGCGTTTGTCTTCTCAACCAGCGGGGACAGGAACT
CCACAGAGACCCTGATGTTGGTGAGCAGCACCACACGCCCCCT
GCTGGGCTGCGTGTGGAACTTCCCCACTGTCTACA

>Unigene18101_All V1R2 (transcriptome data)

GTCAAATGCATGCAGGATGAACATTTTCTGTATTACAGAAAAC
ATTTACAGATGATAGTAATTCTAAGTTATTTATCTCAATTTCCT
TACATTTCTGTCTTTCTAATCTTAGTTGCATAATTCCTAGGTCA
GCGTGCTCTGTTGCAATGGACCTGTGCCTGTCTATTAAGGGTGT
CTCCTTCCTCTTGCAGACTGGTTTGGGCATTTTGGGAAATGTTT
TGGTGCTGTTGGCTTACATTCAAATTGTATGCCTAGAGCCGCAT
TTGCTACCTGTAGACATAATCCTGTGCCATCTGGCCTTCACTAA
CCTGATGTTACTGCTGACTCGATGTGTACCTCAGACCATGACT
GTGTTTGGCTTACGTAATCTCCTGAATGATGCAGGGTGCAAGG
TGGTTATCTACTCCTACCGTATTTCCCGTGCCCTCTCCGTCTGT
ATTACCTGCATGCTCAGTGTCTTCCAGGCTCTTATGCTGGCCCC
AGCTAAGCCTTTTTGGGTCAGGTTGAAGACGAGACTACCTAGC
CTTGTCATCCCAACATTTGCTGCCCTCTGGTTCATCAACATGGC
TGTGTGCATTGCTGCGCCCTTCTTCTCCATCGCACCTAAAAATG
GCACGGTGCCAGCTTTCACACTCAACCTAGGATTTTGTCATGT
GGACTTTCGTGATAACCTATCATATGTTATCAATGGGGTAGCT
GTATCAACACGTGACTTCATCT

TTGTAGGGTTTATGCTGGGCTCCAGTGGGTACATACTGGTGGT
GCTCCATCAGCATGCCCAGAAGGCACACAGTATACGACGAAG
TCAGGCTGGCGCAGCCATGGAAACACGTGCAGCAAATACAGT
GGTCACATTAGTTACGCTGTATGCAGTATTCTTTGGCATAGACA
ATGTTATATGGATCTACATGCTGACGGTGGACCAAGTACCCCC
CCTGGTGGCAGACATGCGGGTGTGGTTCTCATCTTGTTATGCCT
CTCTCAGCCCCTTTCTTATTATGACGTCTAACAAAAAAAGTCA
AGAACCGAATCATGTGTGTAAGAGCAAGTGACCAACAACAGC
TTTCTATTAGCACTCAGGATTCTAGGAAAATGAAAGACTGACC
ACTTGCTCTGTTTTTCCTCATGTTTTTGCAGTGTGACTACGAAC
TGTGCTGTTTAAATAATGGAAAATATTCTAATTAAAATATGTAT
TGATATTAATCTGTAATATAGTGTCCTTTCATAATCCTGGACAG
TACTCATTCTGCAAATCATTATTATTCCATTTTACAGTCATATA
TTTATAGTTTGCTTTTGAATATTTTTGGTAGCGTTTTTCTGGGTA
ACTTTATTTGACCTGTCTACTTATCAATGTACATATCAAAGTGT
ATGTTATGATTTGATTATATGCCCACATTTTGTTTAGAATTTGG
TATTGATAATGTTGCACATTGCTTTGGAAAACAGTGCCTGCCA
AATACTGGTAATCTAGCTGTAACTGCTAATTAAAATACTAACT
GTGATTGCAGTACTGTGCATTTACAAAATGACTGACAAAGAGG
AAACAAACAGCAAAGAGTTGTTTAGAGACTGTGAGTCTGTAGT
CCTAATTAATTTGGTCTTTTGAGCAGGCTTATGTGCAGAGCGAT
CATGAAAGTTGTAGCCATGCAATAATTATGAAAATTTTCACAA
ACAAGGCACTAGAAACTCAAAATATTCTGTATTATACATACAG
TTTAGACAACATAAGTTATGTAATATATTAAATATGTAATATTT
AATTCATATAGGGCAGCTTGTGTAAAGTCATGAGAAATTGGTA
TTAAACACATTCTTGCCAAGTGGATACATGTATGTGCACATAC
ATGGACATACGTGGACATTTTGTCTGTTATTTATCTACGCAGAG
TAGAATTCTAATCACCTAAAAGGTGTTTCAAATGTCTTGTGAAT
GTAAAAATATAGTACTGAATGCTCATACTCTTGCAACTTTAGTT
GCAAACCACCAACCCTCCAAGGTTTCACAATCATTTGTTTAAC
ATGCTGTCTTGTGTCCAC

>Unigene3670_All   V1R1 (transcriptome data)

GGAAATTGTAAAAAAAATCTATAGATGTGTAATGTTGTTGTTG
GTACAATTTGCTATTCATTGATGTAGAAGTGCAGTACTATAAG
GCATAGATAGATTCTATAAAACTCATAAATTTAATTGTGTTAA
GAAAACACTTTTATAGCTTCCTTTCTCATGTGATGTTATTTGTA
AGTTGCTGCCTATGTGGAGCCTCAATATTTGCTTTTTAGAGCAT
AAAGACATTAATGAGCAGAGATG

GAATCTGAGATGACCACGCGCGGCTGCTCTATCTATCTCTCA
CTGTATTGGGTATCCCTGGCAACAGCATTGTGATCTGGGCATTT
GTACAGCTGTCTTACTTTGAGCGTCAACTCCTACCAGCTGATGC
CATTGTGTTGCACCTGGCTTTTGCTAACTTGATGGTGGTGGGGG
TGCGCTGTCTTCTAGAAAGTCTTGCCACTTTCAAGGTATGTAAT
GTCTTTAGCAGCACTGGATGTAAGGCTGTCATATTTGTATATCG
CACAGCTCGTTCATTGTCCATTTGGCTGACATTTGTGCTCAGTG
CATATCAGTGCCTTAGCACAGCTGCTCCTGGTTCACGCTGGGC
CACTGCACGTACGGCCATGGCCAAGAACCTGGGTGGCATTTTT
TTGCTGCTGTGGCTCCTCAACACATCCATGAGTTCATCAGCTGT
CCTCTACTCTCTGAGTTCAAGTAACAATTCAAGTCTTATGAAAC
ATAATATAAATGTGCAGTTCTGTTATGTGCGCTTTCCTTCAAAG
CTTTCAGTGGATGCTAACGGAGCGGTGCAAGTGGGTAGAGATC
TGGTGCCTATGATTCTCATGACTACAGCAAGTGTCATTATTTTG
GTTTTCCTGTACCATCACAGTCACCAAATCAAAAATATCCGTG
GCAACACCAACAGCAGGGGTGGAGGCCCCTCGGCAGAGCAGA
GGGCAGCTATCACTGTGGTAACTTTGGTGATGCTGTATGTGAC
ATTCTATGGTGTGGACAATGGCTTGTGGATGTATACACTCTTTG
TTAAGGAAGCTATGAGCTCCTCAGTGGTGTCAGACTTGCGCAT
ATTCTTCTCATCACTCTATGCTGCCATTAGCCCCTTTGTCATCA
TTGTCTCTAACAAGAAAGTTAACAGACTTCTGAGATGTCAGCT
CGGAGAGAAGGCCCTGCAAAGCACAAAATCAGACGGTCATTC
TGTATGAAGTGCCCTATTCTAAAAATCTCCAGAAAAAAATATT
TTCCCCGATGGAACCTTTTTTTAGACATTGTGGTTCCTATATTTT
AAAACAGTATCCTTACCTGCCATTCAGCTGTATCATTTTTTTTT
TTACCACGACATGATGGAAAATTTATCAAAACCCGGTCCACAA
TAATAAAATTTATTTTTAAAATCGCAAGTAACCTAATTTATACA
GCTATCATCACAATTTATTGGAGTAAGTTACAAAATGAAAAAA
GGCTTGTTTTGAATGTTATAACACATAAATGTTATTAACACATA
ATAAATGGT
