# Supplementary material for: Identification of a uniquely expanded V1R (ORA) gene family in the Japanese grenadier anchovy (Coilia nasus)
Source: Mar Biol. 2016 May 2;163:126. doi: 10.1007/s00227-016-2896-9 (PMC4853444; doi:10.1007/s00227-016-2896-9)
Supplement: Supplementary file 4 — Supplementary Table S4. Primers used for real-time quantitative PCR of the V1R genes of the anadromous and non-anadromous forms of Coilia nasus (PDF 354 kb) [file 227_2016_2896_MOESM4_ESM.pdf]

Electronic Supplementary Material

Identification of a uniquely expanded V1R (ORA) gene family in the Japanese grenadier anchovy (*Coilia nasus*)

Guoli Zhu[a], Wenqiao Tang[a *], Liangjiang Wang[b], Cong Wang[a], Xiaomei Wang[a]

[a] College of Fisheries and Life Science, Shanghai Ocean University, Shanghai, China

[b] Department of Genetics and Biochemistry, Clemson University, Clemson, South Carolina, United States of America

* Corresponding author: College of Fisheries and Life Science, Shanghai Ocean University, Shanghai, China; phone: + 86-21-61900425; Email: wqtang@shou.edu.cn

Supplementary Table S4. Primers used for real-time quantitative PCR of the V1R genes of the anadromous and non-anadromous forms of *Coilia nasus*.

**V1R**	**Primers (5' to 3')**
**V1R1**	F: TCTGTATGAAGTGCCCTATTCTA
	R: CAGCTGAATGGCAGGTAAGGA
**V1R2**	F: TTGGGTCAGGTTGAAGACGAG
	R: TTTTAGGTGCGATGGAGAAGAAG
**V1R3-1**	F: TGTCACCCATTGTCCTCGCAGTAG
	R: GTGTGCATATGTGTTCAGCGTTGA
**V1R3-2**	F: TGATCCTGGCCTTAAACATGCTAT
	R: TGTAGGGCAATAATGTCCGGTGGT
**V1R4**	F: CTGTTAGTGAGTTACACCTGCCAGA
	R: TGAGAAAACCAGAAGCCCAAA
**V1R5**	F: CACGATAGAGGAAGGAGATGAACC
	R: TCTGGATGCTGGCGAAGA

**V1R6**	F: AACCCCAGTCCACCTCTCAC
	R: TGCCCACCACAGAAACGA
**GAPDH**	F: AGCTTGCCACCCTCTTGCT
	R: AGCCATCAACGACCCCTTC
