# Supplementary material for: Identification of a uniquely expanded V1R (ORA) gene family in the Japanese grenadier anchovy (Coilia nasus)
Source: Mar Biol. 2016 May 2;163:126. doi: 10.1007/s00227-016-2896-9 (PMC4853444; doi:10.1007/s00227-016-2896-9)
Supplement: Supplementary file 5 — Supplementary Text S5. Nucleotide sequences of V1Rs in Coilia nasus (PDF 317 kb) [file 227_2016_2896_MOESM5_ESM.pdf]

Electronic Supplementary Material

Identification of a uniquely expanded V1R (ORA) gene family in the Japanese grenadier anchovy (*Coilia nasus*)

Guoli Zhu[a], Wenqiao Tang[a *], Liangjiang Wang[b], Cong Wang[a], Xiaomei Wang[a]

[a] College of Fisheries and Life Science, Shanghai Ocean University, Shanghai, China

[b] Department of Genetics and Biochemistry, Clemson University, Clemson, South Carolina, United States of America

* Corresponding author: College of Fisheries and Life Science, Shanghai Ocean University, Shanghai, China; phone: + 86-21-61900425; Email: wqtang@shou.edu.cn

Supplementary Text S5. Nucleotide sequences of V1Rs in *Coilia nasus*.

>V1R1-Cna

AGTCGAGTGATAAGAACAAACAAACGACACAAACATGTTTAC
AACTGAGTCCCTACTTGAATGTATGGTGTCCCTTAAGTGGAGC
CATAGGGATTTTGATATTTTAATTATGTTGCAGGTAGCCAATG
TTATTAAGTAAAGAGGGGAGGGTCTGAACTTATGAGACACCCA
GTATTTCAAGTCTACACTGAAATAACTGGTCAGAATTCATCTTA
CCAGTCACAAATTGTGGATTTTATGGTTAACCATTTAATTACTA
TTTTTTTAAGAAGTGACAACAGTTTATAGAAGACTGTCTTTCTT
TATACATTTAAACATGTTTTATATGGAAATCACCATCTAAATGG
ATCAGATTTTGTGGCTTTGTTTAAGATAAACATGTTGTCCTTTG
ACGACATGTCCTGTCAGATGAATATTTACAAAAGGTTTTGAAG
TAGTTATAAAGCAGTGTAGTAATGGATCACATTAGAATGATTT
TAACATACTTAATTTATATGTTATTCTACAACGACTCTTATAGA
TTGAGTTCTCACCATAGATTATAAAGCTCCTATTTGCTCTACAT
AATGGCACTGATGATAGTTTTGCCTTACATTAACATGCTGTTTA
TTCACAAAATGCTCTTTGTGAAGACATGTTTAAATTCATAAATG
TTGACATGTTTCAGCATTTTAGTCTTTAAGGAGCAGATGGGTTC
TTGAAAAAACACTTGCACTATAGAGAAGTAGCACTGTTTTGCA
TCATAAGTCTCTTTAGAGTAAATGCACCCCAACCAATTGACCA
GTCATGTCTTTGTGCATATAGTTTGTTCTAATGACACAGTCTAG
CTTAATGAAGCATCCAGCTAAACAGATTGTTGTTGCCACTTCA
CTGCACATTGGCACTCTGTAGGCTATAAAAGGACAGGCAGGCA
TTAGGTGTTTTTATTTTGAGGTGGTTTGTTTAGGTACGGAAATT
GTAAAAAAATCTATAGATGTGTAATGTTGTTGTTGGTACAATTT
GCTATTCATTGATGTAGAAGTGCAGTACTATAAGGCATAGATA
GATTCTATAAAACCCATAAATTTAATTGTGTTAAGAAAACACT
TTTATAGCTTCCTTTCTCATTTGATGTTATTTGTAAGTTGCTGCC
TATGTGGAGCCTCAATATTTGCTTTTTAGAGCATAAAGACATTA
ATGAGCAGAGATGGAATCTGAGATGACCACGCGCGGGCTGCT
CTATCTATCTCTCACTGTATTGGGTATCCCTGGCAACAGCATTG
TGATCTGGGCATTTGTACAGCTGTCTTACTTTGAGCGTCAACTC
CTACCAGCTGATGCCATTGTGTTGCACCTGGCTTTTGCTAACTT
GATGGTGGTGGGGGTGCGCTGTCTTCTAGAAAGTCTTGCCACT
TTCAAGGTATGTAATGTCTTTAGCAGCACTGGATGTAAGGCTG
TCATATTTGTATATCGCACATCTCGTTCATTGTCCATTTGGCTG
ACATTTGTGCTCAGTGCATATCAGTGCCTTAGCACAGCTGCTCC
TGGTTCACGCTGGGCCACTGCACGTACGGCCATGGCCAAGAAC
CTGGGTGGCATTTTTTTGCTGCTGTGGCTCCTCAACACATCCAT
GAGTTCATCAGCTGTCCTCTACTCTCTGGGTTCAAGTAATAATT
CAAGTCTTATGAAACATAATATAAATGTGCAGTTCTGTTATGT

GCGCTTTCCTTCAAAGCTTTCAGTGGATGCTAACGGAGCGGTG
CAAGTGGGTAGAGATCTGGTGCCTATGATTCTCATGACTACAG
CAAGTGTCATTATTTTGGTTTTCCTGTACCATCACAGTCACCAA
ATCAAAAATATCCGTGGCAACACCAACAGCAGGGGTGGAGGC
CCCTCGGCAGAGCAGAGGGCAGCTATCACTGTGGTAACTTTGG
TGATGCTGTATGTGACATTCTATGGTGTGGACAATGGCTTGTG
GATGTATACACTCTCTGTTAAGGAAGCTATGAGCTCCTCAGTG
GTGTCAGACTTGCGCATATTCTTCTCATCACTCTATGCTGCCAT
TAGCCCCTTTGTCATCATTGTCTCTAACAAGAAAGTTAACAGA
CTTCTGAGATGTCAGCTCGGAGAGAAGGCCCTGCAAAGCACA
AAATCAGACGGTCATTCTGTATAAAGTCCCCTATTCTAAAAAT
CTCCAGAAAAAAATATTTCCCTGATGGAACCTTTTTTAGACA
TTGTGGTTCCTATATTTTAAAACAGTATCCTTACCTGCCATTCA
GCTGTATCATTTTTTTTTTACCACGACATGATGGAAAATTTATC
AAAACCCGGTCCACAATAATAAAATTTATTTTTAAAATCGCAA
GTAACCTAATTTATACAGCTATCATCACAATTTATTGGAGTAA
GTTACAAAATGAAAAAAGGCTTGTTTTGAATGTTATAACACAT
AAATGTTATTAACACATAATAAATGGT

>V1R2-Cna
GGGGCAGGGCAGGGCAGGGCAGGGAGAGAATTCAGCATCTTG
CACTGAAGAGGCTGTTGGATGGATGGGTGGGATTTCCCGCAAT
GCAGAGGGCTTTGCAGGTGAGGCGGGTGTGGTATATGTCCTGC
AGGGAGGGGAGAGAGACACCCACGTAAGAAATGCAATAGTCTT
GTTTGTGATCCTTGGAAACATACAATAAGCTTGTTATTTTGCCAG
AAGCACCATCATCACACTCTGGCTAATGAGATAAACATCATTATG
ATGAGATGTTTACATGGTGATGAATCTTCCCTGCTGTCTCTTAGT
CTCCAGTCTATTCCATTTTTACATTTTATAGAAAGTTTCCCCGTAA
CCATGCACAGACTTTTTGAGTGCAACATTAATGCATTCAAACAT
CTGTCATTAGTAACAAACAATTAAAATGTGATTAATCATGATTAC
CTATACAGTTAATGACTCTATCTCCCTGCTGTGTTTGTGTCAATC
ACCATTTGTTTTTTTTAACATTTGTTCCTAAACTCTCTATTCCCCA
TAGTGTCTGACAGCCTATATAATGACTCTGGTTATGTCAGTGTAC
AATCGGCAGGAGTCAAATGCATGCAGGATGAACATTTTCTGTAT
TACAGAAAACATTTACAGATGATAGTAATTCTAAGTTACTTATCT
CAATTTCCTTACATTTCTGTCTTTCTAATCTTAGTTGCATAATTCC
TAGGTCAGCGTGCTCTGTTGCAATGGACCTGTGCCTGTCTATTA
AGGGTGTCTCCTTCCTCTTGCAGACTGGTTTGGGCATTTTTGGA
AATGTTTTGGTGCTGTTGGCTTACATTCAAATTGTATGCCTAGAG

CCGCATTTGCTACCTGTAGACATAATCCTGTGCCATCTGGCCTTC
ACTAACCTGATGTTACTGCTGACTCGATGTGTACCTCAGACCAT
GACTGTGTTTGGCTTACGTAATCTCCTGAATGATGCAGGGTGCA
AGGTGGTTATCTACTCCTACCGTATTTCCCGTGCCCTCTCCGTCT
GTATTACCTGCATGCTCAGTGTCTTCCAGGCTCTTATGCTGGCCC
CAGCTAAGCCTTTTTGGGTCAGGTTGAAGACGAGACTACCTAG
CCTTGTCATCCCAACATTTGCTGCCCTCTGGTTCATCAACATGGC
TGTGTGCATTGCTGCGCCCTTCTTCTCCATCGCACCTAAAAATG
GCACGGTGCCAGCTTTCACACTCAACCTAGGATTTTGTCATGTG
GACTTTCGTGATAACCTATCATATGTTATCAATGGGGTAGCTGTA
TCAACACGTGACTTCATCTTTGTAGGGTTTATGCTGGGCTCCAG
TGGGTACATACTGGTGGTGCTCCATCAGCATGCCCAGAAGGCAC
ACAGTATACGACGAAGTCAGGCTGGCGCAGCCATGGAAACACG
TGCAGCAAATACAGTGGTCACATTAGTTACGCTGTATGCAGTATT
CTTTGGCATAGACAATGTTATATGGATCTACATGCTGACGGTGGA
CCAAGTACCCCCCCTGGTGGCAGACATGCGGGTGTGGTTCTCAT
CTTGTTATGCCTCTCTCAGCCCCTTTCTTATTATGACGTCTAACA
AAAAAGTCAAGAACCGAATCATGTGTGTAAGAGCAAGTGACCA
ACAACAGCTTTCTATTAGCACTCAGGATTCTAGGAAAATGAAAG
ACTGACCACTTGCTCTGTTTTTCCTCATGTTTTTGCAGTGTGACT
ACGAACTGTGCTGTTTAAATAATGGAAAATATTCTAATTAAAATA
TGTATTGATATTAATCTGTAATATAGTGTCCTTTCATAATCCTGGA
CAGTACTCATTCTGCAAATCATTATTATTCCATTTTACAGTCATAT
ATTTACAGTTTGCTTTTGAGTATTTTTGGTAGCGTTTTTCTGGGT
AACTTTATTTGACCTGTCTACTTATCAATGTACATATCAAAGTGTA
TGTTATGATTTGATTATATGCCCACATTTTGTTTAGAATTTGGTAT
TGATAATGTTGCACATTGCTTTGGAAAACAGTGCCTGCCAAATA
CTGGTAATCTAGCTGTAACTGCTAATTAAAATACTAACTGTGATT
GCAGTACTGTGCATTTACAAAATGACTGACAAAGAGGAAACAA
ACAGCAAAGAGTTGTTTAGAGACTGTGAGTCTGTAGTCCTAATT
AATTTGGTCTTTTGAGCAGGCTTATGTGCAGAGCGATCATGAAA
GTTGTAGCCATGCAATAATTATGAAAATTTTCACAAACAAGGCA
CTAGAAACTCAAAATATTCTGTATTATACATACAGTTTAGACAAC
ATAAGTTATGTAATATATTAAATATGTAATATTTAATTCATATAGGG
CAGCTTGTGTAAAGTCATGAGAAATTGGTATTAAACACATTCTT
GCCAAGTGGATACATGTATGTGCACATACATGGACATACGTGGA
CATTTTGTCTGTTATTTATCTACGCAGAGTAGAATTCTAATCACCT
AAAAGGTGTTTCAAATGTCTTGTGAATGTAAAAATATAGTACTG
AATGCTCATACTCTTGCAACTTTAGTTGCAAACCACCAACCCTC
CAAGGTTTCACAATCATTTGTTTAACATGCTGTCTTGTGTCCAC

>V1R3-Cna

TGTGGGCGTAGAGTGTCAGCAGCGAATATGTAAGCATGCCTTGG
GTCTGAATAGGTCTGAGTGTGTGTGAGTGTAATTAGGTATATGG
AAGTGAGTGGATATTTTTTCACCCTCCTGATGACGGGCACCTCA
ACGTGGTTACCATGGAGATTGGTGACATCCTTTTCCAACGAGAG
GTTGGACCACTTCATGTCAGAAGAGCAGCCTACACACATTAGTC
ATAATTTCCATGGAAGTTGGTATATAGAAGCCAATCACAGGAAA
AGACTGAATGAGTTGGAGTTTCTGTGGGCCTGAAAAGGGATTA
AATGTATTGTTTACACTGAGGTTGCACATTAGTAAAGTTGTGAAT
GGAATGGATTCTCAGACAGAAGCATCTGTGTGGAGTAAGGCGG
TCGTGGATGAAATGCACACGACCACACAGCCCGTTGGTATGGG
CCTTCGGATCCGGGTGTCGGACGTCCAAACAGTGTTTTACATCT
TCCTGGTCATGCTGGGGATCCTGGGAAATGCGACAACCATTGGC
GTGATTGGCGAGGGCATTGTGAGGGACCAAGGTGGGGGACGC
AGCTCCGACATGATCCTGGTGAACATGGCCTTCTCCAACCTCAT
GGTGTCTGTCACCAGAAACACCCTGCTGGTCATATCTGACCTGG
GAGTTGAGGTAGGTGCAATGGGCGAAGGTGTATGGGGAAAAA
GGAGGTCAAAGTGTTTTCTCTCAGGGCCAACCCATAATAAGACA
TAAGACAAGATTATGTTTTCGGCTTATCTACCTTGGTGATCTGGG
CCAGGGCCATTGGGTCCAACCGTGGCTTGCGGGCCCTTTGCTG
ATGCCAGTAATACTATTAGTAATAGCATGGCACATTTTGTATAGG
GCCTGAAATTTCTGGCTACAACCCTAATCTAGGCAGCCAAAGAG
AAGTCTTAGAGAGAGAGTGGTACAGGAATGCCCCATTTCCTCTC
AGTGCTGACACTGCTGTATTATAATGGCTTGCTATAAATTCATGA
ATTGATTGCCTTCATGATTGAAGATCAGAGAAATTGACAAGAGC
AGGAATCGCATTAATCTTAAATCTGGGAGGTCTTTTCAGTGAGA
GTTCCACCTTAACTAACTGCTATTCCGTTTGAGTGTGACAGTAG
GGAAAGCTGTTTGGAACAAATAAGTGTTTCCTTTGTTTCATTATT
TTCTTAAGAACTAAAGATTGAGATGAAGTTTTGTCATGACCTAC
TGAGGCATTCCTGGCACCATAGATAATTGCAGGTGAGAAGCCTG
ACCAACACCCTCTTTATTGTTCAACAGCTTAAATACTTAAAAGT
TGAGACTTGCTTTCTGAGTGTGATATTTTGTGACATATTTCAGAT
GTCTCTTGAATGTCATGTCATTTAATCCCACTTAATGAGCACACT
ATTTTGCAGTTACATGTCTACGGTGTATACATTGTGAATGCTTAT
GTCATTACGGGTGCAAATCTCTTTCTAATGTGTTTATTCTATTGA
CTTTGCTGTTCTGTCTGAGGACAGCTGTACTCATCTAGGGAATG
GTGCCAGTTTCTCATGGGCCTTTGGGTGTGGCTGCGGTCCGTCA
ACGTGTGGTCCACCTTCTCCTCAGTGCCTTCCACTTCCAGACTC
TCCGTCGCATCGCTCCCATGTCCGTGACCAGAGGACCTTCCAAA
TTCTTCTTCCTCATCTTCGGCCTGATTTGGTTCCTCAATCTGCTCT
ACTCCATCCCTGCGTTTGTCTTCTCAACCAGCGGGGACAGGAA

CTCCACAGAGGTCAGTGAGTCACCGCACCACAGTAAACAGGCA
GACATCACATTGTACAAGGCAGGGAGATTAAGGACCAGTTAAT
GCACAGGACACTGGCCGTTACTGTAGTTACTGGTCTTCATAATA
CTGGCAACTTTACGAGACCTGGACATTTGTGATGCTGGTGTTAT
CTGCCTCTCAGAATTCATACTAAACAGAAGAACCATTACAGCAA
AGACAGAAGGTAATTTACTCAGCTGTTGTAAATAGCCTGAAAA
AGAATATTACTTTTTTGACCGGGACAAAATTTATAGATAGGATAA
AGAAATGAAAGTAAATATTTAACTTTTACTATTATTGAAATGCCC
ACCTGTAGACCCTGATGTTGGTGAGCAGCACCACACGCCCCCT
GCTGGGCTGCGTGTGGAACTTCCCCACTGTCTACAATGGGCTG
GCCTACGCCACCACGTCCATGGTGATCCACGAGAGCCTGCCCAT
CGTGTTGATGAGCATCACCAACCTGGGTTCGCTGCTGACACTCT
ACGCCCACAGCCGCTCCCTGCTGCACTCTCAGAAGAACCTCGA
GGTGCCCGTCATCAGGAGGGTGCCAGCTGAGAGACGCGCTGCC
AAGGTGAGTCACACTACCAGTGCTGCTAAGGTGAGACACTACC
AGTGCTGCTAAGGTGAGTCACACTACCAGTGCTGCTAAGGTGA
GTCACACTACCAGTGCTGCCAAAGTGAGTCACACTACCAGTGC
TGCTAAGGTGAGTCACTACCAGTGCTGCTAAGGTGAGTCACAC
TACCAGTGCTGCTAAGGTGAGTCACTACCAGTGCTGATGGTGA
GTCACTATCAGTGCTGCTAGGTGATATTTTAGGGTATCATTTCTT
CTACTGTTCTCAACCCATTTTACATTACTGTACTTATTTTAAGACT
GTGGCGTGCACCACAGTGTAATGTATATTTGAATAAACATGCTTA
TTGCGTGTGTACATGGTATCATTAAAACAAACCGGTATGCACATT
TCTACCTAAAATCACGGTTGTTGACACGTCAATGGTAAAATGCA
TACAGTACCACAAATAAGGTGGAAATTGTATCTCTCTCTCTGTCT
CTGTCTGTCTTTCTGTCTCTGTCTGTCTGTCTCTGTCTCTCTGTC
TCTCTGTCTCTCTCTCTGTCTCTGTCTCTCACGTGATTCTGGCTC
TAATCATGCTCTTCATCTCCTCCTGGGGCACCAGTATAATCTCGG
TCAACTACTTCAACTATAATCGCGGCACATCTACGGAGTTTCTGT
TGGTCATCGCTCGCTTCGCCAACATCACCTTCATTGCGCTGTCA
CCCATTGTCCTCGCAGTAGGACACGGACGCCTGCGAGCTGTTCT
GAAGTCCCTGCTCACTCACTGACAGCTGGACATGTCTGAAGGC
CTGAAGGGCTGTGGCACTGCATATAGTCAGTGTATTCGTTACCAT
AAATGCAGATAAGCTCACATCTCAAAACCACCAAAAACTGTCC
TTTGGAAACCTTTGAGTGGAGATCTTGAGCTGTTTTCAACGCTG
AACACATATGCACACACGCACGCACGCACGCACACACATACAC
ACACACTGAAATTCAAAATTCATTGTGGACTCTCACTGTTCCAG
AGTAAAGTGGCCTTTTCATCACAATAAGATCTAAATTTTCTCCAG
GTCTGAATATCATCAGAGATTTGTCTTTATTTCTGATCTCTTTCTG
TTGTATTCTAAATTGCCTAAATATATCAGATTTCAAATGAGCCTG
CGGTGTTATTATTCTGAGTCTTTATTCCCCAACTATGGTCTTCTCT
TTCACATCCATACAAACACAAAGACACACAACTGTGTTTACAGT
ATATTATCAGCAATGCTGCGCATTTCAATCCTCACATTTTTAAAC

TTTTTACTTTTTTTGTCAATTTATGTTAATGTTAATCTTAATCAAC
TTAATGTTAGTCAACACTTCATTTGCTTTCTACTCATGAAGCCCA
ATAAGCCCCTCCTCTTCCATAAAAGCAGGAACACTACTATACCT
ACAACATGTTTGACTGGGCAGTATGACAACTTTGAAGGCATTTT
TCTCTGTTTCAATGTTCGTGTAGTTACTGCACTTTGCCTCTGTAG
GGCAATAATGCCTCTGTAGGGCAATAATGTCCGGTGGTTTTGTT
CAGTTAATAAGAAGAGACCTGAGTACACCTCTGATATGCCTGTG
TCCGACCGCTAGCACAAAAGGGGAGAAGGCGATGAACGCGGT
GTTGGCGAAGCGAACAATGACCAGCATATACTCCGTGGATGGA
CCACGGTTGTAGTTAAAGTAGTTGATAGAGATCATACTGGTGCC
CCAGGAGGAGATGAAGAGCA

>V1R4-Cna

CTGACTGTTAGTGAGTTACACCTGCCAGATATGGCTAACAGAGA
CTCAAACAAGCCCAGCTGTTGGAGAGATGTCTGAGGTGCTCAC
GGTAGATGCCATTTTGTTTGGGCTTCTGGTTTTCTCAGGAATCGT
AGGGAACATTCTGGTCATATATGTGGTAAGTCATAGGGAACATT
CTAGTCATATATGTCGTAAGTCAGACAGTCTGACAACAGCTTCA
TTCTGAGAAACATACTAAGTCTCCTTAAGAGTATCAACAGTTCA
GTTACACAAAATTGTATGCGTTGCTAAATTCATCTATTTTCATTG
ACTCAGTTATTGATGAATTTTGAATAATTTTAGTTTATAATACTGG
CACCTCTCTGATTCCATCATCTAGCTTCCACCATCTCACACCCCT
CTCCCATTCTTTGAGTCTTGTATCATGGGCTGGGTTTGAACCTCA
TGCACTGACTGTAATTTACTCTGCCTGGGGATGGTCAAAGGACC
AAGAAGCTTGTAGAGTGAAGCAACTGTTTGCACAAATTTGAGT
GTGCGGATTGTCCTGTCTGTCTCAGGTCTTCGGGTCGGCCACGG
AGAATTCCTTCCGCCACCTCCCTCCCTCCGACGCCATCCTAGTC
AACCTGTCGCTGGCCAACCTGCTGACGTCGCTGTTCCGCACCG
TGCCCATCTTCATCTCGGACCTGGGCCTGGAGGTGAGCCTGGC
GCCGGGCTGGTGCCGGCTCTTCATGCTGCTGTGGGTGTGGTGG
CGTGCGGTGGGCTGCTGGGTGACGCTGGCGCTGAGCGCCTTCC
ACTGCGCCAAGCTGCAGCGGCAGCGCATGGTGGTGGGGCCACT
GGCGCAGCGGCGGGAGCGGCAGCAGGTGTGGCTGGCCCTGGC
GCTGGTGTGGGGCGCCAACCTGGCCTTCTCGCTGCCCGCGCTG
GTCTACACCACGCACGTGCATGGCAACGCCACCGTGGAGCTCA
TGGTGATCAGCTGTACCACGCGGCCCCTGCTGGGCTGCGTCTG
GGAGTTCCCGACTGAGGAGCAGGGCTCGGCCTTCGCCTCGGCC
TCGCTGGCGCTCAACGAGGTGGTGCCGCTCGTGCTCATGATGG
GCACCAACCTGGCCACCCTGCACACGCTGGCGCGCCACATCCG

CTCCGTCACGGCCGCCCAGCAGCCGCAGCAGCAGGCGGAGCT
GGGAAGGCACGTGGCCAGCGAGCGCAAGGCGGGCCACGTCAT
CATGGCGCTGGTGTCGCTGTTCGTGGTGTGCTGGGCGCTGCAG
GTGGCCGCGGTGACCTACTACAACCACAACGGGGGAAACCAC
GCCGAGGGCCTGCTGACCGTCTCGCACTTCTCCGCCTCGCTGTT
CGTGGGCTTCAGCCCCATGGTGGTGGCCCTGGGCCACGGCAAG
CTGCGGCGCCGGATCACAGCCATGGTGCTGAGCTGCCTGCACA
GGGCCAAGTGCGGCGCGCAGGGCGGAGCAGAGCAGGCGCTCG
TGTCTGACATGAGTGTAAGTAAACAAACTGAGCTGAGCAAAGG
CCAGAGAGACAAACGCATCATAAAGGTGGAAGCACGAGGTAG
AACCTAGCTACACATGCAAGTGTCTGATAGTGGATTTGTGTGTA
AGTGCATGTATGTGTGTGTGTGACTTTGTGTGAGTGTGTGTGTG
AGTGTATGCATGTGTGTGCATCTGTGCATGTGTGTGCGTGTGTG
AGTGTATTACCGGTGTGGCGTCTGCCGCCCATCTCCTCGCACAG
CTGGCTGAAGGGGTTGGGGGGCAGGGGGCCCGGCTGCTCCTC
AGTCGGGAACTCCCA

>V1R5-Cna

GTACATTGCAACCCAACCAGCTGAAGAATGGATGCAGAGGGGT
GGGTCGAGTCTTTTGCCAGGGGTACTATGTGCTTATTGGGTATAG
TAGGCAACAACTGGCTAGCTTTTAGTTCATTCCCAAGATCCAAG
TCACAGCTGAAGACTAATGACGCTCTATTCCTCAATCTGGCTGT
GTCCAACCTCATCACTAACTACATGGTAGATCTGCCCGACACCA
TGGCAGATTTTGCGGGCCGCTGGTTCATGGGCCTGACCTACTGT
CGAATCTTCCGCTTCTGTGCCGACCTGTCAGAGACCAGCAGCAT
CTTCTCCACCCTCTTCATCAGCGTGTTCTGGTACCAGAAGCTGG
TGGGCTCCCTGAAGCGTGGAGGGGCCCCGGTCCGCTTGGACAA
CCTGCGTCTAGTCGCTGCCCTTCTGGGAGGAAGCTGGATGGTG
GCTATCGTCTTCAGCATCCCGCATCTCATCTATGTCACGATAGAG
GAAGGAGATGAACCTGACTGTGTGGATGACTTTCCTTCTCCCAC
TGCCCATCAGATCTACGAGATCCTGTACTTGAGCTTGGCAAATG
CCGTTCCGATCACTGGCATCGTCTTCGCCAGCATCCAGATCGTG
GTCACCCTTCTAAAAAATCAGCAACGCATCAGGGCAACAGGAG
CTGGAACAGGTGAGCCTTCACCAGACAAGCCTCAGGACACTGC
AGATACAACTAGTCCAGGTCAAGCTCAGGCACAGCAAGGCTCT
AATTCTCCATCAGCTCAAGGTCCACCCAGCAGTGCACCAGGGG
CTGCTGTCCCGCAGCAAGGGCAGGCAGCACCCAAAGCACAGG
CGAAGGGCAGCCCTGGTGCTGGTGGCCTGGTGCGGGCTGCCAA
GAGTGTGGTGGCTGTGGCCTCCGTGTTTCTAGTGTGCTGGGTGA

CCCATCTGCTCCTCCGCATCAGCAGCAACGTGAAGACGTCCAA
GGTTGTGACGGAGGTGGCCAGCTACATCGCAGCGTCCTACACC
AGCATCATCCCCTACATCTTTCTGCACGGTGTGAAGAAACTCAC
CTGCAACTGTAGACGGTGAGCTGGAAATGGTCAGAGCCTGTGG
ACCACTGACATTCAGCTTGTAGAACAGTTATCATTATACAACAC
TGAGCAGAATTTAAGGTTTTATGTAATATATACACATGTACAGTC
ATATATACAGTTGTGTTCATAAGTTTACATACCCTGGCAGAATTT
TTTCTTAACCATTTTTTTAGAGAATCGTTTGGGTAGTTTCTGTTC
ACAGATGTTTGACAATAAATTACTTCAGCAAACCACTAACCATG
AGTGAAAAAAAAGTTTGTGTCATCATTCATATCCTCTGAAAAAT
GGTTAAGAAATCATCAATTCTGCAAGGGTATGTAAACTTATGAG
CACAACTGTACACATGAAATATTTATCATGAAATGTCTGATGCCT
AAGCAACTACTGGTATAGCTGACCCATTAAAGACTAAATGTATT
TTTATGTGAACGTTATTTCATATAGGCCTACAATAAAATCATGTG
CCTAAATTTCAGTTTAATGCACAGATTGGTTGCAATCACGCCAG
TATGGGGAGAAGCTCACTTCTGCCAACAGTGCACTGCAAGATG
GGTCCCAGCATCGCTTGAGTTTTACGGTGTTTGATATGCTAAATA
AAATACTGTTCATTTTAGATTCGACATCAAAGAGTTTTATTCTGG
CTAACACCAGTGTCAGTAGAGCTTCTGTGAATAGAGACAATTGG
CTCAGAAATGGCATAATTGCAAACATATTTGAGTAATTGTTCCCC
TGTTCATTGCTCAATTAGGATAAAACGGATTCAGGGGAAGATTT
TTTCCCAGGAC ACGCTGATGAAGAGGGTGGAGAAGAT

>V1R6-Cna

ACTTGTAGAGGTGGTTGAACAGTGGGCAGGCTGCACTTATATCA
TGCTGTAACGCATTGGGGAGGGCAAAGCTCACAGGCACCAATT
AAAGTGGTCCCATCACAAGCTCGTAATGAACCAGTGATCACCA
GAAAGGGTGTGCAGGTCTCTACAGATGAGGATGGTGACATCAA
AGACGTTAGGCCAGTGAAGCAGAAGGACACAGAGAATCCTTAA
TGAACCCCAGTCCACCTCTCACTCTGTTTGACTGCACCCCTTGC
CTTATCCGTGTAGTGACAGACTCTACACTCAGGTGTGGGATGGC
GTTGCAGGTGTACCTGTTGATCCTGAGAGGTCTCGTTTCTGTGG
TGGGCATTATTGGCAACGTGGTGTTGATCCAGTCCATCCTGAGG
CTGGCTCGTTTTAAGACGTTCGAAATTTTTCTGCTGGGGCTGGC
CTTCTCCAATGTGGAGGAGATCCTGATCGTGGACATCTACGACA
TTGTCGTGAACCAGCTGTCCTGGATGGAGATCAGCGCCTGGTG
GTGTCGCTTGCTGAAGTTCCTGACAGTCCTCGGCGAGATTGGC
AGCATCGCGTTCACCGTCATCATCAGCATCTTCCGCTACCAGAA
GCTACGTGACGCCGAGCGACGCATCAGCCAGACGATCCTCATG
GATGACGGGCGCGCCGTGTACGGCTTCTGTGGCGGCAGCGTGC
TGTTCGCGCTCGCCCTGGCAGTGCCCACCTTCGTCACCAACCTC

GACGGGCACATGGGCAACCTCACCCGCCGCACCAGCTGCCCAC
CAGACTTCTTCCAGTGCCCCCGGAGCAACTGCCCACTGTTCAA
CCACCTCTACAAGTACCTGTTCATCCTGCTGTGCAACCTGCTGC
CCTTGCTCATCGTCACCTGGACCAGCTGCCTCATCATCAGGGTC
CTGATTGGCCAGCAGAAGGCGGTACATGCCCGCCAGACCGTGC
AGCCCGGGTCGCAGGCACAGACGCGTTCCAGGCGGTTCCGGC
AGAGCACCGTGGCCATCCTGGTTGCCATGGCAGTGTTCCAGGT
GGACTGGACGCTGTACCTGGTGCTGCACCTGACCTCCAGTCCCT
ACACTTTCGCCGCCTGGTCTGAGATGGAGTTCTTCATCACCACC
ACCCACACCACCATCAGCCCCTATGTGTACGGGGTAGGAAACA
ACCTGTTCTCGTTCAAGGCCCTCAGGTGTACGGGATAGAAAAC
AAAATGCTCTCGTCACGACCCTCAGGTGTGATGTATCTGTGCGG
AATAGGAAATAGGAATAGAAGGTAAAATAGAATGCCCTATATTG
TCACTATACACATGTACAATAAGACAGAATGTGTAACTACTGTCT
GACCTGTGGGCAAGAAAACAGAGGGGTGGGGTTCTACTAGTGC
TGCTACCAGTTGTGGTGTTACGTTCAAAAGTCATTCTTAACTGT
AAAATATTTTACCTTTTTACATGTTGTTTTACATCTGTCTTTCTTT
CAAACTTCAATTTTAACAGTTCTTATTTAAATTATATAAACTATTT
AACATATTACCGGATTTATTTTGTAGTGCATCATGCTCAACACTT
TGCTGTATCTCCACTGCATTGGAAATGAAGGCTACCGTTAGTCAT
ACTTGTACTGTACATGTTACGTTACATTACATTACACTTAGCTGA
CGCTCTTATCCAGAGCGACTTACAGATATTTTACAGGGTTTCAGT
TACAGTCCCTGGAGCAGGGTGGGGTTAGATGCCTTGCTCAAGG
GCATCTCAACCATGGGTAGGGCGGGATTCGAACCGGCAACCTT
CCGATTGCAACACCAAACTCGAGA
