# Supplementary material for: Identification of a uniquely expanded V1R (ORA) gene family in the Japanese grenadier anchovy (Coilia nasus)
Source: Mar Biol. 2016 May 2;163:126. doi: 10.1007/s00227-016-2896-9 (PMC4853444; doi:10.1007/s00227-016-2896-9)
Supplement: Supplementary file 6 — Supplementary Text S6. Amino acid sequences of V1Rs in Coilia nasus (PDF 302 kb) [file 227_2016_2896_MOESM6_ESM.pdf]

Electronic Supplementary Material

Identification of a uniquely expanded V1R (ORA) gene family in the Japanese grenadier anchovy (*Coilia nasus*)

Guoli Zhu[a], Wenqiao Tang[a *], Liangjiang Wang[b], Cong Wang[a], Xiaomei Wang[a]

[a] College of Fisheries and Life Science, Shanghai Ocean University, Shanghai, China

[b] Department of Genetics and Biochemistry, Clemson University, Clemson, South Carolina, United States of America

* Corresponding author: College of Fisheries and Life Science, Shanghai Ocean University, Shanghai, China; phone: + 86-21-61900425; Email: wqtang@shou.edu.cn

Supplementary Text S6. Amino acid sequences of V1Rs in *Coilia nasus*.

>V1R1-Cna

MESEMTTRGLLYLSLTVLGIPGNSIVIWAFVQLSYFERQLLPADAIV
LHLAFANLMVVGVRCLLESLATFKVCNVFSSTGCKAVIFVYRTSR
SLSIWLTFVLSAYQCLSTAAPGSRWATARTAMAKNLGGIFLLLWLL
NTSMSSSAVLYSLGSSNNSSLMKHNINVQFCYVRFPSKLSVDANG
AVQVGRDLVPMILMTTASVIILVFLYHHSHQIKNIRGNTNSRGGGP
SAEQRAAITVVTLVMLYVTFYGVDNGLWMYTLSVKEAMSSSVVS
DLRIFFSSLYAAISPFVIIVSNKKVNRLLRCQLGEKALQSTKSDGHS
V*

>V1R2-Cna

MDLCLSIKGVSFLLQTGLGIFGNVLVLLAYIQIVCLEPHLLPVDIILC
HLAFTNLMLLLTRCVPQTMTVFGLRNLLNDAGCKVVIYSYRISRA
LSVCITCMLSVFQALMLAPAKPFWVRLKTRLPSLVIPTFAALWFIN
MAVCIAAPFFSIAPKNGTVPAFTLNLGFCHVDFRDNLSYVINGVAV
STRDFIFVGFMLGSSGYILVVLHQHAQKAHSIRRSQAGAAMETRA
ANTVVTLVTLYAVFFGIDNVIWIYMLTVDQVPPLVADMRVWFSSC
YASLSPFLIMTSNKKVKNRIMCVRASDQQQLSISTQDSRKMKD*

>V1R3-Cna

MHTTTQPVGMGLRIRVSDVQTVFYIFLVMLGILGNATTIGVIGEGI
VRDQGGGRSSDMILVNMAFSNLMVSVTRNTLLVISDLGVEGMVP
VSHGPLGVAAVRQRVVHLLLSAFHFQTLRRIAPMSVTRGPSKFFFL
IFGLIWFLNLLYSIPAFVFSTSGDRNSTETLMLVSSTTRPLLGCVWN
FPTVYNGLAYATTSMVIHESLPIVLMSITNLGSLLTLYAHSRSLLHS
QKNLEVPVIRRVPAERRAAKVVILALIMLFISSWGTSIISVNYFNYN
RGTSTEFLLVIARFANITFIALSPIVLAVGHGRLRAVLKSLLTH*

>V1R4-Cna

MSEVLTVDAILFGLLVFSGIVGNILVIYVVFGSATENSFRHLPPSDAI
LVNLSLANLLTSLFRTVPIFISDLGLEVSLAPGWCRLFMLLWVWW
RAVGCWVTLALSAFHCAKLQRQRMVVGPLAQRRERQQVWLAL

ALVWGANLAFSLPALVYTTHVHGNATVELMVISCTTRPLLGCVW
EFPTEEQGSAFASASLALNEVVPLVLMMGTNLATLHTLARHIRSVT
AAQQPQQQAELGRHVASERKAGHVIMALVSLFVVCWALQVAAVT
YYNHNGGNHAEGLLTVSHFSASLFVGFSPMVVALGHGKLRRRITA
MVLSCLHRAKCGAQGGAEQALVSDMSVSKQTELSKGQRDKRIIK
VEARGRT*

>V1R5-Cna

MDAEGWVESFARGTMCLLGIVGNNWLAFSSFPRSKSQLKTNDAL
FLNLAVSNLITNYMVDLPDTMADFAGRWFMGLTYCRIFRFCADLS
ETSSIFSTLFISVFWYQKLVGSLKRGGAPVRLDNLRLVAALLGGSW
MVAIVFSIPHLIYVTIEEGDEPDCVDDFPSPTAHQIYEILYLSLANAV
PITGIVFASIQIVVTLLKNQQRIRATGAGTGEPSPDKPQDTADTTSP
GQAQAQQGSNSPSAQGPPSSAPGAAVPQQGQAAPKAQAKGSPGA
GGLVRAAKSVVAVASVFLVCWVTHLLLRISSNVKTSKVVTEVASYI
AASYTSIIPYIFLHGVKKLTCNCRR*

>V1R6-Cna

MNPSPPLTLFDCTPCLIRVVTDSTLRCGMALQVYLLILRGLVSVVG
IIGNVVLIQSILRLARFKTFEIFLLGLAFSNVEEILIVDIYDIVVNQLS
WMEISAWWCRLLKFLTVLGEIGSIAFTVIISIFRYQKLRDAERRISQ
TILMDDGRAVYGFCGGSVLFALALAVPTFVTNLDGHMGNLTRRTS
CPPDFFQCPRSNCPLFNHLYKYLFILLCNLLPLLIVTWTSCLIIRVLI
GQQKAVHARQTVQPGSQAQTRSRRFRQSTVAILVAMAVFQVDWT
LYLVLHLTSSPYTFAAWSEMEFFITTTHTTISPYVYGVGNNLFSFKA
LRCTG*
