# Supplementary material for: Identification of a uniquely expanded V1R (ORA) gene family in the Japanese grenadier anchovy (Coilia nasus)
Source: Mar Biol. 2016 May 2;163:126. doi: 10.1007/s00227-016-2896-9 (PMC4853444; doi:10.1007/s00227-016-2896-9)
Supplement: Supplementary file 7 — Supplementary Fig. S7. Amino acid structures of V1Rs in Coilia nasus (PDF 1029 kb) [file 227_2016_2896_MOESM7_ESM.pdf]

Electronic Supplementary Material

Identification of a uniquely expanded V1R (ORA) gene family in the Japanese grenadier anchovy (*Coilia nasus*)

Guoli Zhu[a], Wenqiao Tang [a *], Liangjiang Wang [b], Cong Wang[a], Xiaomei Wang[a]

[a] College of Fisheries and Life Science, Shanghai Ocean University, Shanghai, China
[b] Department of Genetics and Biochemistry, Clemson University, Clemson, South Carolina, United States of America
* Corresponding author: College of Fisheries and Life Science, Shanghai Ocean University, Shanghai, China; phone: + 86-21-61900425; Email: wqtang@shou.edu.cn

Supplementary Fig. S7. Amino acid structures of V1Rs in *Coilia nasus*.

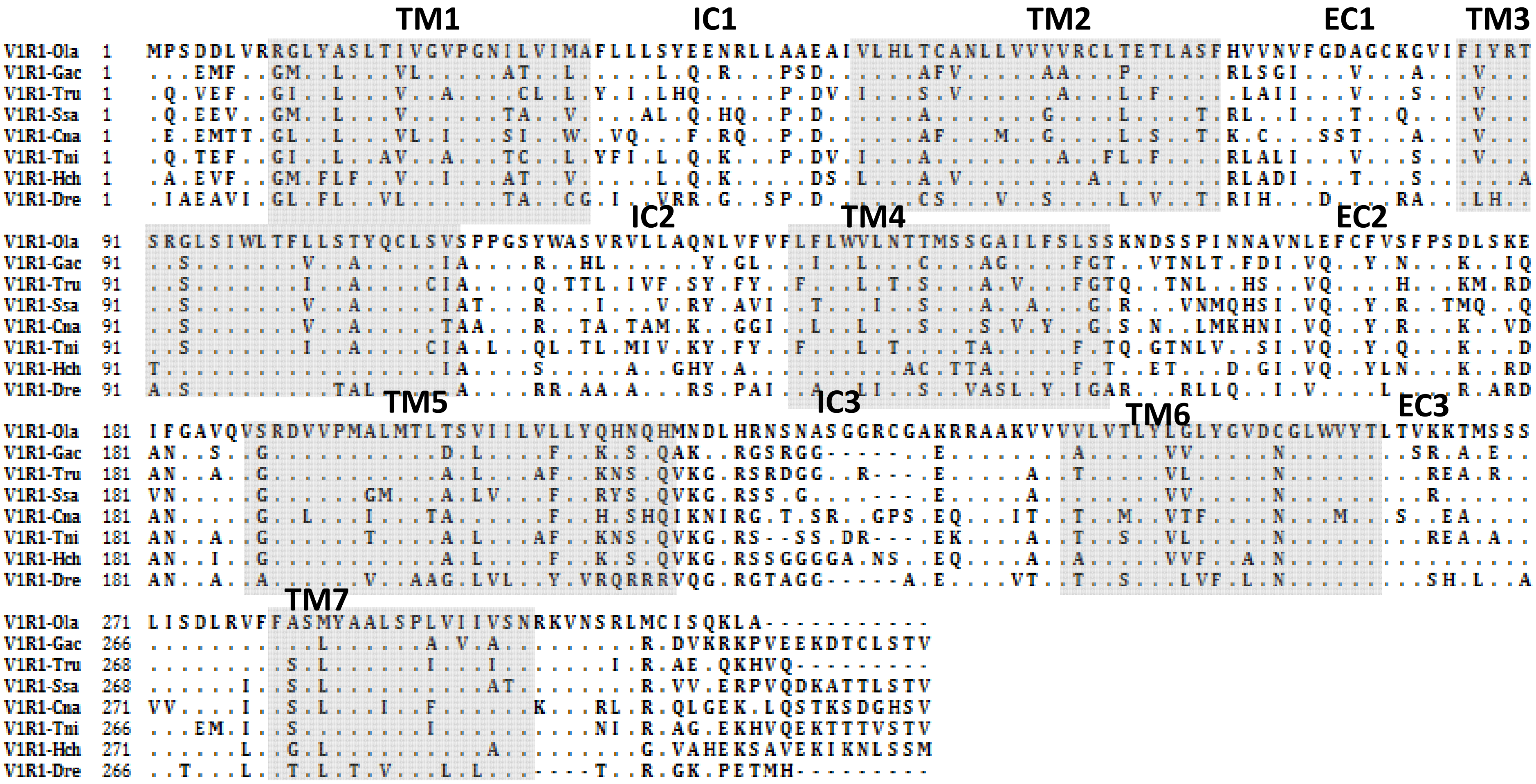

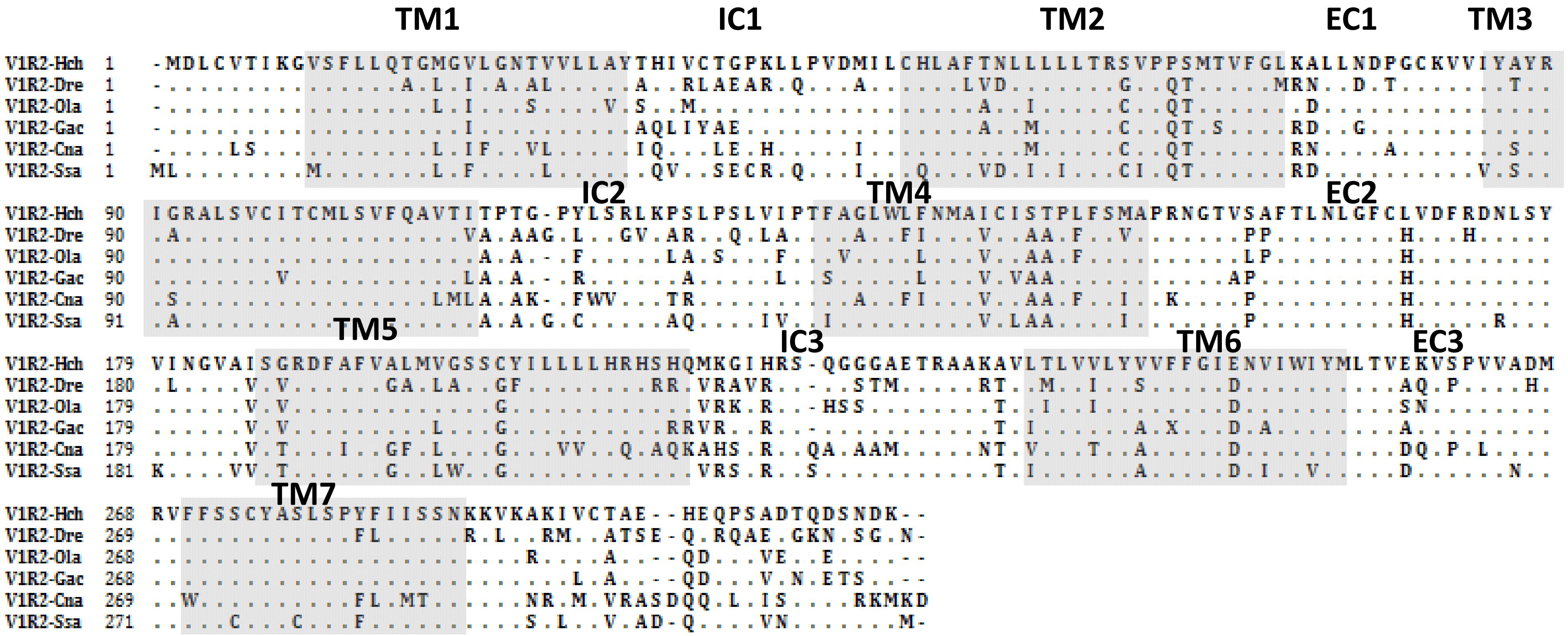

TM1
IC1
TM2
EC1
TM3
IC2
TM4
EC2
TM5
IC3
TM6
EC3
TM7
V1R2-Hch
V1R2-Dre
V1R2-Ola
V1R2-Gac
V1R2-Cna
V1R2-Ssa

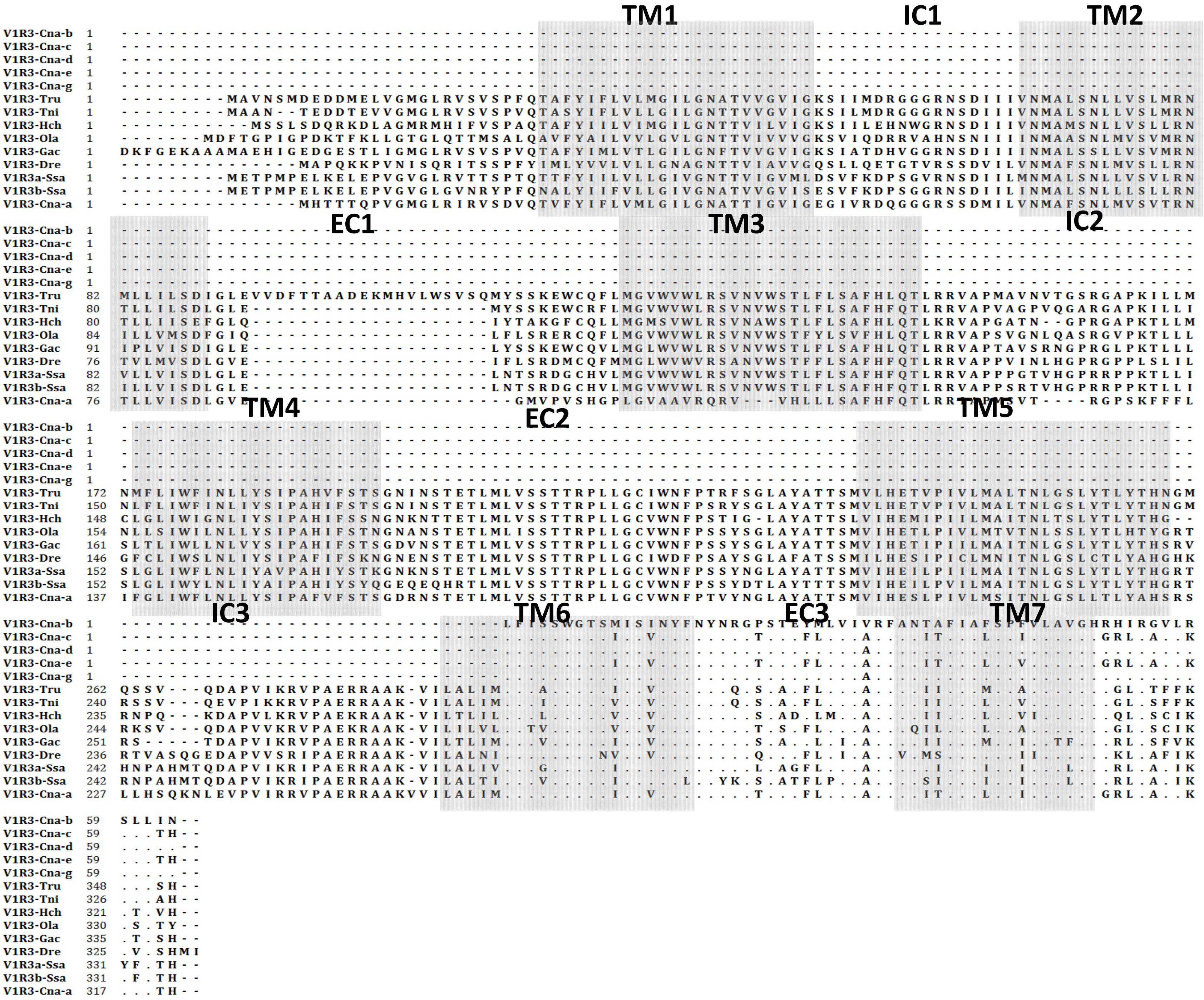

TM1
IC1
TM2
EC1
TM3
IC2
TM4
EC2
TM5
IC3
TM6
EC3
TM7

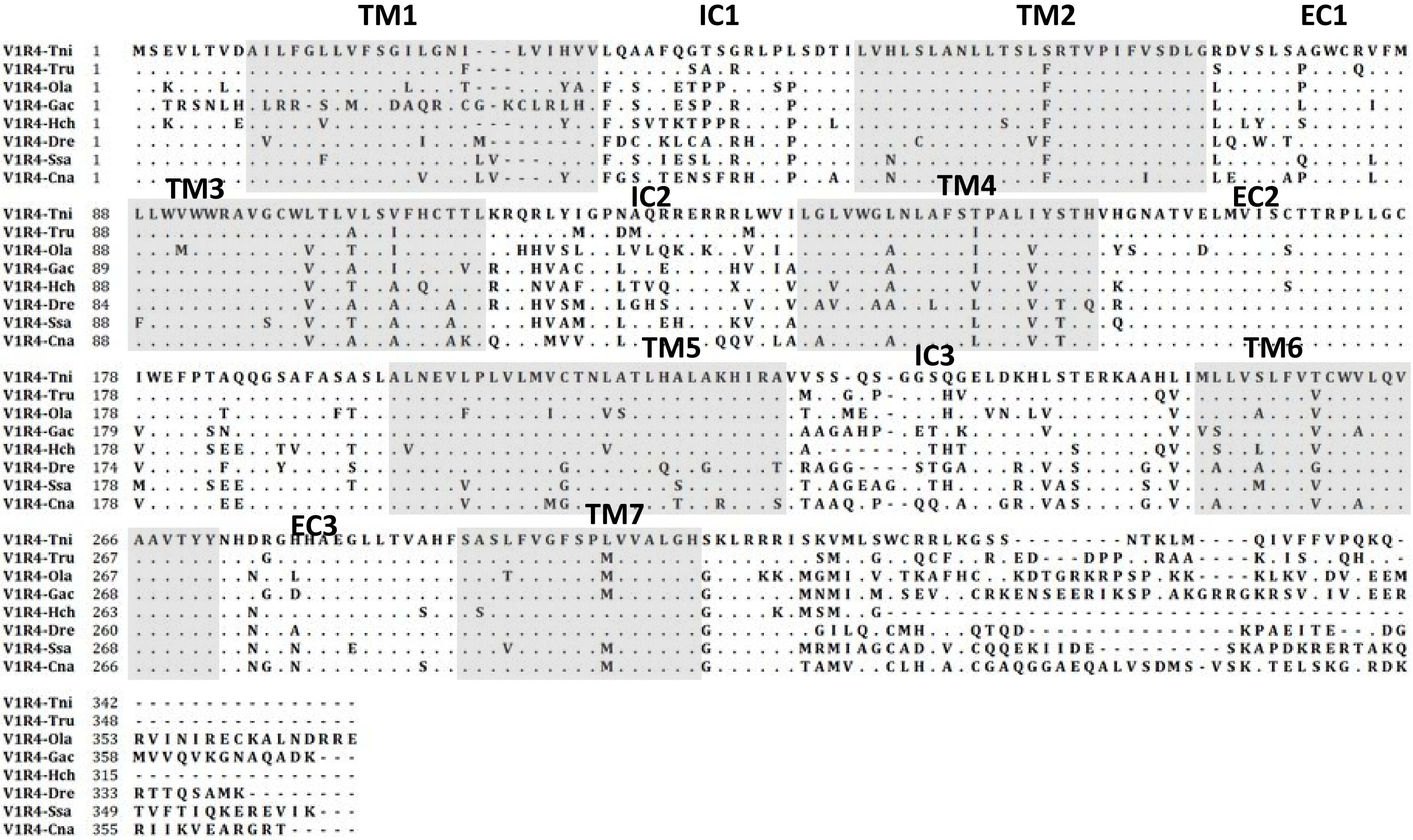
TM1
IC1
TM2
EC1
TM3
IC2
TM4
EC2
TM5
IC3
TM6
EC3
TM7
V1R4-Tni
V1R4-Tru
V1R4-Ola
V1R4-Gac
V1R4-Hch
V1R4-Dre
V1R4-Ssa
V1R4-Cna

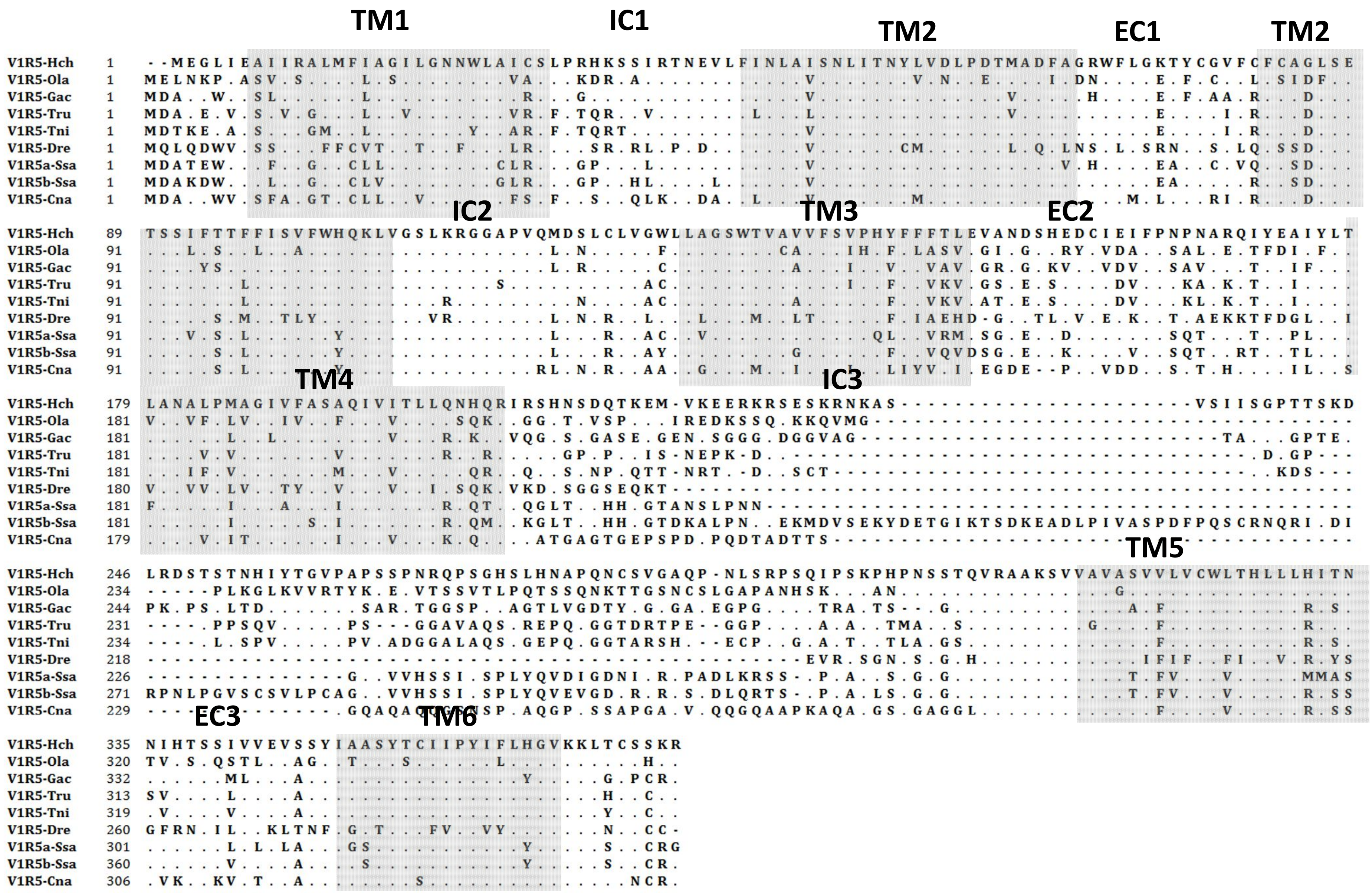
TM1
IC1
TM2
EC1
TM2
IC2
TM3
EC2
TM4
IC3
TM5
EC3
TM6
V1R5-Hch
V1R5-Ola
V1R5-Gac
V1R5-Tru
V1R5-Tni
V1R5-Dre
V1R5a-Ssa
V1R5b-Ssa
V1R5-Cna

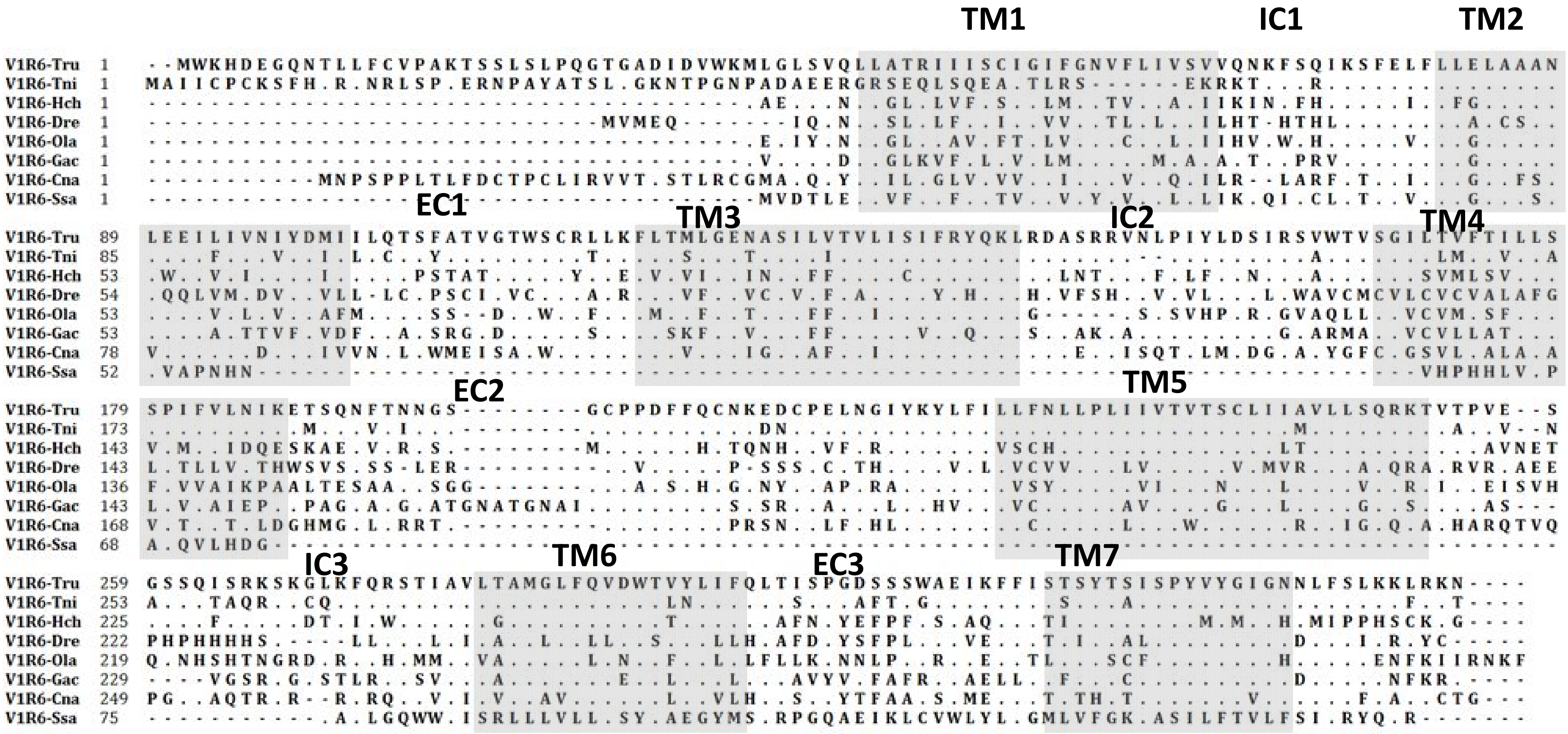
TM1
IC1
TM2
EC1
TM3
IC2
TM4
EC2
TM5
IC3
TM6
EC3
TM7
V1R6-Tru
V1R6-Tni
V1R6-Hch
V1R6-Dre
V1R6-Ola
V1R6-Gac
V1R6-Cna
V1R6-Ssa
