# Supplementary material for: Identification of a uniquely expanded V1R (ORA) gene family in the Japanese grenadier anchovy (Coilia nasus)
Source: Mar Biol. 2016 May 2;163:126. doi: 10.1007/s00227-016-2896-9 (PMC4853444; doi:10.1007/s00227-016-2896-9)
Supplement: Supplementary file 8 — Supplementary Text S8. Nucleotide sequences of V1R3 genes in Coilia nasus (PDF 227 kb) [file 227_2016_2896_MOESM8_ESM.pdf]

Electronic Supplementary Material

Identification of a uniquely expanded V1R (ORA) gene family in the Japanese grenadier anchovy (*Coilia nasus*)

Guoli Zhu[a], Wenqiao Tang[a *], Liangjiang Wang[b], Cong Wang[a], Xiaomei Wang[a]

[a] College of Fisheries and Life Science, Shanghai Ocean University, Shanghai, China

[b] Department of Genetics and Biochemistry, Clemson University, Clemson, South Carolina, United States of America

* Corresponding author: College of Fisheries and Life Science, Shanghai Ocean University, Shanghai, China; phone: + 86-21-61900425; Email: wqtang@shou.edu.cn

Supplementary Text S8. Nucleotide sequences of V1R3 genes in *Coilia nasus*.

>V1R3-3000-7

TGCTCTTCATCTCCTCCTGGGGCACCAGTATAATCTCGGTCAACTACTTCAACTAT
AATCGCGGCACATCTACGGAGTTTCTGTTGGTCATCGCTCGCTTCGCCAACATCA
CCTTCATTGCGCTGTCACCCATTGTCCTCGCAGTAGGACACGGACGCCTGCGAGC
TGTTCTGAAGTCCCTGCTCACTCACTGACAGCTGGACATGTCTGAAGGCCTGAAG
GGCTGTGGCACTGCATATAGTCAGTGTATTCGTTACCATAAATGCAGATAAGCTC
ACATCTCAAAACCACCAAAAACTGTCCTTTGGAAACCTTTGAGTGGAGATCTTGA
GCTGTTTTCAACGCTGAACACATATGCACACACGCACGCACGCACGCACACACAT
ACACACACACTGAAATTCAAAATTCATTGTGGACTCTCACTGTTCCAGAGTAAAG
TGGCCTTTTCATCACAATAAGATCTAAATTTTCTCCAGGTCTGAATATCATCAGAG
ATTTGTCTTTATTTCTGATCTCTTTCTGTTGTATTCTAAATTGCCTAAATATATCAG
ATTTCAAATGAGCCTGCGGTGTTATTATTCTGAGTCTTTATTCCCCAACTATGGTC
TTCTCTTTCACATCCATACAAACACAAAGACACACAACTGTGTTTACAGTATATT
ATCAGCAATGCTGCGCATTTCAATCCTCACATTTTTAAACTTTTTACTTTTTTTGTC
AATTTATGTTAATGTTAATCTTAATCAACTTAATGTTAGTCAACACTTCATTTGCT
TTCTACTCATGAAGCCCAATAAGCCCCTCCTCTTCCATAAAAGCAGGAACACTAC
TATACCTACAACATGTTTGACTGGGCAGTATGACAACTTTGAAGGCATTTTTCTCT
GTTTCAATGTTCGTGTAGTTACTGCACTTTGCCTCTGTAGGGCAATAATGCCTCTG
TAGGGCAATAATGTCCGGTGGTTTTGTTCAGTTAATAAGAAGAGACCTGAGTACA
CCTCTGATATGCCTGTGTCCGACCGCTAGCACAAAAGGGGAGAAGGCGATGAAC
GCGGTGTTGGCGAAGCGAACAATGACCAGCATATACTCCGTGGATGGACCACGG
TTGTAGTTAAAGTAGTTGATAGAGATCATACTGGTGCCCCAGGAGGAGATGAAG
AGCA

>V1R3-3000-12

TGCTCTTCATCTCCTCCTGGGGCACCAGTATAATCTCGGTCAACTACTTCAACTAT
AATCGCGGCACATCTACGGAGTTTCTGTTGGTCATCGCTCGCTTCGCCAACATCA
CCTTCATTGCGCTGTCACCCATTGTCCTCGCAGTAGGACACGGACGCCTGCGAGC
TGTTCTGAAGTCCCTGCTCACTCACTGACAGCTGGACATGTCTGAAGGCCTGAAG
GGCTGTGGCACTGCATATAGTCAGTGTATTCGTTACCATAAATGCAGATAAGCTC
ACATCTCAAAACCACCAAAAACTGTCCTTTGGAAACCTTTGAGTGGAGATCTTGA
GCTGTTTTCAACGCTGAACACATATGCACACACGCACGCACGCACGCACACACAT
ACACACACACTGAAATTCAAAATTCATTGTGGACTCTCACTGTTCCAGAGTAAAG
TGGCCTTTTCATCACAATAAAATCTAAATTTTCTCCAGGTCTGAATATCATCAGAG
ATTTGTCTTTATTTCTGATCTCTTTCTGTTGTATTCTAAATTGCCTAAATATATCGG
ATTTCAAATGAGCCTGCGGTGTTATTATTCTGAGTCTTTATTTCCCAACTATGGTC
TTCTCTTTCACATCCATACAAACACAAAGACACACAACTGTGTTTACAGTATATT
ATCAGCAATGCTGCGCATTTCAATCCTCACATTTTTAAACTTTTTACTTTTTTTGTC
AATTTATGTTAATGTTAATCTTAATCAACTTAATGTTAGTCAACACTTCATTTGCT
TTCTACTCATGAAGCCCAATAAGCCCCTCCTCTTCAGCTCTGCGTGATGGTACAG
GGGGGTGGGGCGGCGACAAACATGAAGCATAAGGAGAGGGAAAGCTTAAAATG
TCTGATTTTAGTGTATCACGGGTGTCAGCGCAGAGGCAGAGGCAGAGGCATCATG
ACCTGCCTGCCACGTACAGGCTTCCACAGGTCAAACCAGCCACCAAATCACATTT
GCAGTCACCATAACATGTGTGCATGATTTTGTGCTCAGGTTTTCTGTAGCTGGAA
ATCTTTGCTGTTTTTTTTTGTTTTACAATTTTTTTTTATATGGGTAATGTTGATGAT
GATCTATTCTACATGTAAGACAGCAGTGATTGGGTAGTCCCTTCCCTGACTCTTGT
AAACGTCTTGATGGTTTTAATGGCTGATGGTAACTGCTAGTGCTGCATATTTTGTC
GCGACCAGCCTTTAATTGTTTGCTCCCCTGCTATATGGGGATGTCTGACACCACAT

TTAGACATACCCCTAGGGACACAGGATGCGGCATGACAGCAATAAATATAGCGG
TGCAATAGATGGGTGAATTTGGGGAAAGCAAGCAATACACCATTTGAATAACAC
TTTGGCCTGAATGGACAATGAGAGCATAAAGCACCAAAATGAAGATTTTTGATG
GTTTACATGATAATGTAATTGGGCCAATGCATGAAGACTCTTAAATGAATAGAAT
AGAATAGAAATGAATTAAATGAAGTAAGTTGGAGCAATACTTTTTCATGTTTATA
TTTAATTCATGTAAAACACACATTCACAAACACGAACCACATAAGTGTACAAATG
ATGTAAAATAAATGAAGTAAGAAGGACGCTTAAACCAGAATTTGTTTGTCATTAG
TGGCCCCCAGTGTGCTTTCAGTCACTCTGCTTCTAGGACTTCTAGGAAAAGCGCTC
CAGTGGAAAGAGGATGTCCAGGATGTAACCATGATCATTGCAAATAACCTTGTGT
GTGTGTGTGACCTTTTCAGAAGATGGAAAACAACATAAATATATGACGTTACAAT
CTGCACTTTAATTAAATCTTGAAAAGTTGAAAATGTATGAAATGTTTAGCCAAGT
GGTTAATAAATTAAGCAAATAAGCCATGTACGAAACAGTCCCATGATATTTTCCA
GCTTTTTTAATGAAACACTTTTGTCTTCAGTGAATAAACTGTGCTATTAAAAATAC
GATGTATTCAGTAAGGTAATATGCCCTACAAAACCAAAAGGCAGTAACTACATAT
TTTGTCAAAACTGAGTTTAGACTGGAAATTGACTTTATAACACCTCCTTTGTTTTG
TGACTAAATATCTTGCTTCTTCCTAATAAGAAAGTACATAAAAGCAGGAACACTA
CTATACCTACAACATGTTTGACTGGGCAGTATGACAACTTTGAAGGCATTTTTCTC
TGTTTCAATGTTCGTGTAGTTACTGCACTTTGCCTCTGTAGGGCAATGATGCCTCT
GTAGGGCAATAATGTCCGGTGGTTTTGTTCAGTTAATAAGAAGAGACCTGAGTAC
ACCTCTGATATGCCTGTGTCCGACCGCTAGCACAAAAGGGGAGAAGGCGATGAA
CGCGGTGTTGGCGAAGCGAGCAATGACCAGCATATACTCCGTGGATGGACCACG
GTTGTAGTTAAAGTAGTTGATAGAGATCATACTGGTGCCCCAGGAGGAGATGAA
GAGCA

>V1R3-4000-33

TGCTCTTCATCTCCTCCTGGGGCACCAGTATAATCTCGGTCAACTACTTCAACTAT
AATCGCGGCACATCTACGGAGTTTCTGTTGGTCATCGCTCGCTTCGCCAACATCA
CCTTCATTGCGCTGTCACCCGTTGTCCTCGCAGTAGGACACGGACGCCTGCGAGC
TGTTCTGAAGTCCCTGCTCACTCACTGACAGCTGGACATGTCTGAAGGCCTGAAG
GGCTGTGGCACTGCATATAGTCAGTGTATTCGTTACCATAAATGCAGATAAGCTC
ACATCTCAAAACCACCAAAAACTGTCCTTTGGAAACCTTTGAGTGGAGATCTTGA
GCTGTTTTCAACGCTGAACACATATGCACACACGCACGCACGCACGCACACACAT
ACACACACACTGAAATTCAAAATTCATTGTGGACTCTCACTGTTCCAGAGTAAAG
TGGCCTTTTCATCACAATAAAATCTAAATTTTCTCCAGGTCTGAATATCATCAGAG
ATTTGTCTTTATTTCTGATCTCTTTCTGTTGTATTCTAAATTGCCTAAATATATCAG
ATTTCAAATGAGCCTGCGGTGTTATCATTCTGAGTCTTTATTTCCCAACTATGGTC
TTCTCTTTCACATCCATACAAACACAAAGACACACAACTGTGTTTACAGTATATT
ATCAGCAATGCTGCGCATTTCAATCCTCACATTTTTAAACTTTTTACTTTTTTTGTC
AATTTATGTTAATGTTAATCTTAATCAACTTAATGTTAGTCAACACTTCATTTGCT
TTCTACTCATGAAGCCCAATAAGCCCCTCCTCTTCAGCTCTGCGTGATGGTACAG
GGGGGGTGGGGCGGCGACAAACATGAAGCATAAGAGAGGGAAAGCTTAAAATG
TCTGATTTTAGTGTATCACGGGTGTCAGCGCAGAGGCAGAGGCAGAGGCATCATG
ACCTGCCTGCCACGTACAGGCTTCCACAGGTCAAACCAGCCACCAAATCACATTT
GCAGTCACCATAACATGTGTGCATGATTTTGTGCTCAGGTTTTCTGTAGCTGGAA
ATCTTTGCTGTTTTTTTTTGTTTTACAATTTTTTTTTATATGGGTTCGCTGCTGACA
CTCTACGCCCACAGCCGCTCCCTGCCGCACTCTCAGAAGAACCTCGAGATGCCCG
TCATCAGGAGGGTGCCAGCTGAGAGACGCGCTGCCAAGGTGAGTCACACTACCA
GTGCTGCTAAGGTGAGTCACACTACCAGTGCTGCTAAGGTGAGTCACACTACCAG
TGCTGCCAAAGTGAGTCACACTACCAGTGCTGCTAAGGTGAGTCACACTACCAGT
GCTGCTAAGGTGAGTCACACTACCAGTGCTGCTAAGGTGAGTCACTACCAGTGCT
GATGGTGAGTCACTATCAGTGCTGCTAGGTGATATTTAGGGTATCATTTCTTCTAC
TGTTCTCAACCCATTTTACATTACTGTACTTATTTTAAGACTGTGGCGTGCACCAC
AGTGTAATGTATATTTGAATAAACATGCTTATTGCGTGTGTACATGGTATCATTAA
AACAAACCGGTATGCACATTTCTACCTAAAATCACGGCCGTTGACACGTCAATGG

TAAAATGCATACAGTACCACAAATAATGTGGAAATTGTATCTCTCTCTCTGTCTCT
CCCTGTCTTTCTGTCTCTGTCTCTGTCTGTCTGTCTCTGTCTCTCTCTCTCTCTCTCT
CTCTCTCTCTCTCTCTCTCTGTCTCTGTCTCTCAGGTGATTCTGGCTCTAATCATGC
TCTTCATCTCACTCGACA

>V1R3-4000-26

TGCTCTTCATCTCCTCCTGGGGCACCAGTATGATCTCTATCAACTACTTTAACTAC
AACCGTGGTCCATCCACGGAGTATATGTTGGTCATTGCTCGCTTCGCCAACACCG
CGTTCATCGCCTTCTCCCCTTTTGTGCTAGCGGTCGGACACAGGCATATCAGAGGT
GTACTCAGGTCTCTTCTTATTAACTGAACAAAACCACCGGACATTATTGCCCTAC
AGAGGCATTATTGCCCTACAGAGGCAAAGTACAGTAACTACACGAACATTGAAA
CAGAGAAAAATGCCTTCAAAGTTGTCATACTGCCCAGTCAAACATGTTGTAGGTA
TAGTAGTGTTCCTGCTTTTATGTACTTTCTTATTAGGAAGAAGCAAGATATTTAGT
CACAAAACAAAGGAGGTGTTATAAAGTCAATTTCCAGTCTAAACTCAGTTTTGAC
AAAATGTGTAGTTACTGCCTTTTGGCTTTGTAGGGCATATTACCTTACTGAATACA
TCGTATTTTTAATAGCACAGTTTATTCACTGAAGACAAAAGTGTTTCATTAAAAA
AGCTGGAAAATATCATGGGACTGTTTCGTACATGGCTTATTTGCTTAATTTATTAA
CCACTTGGCTAAACATTTCATACATTTTCAACTTTTCAAGATTTAATTAAAGTGCA
GATTGTAACGTCATATATTTATGTTGTTTTCCATCTTCTGAAAAGGTCACACACAC
ACACACACACACACACACACACACACACACACACACACACACACACACACACAC
ACACACACACACACACACACACACACACACACGCACACACACTCCCCATCTCCTTCT
GACTTTGGCCACATGGGAGTTTTAATTAGGAAAACTAAATGGCAACGTTGCACAT
TTTGCCCTCACCCAGCGTATGATGATTAATTAATCCCCTGGTGTGTCCCTCTAATA
TGCACGCCCCATGGCCAAACACCCTGAAGAAAATTCCTGCCCTTTCAAGTAGCTG
AAGCTGTGTGGTGATTAAATCATCCATGCCTTGGAGTGGTTAAGCCAGCCACCCA
GAAGAAAATATCATGGACCTATGTATTGGATTGAATGAATATGAATATGATATGA
ATTGAATATGAATATGATTTGAATATTGGATGGTGATATGAATAGGAAATTGGAA
ATT

>V1R3-a (3000-7)

TGCTCTTCATCTCCTCCTGGGGCACCAGTATAATCTCGGTCAACTACTTCAACTAT
AATCGCGGCACATCTACGGAGTTTCTGTTGGTCATCGCTCGCTTCGCCAACATCA
CCTTCATTGCGCTGTCACCCATTGTCCTCGCAGTAGGACACGGACGCCTGCGAGC
TGTTCTGAAGTCCCTGCTCACTCAC

>V1R3-b (3000-7)

TGCTCTTCATCTCCTCCTGGGGCACCAGTATGATCTCTATCAACTACTTTAACTAC
AACCGTGGTCCATCCACGGAGTATATGCTGGTCATTGTTCGCTTCGCCAACACCG
CGTTCATCGCCTTCTCCCCTTTTGTGCTAGCGGTCGGACACAGGCATATCAGAGGT
GTACTCAGGTCTCTTCTTATTAAC

>V1R3-c (3000-12)

TGCTCTTCATCTCCTCCTGGGGCACCAGTATAATCTCGGTCAACTACTTCAACTAT
AATCGCGGCACATCTACGGAGTTTCTGTTGGTCATCGCTCGCTTCGCCAACATCA

CCTTCATTGCGCTGTCACCCATTGTCCTCGCAGTAGGACACGGACGCCTGCGAGC
TGTTCTGAAGTCCCTGCTCACTCAC

>V1R3-d (3000-12)

TGCTCTTCATCTCCTCCTGGGGCACCAGTATGATCTCTATCAACTACTTTAACTAC
AACCGTGGTCCATCCACGGAGTATATGCTGGTCATTGCTCGCTTCGCCAACACCG
CGTTCATCGCCTTCTCCCCTTTTGTGCTAGCGGTCGGACACAGGCATATCAGAGGT
GTACTCAGGTCTCTTCTTATTAAC

>V1R3-e (4000-33)

TGCTCTTCATCTCCTCCTGGGGCACCAGTATAATCTCGGTCAACTACTTCAACTAT
AATCGCGGCACATCTACGGAGTTTCTGTTGGTCATCGCTCGCTTCGCCAACATCA
CCTTCATTGCGCTGTCACCCGTTGTCCTCGCAGTAGGACACGGACGCCTGCGAGC
TGTTCTGAAGTCCCTGCTCACTCAC

>V1R3-g (4000-26)

TGCTCTTCATCTCCTCCTGGGGCACCAGTATGATCTCTATCAACTACTTTAACTAC
AACCGTGGTCCATCCACGGAGTATATGTTGGTCATTGCTCGCTTCGCCAACACCG
CGTTCATCGCCTTCTCCCCTTTTGTGCTAGCGGTCGGACACAGGCATATCAGAGGT
GTACTCAGGTCTCTTCTTATTAAC
