# Supplementary material for: Identification of a uniquely expanded V1R (ORA) gene family in the Japanese grenadier anchovy (Coilia nasus)
Source: Mar Biol. 2016 May 2;163:126. doi: 10.1007/s00227-016-2896-9 (PMC4853444; doi:10.1007/s00227-016-2896-9)

Electronic Supplementary Material

Identification of a uniquely expanded V1R (ORA) gene family in the Japanese grenadier anchovy (*Coilia nasus*)

Guoli Zhu[a], Wenqiao Tang[a *], Liangjiang Wang[b], Cong Wang[a], Xiaomei Wang[a]

[a] College of Fisheries and Life Science, Shanghai Ocean University, Shanghai, China

[b] Department of Genetics and Biochemistry, Clemson University, Clemson, South Carolina, United States of America

* Corresponding author: College of Fisheries and Life Science, Shanghai Ocean University, Shanghai, China; phone: + 86-21-61900425; Email: wqtang@shou.edu.cn

Supplementary Fig. S9. The alignment of V1R3-3000-7 (V1R3-3-a/V1R3-3-b) and V1R3-3000-12 (V1R3-3-c/V1R3-3-d).

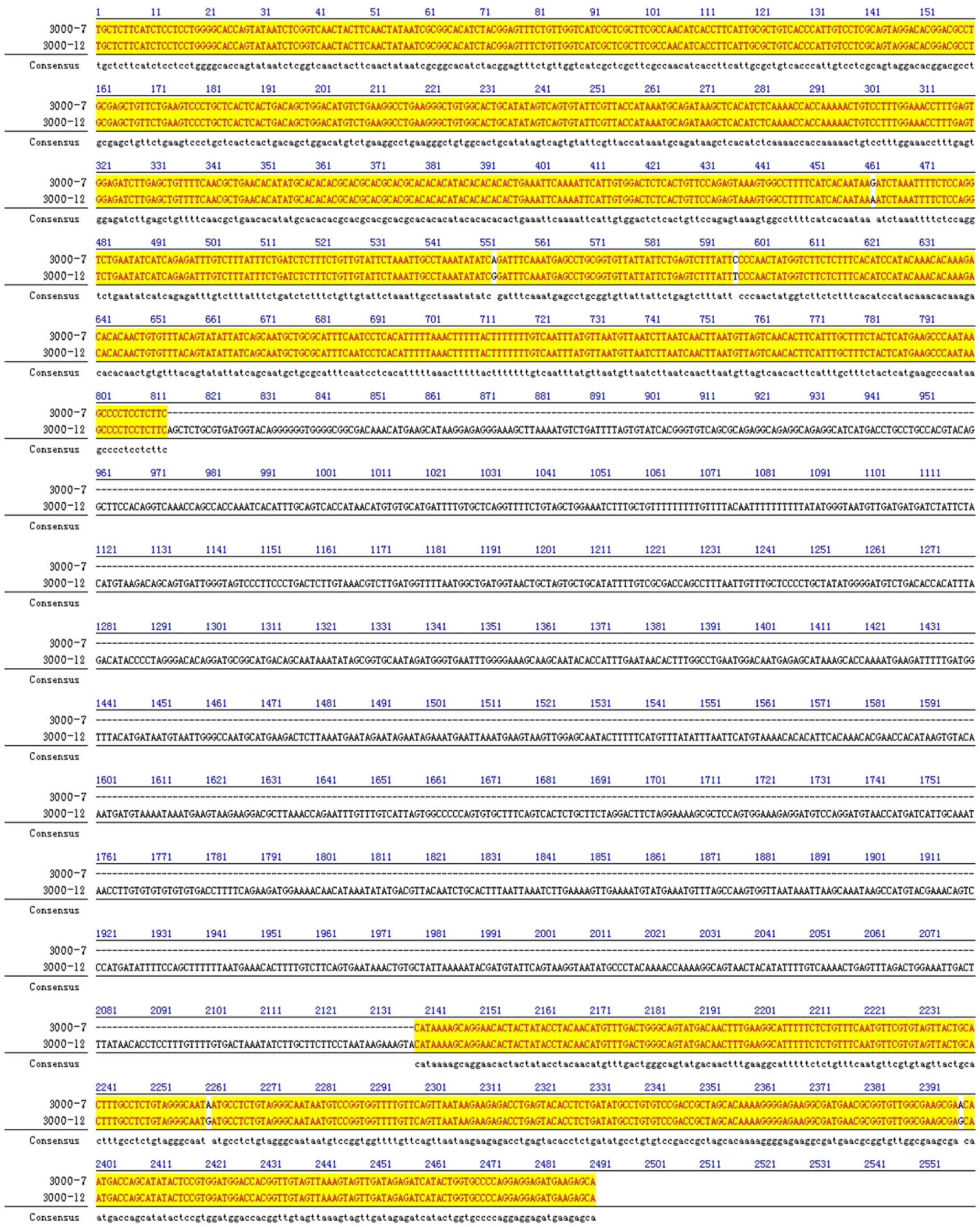

Supplement: Supplementary file 9 — Supplementary Fig. S9. The alignment of V1R3-3000-7 (V1R3-3-a/V1R3-3-b) and V1R3-3000-12 (V1R3-3-c/V1R3-3-d) (PDF 1309 kb) [file 227_2016_2896_MOESM9_ESM.pdf]
