# Supplementary material for: Identification of a uniquely expanded V1R (ORA) gene family in the Japanese grenadier anchovy (Coilia nasus)
Source: Mar Biol. 2016 May 2;163:126. doi: 10.1007/s00227-016-2896-9 (PMC4853444; doi:10.1007/s00227-016-2896-9)

Electronic Supplementary Material

Identification of a uniquely expanded V1R (ORA) gene family in the Japanese grenadier anchovy (*Coilia nasus*)

Guoli Zhu[a], Wenqiao Tang[a*], Liangjiang Wang[b], Cong Wang[a], Xiaomei Wang[a]

[a] College of Fisheries and Life Science, Shanghai Ocean University, Shanghai, China

[b] Department of Genetics and Biochemistry, Clemson University, Clemson, South Carolina, United States of America

* Corresponding author: College of Fisheries and Life Science, Shanghai Ocean University, Shanghai, China; phone: + 86-21-61900425; Email: wqtang@shou.edu.cn

Supplementary Fig. S10. Polymorphism in V1R genes from different populations of *Coilia nasus* collected from the Jingjiang section of the Yangtze River, the waters of Zhoushan, the Taihu Lake, the Poyanghu Lake and the Dongtinghu Lake. The M represents the DNA marker. Lanes 1-10 are from the Jingjiang population, 11-20 are from the Zhoushan population, 21-30 are from the Taihu Lake population, 31-40 are from the Poyanghu Lake population, and 41-50 are from the Dongtinghu lake population. Lane 0 is the negative control with sterile water used as the template.

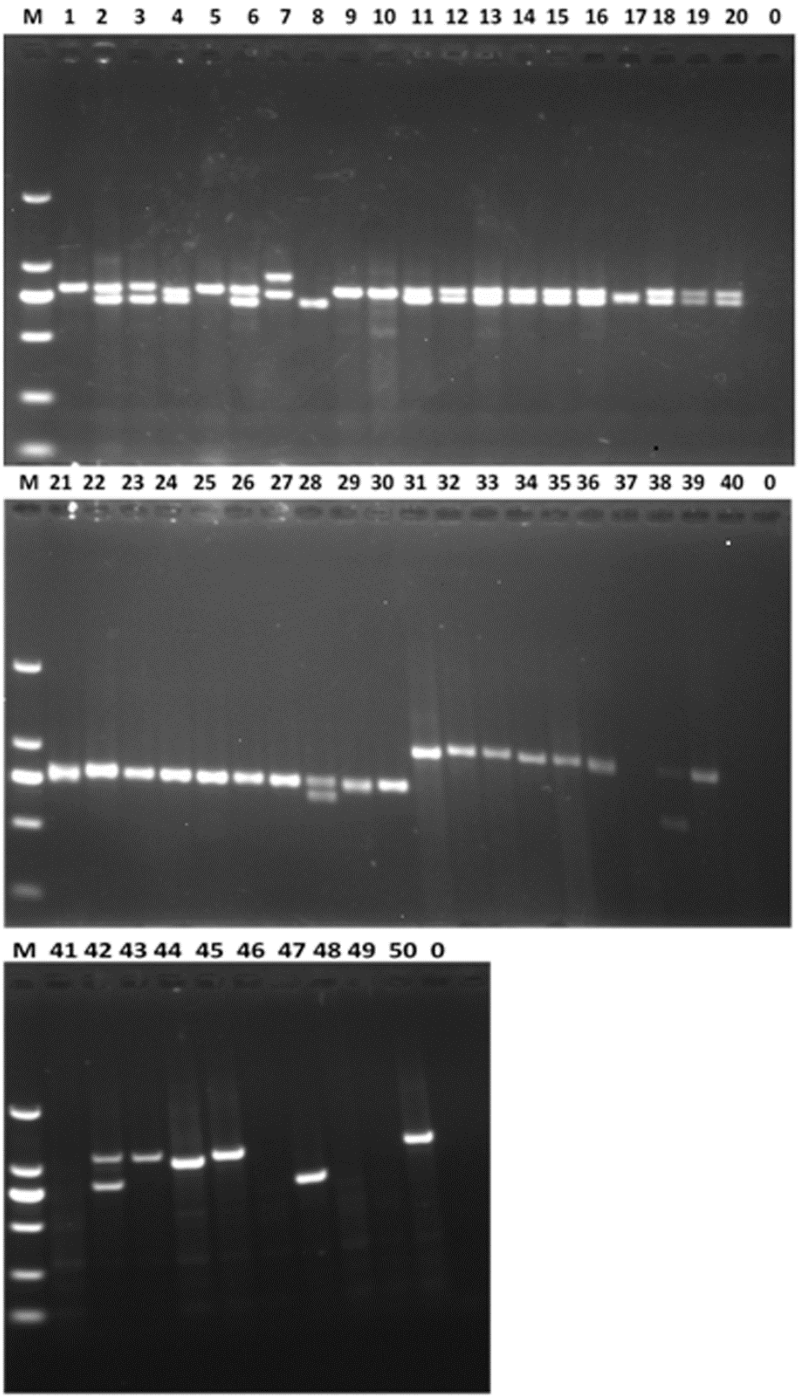

Supplement: Supplementary file 10 — Supplementary Fig. S10. Polymorphism in V1R genes from different populations of Coilia nasus collected from the Jingjiang section of the Yangtze River, the waters of Zhoushan, the Taihu Lake, the Poyanghu Lake, and the Dongtinghu Lake. The M represents the DNA marker. Lanes 1–10 are from the Jingjiang population, 11–20 are from the Zhoushan population, 21–30 are from the Taihu Lake population, 31–40 are from the Poyanghu Lake population, and 41–50 are from the Dongtinghu lake population. Lane 0 is the negative control with sterile water used as the template (PDF 479 kb) [file 227_2016_2896_MOESM10_ESM.pdf]
