# Supplementary material for: Identification of a uniquely expanded V1R (ORA) gene family in the Japanese grenadier anchovy (Coilia nasus)
Source: Mar Biol. 2016 May 2;163:126. doi: 10.1007/s00227-016-2896-9 (PMC4853444; doi:10.1007/s00227-016-2896-9)
Supplement: Supplementary file 11 — Supplementary Text S11. Nucleotide sequences of the internal region of the V1R3 gene in the populations from Jingjiang, Zhoushan, Taihu Lake, Poyanghu Lake, and Dongtinghu Lake (PDF 302 kb) [file 227_2016_2896_MOESM11_ESM.pdf]

Electronic Supplementary Material

Identification of a uniquely expanded V1R (ORA) gene family in the Japanese grenadier anchovy (*Coilia nasus*)

Guoli Zhu[a], Wenqiao Tang[a *], Liangjiang Wang[b], Cong Wang[a], Xiaomei Wang[a]

[a] College of Fisheries and Life Science, Shanghai Ocean University, Shanghai, China

[b] Department of Genetics and Biochemistry, Clemson University, Clemson, South Carolina, United States of America

* Corresponding author: College of Fisheries and Life Science, Shanghai Ocean University, Shanghai, China; phone: + 86-21-61900425; Email: wqtang@shou.edu.cn

Supplementary Text S11. Nucleotide sequences of the internal region of the V1R3 gene in the populations from Jingjiang, Zhoushan, Taihu Lake, Poyanghu Lake and Dongtinghu Lake.

```
                      10        20        30        40        50        60        70        80        90       100       110
              ....|....|....|....|....|....|....|....|....|....|....|....|....|....|....|....|....|....|....|....|....|....|
JJ19-26    1  TGACACTCTACGCCCACAGCCGCTCC-TGCTGCACTCTCAGAAGAACCTCGAGGTGCCCGTCATCAGGAGGGTGCCAGCTGAGAGACGTGCTGCCAAGGTGAGTCACACT  JJ19-26
JJ19-24    1  ..........................C.............................................................C.....................  JJ19-24
JJ19-18    1  -.........................-.............................................................C.....................  JJ19-18
ZS29-28    1  ..........................C.............................................................C.....................  ZS29-28
ZS29-15    1  ..........................C.............................................................C.....................  ZS29-15
TH2-6      1  ..........................C.............................................................C.....................  TH2-6
TH3-8      1  ..........................C.............................................................C.....................  TH3-8
PY1-4      1  ..........................C.............................................................C.....................  PY1-4
PY1-9      1  ..........................C.............................................................C.....................  PY1-9
PY1-11     1  ..........................C.............................................................C.....................  PY1-11
PY2-1      1  ..........................C.............................................................C.....................  PY2-1
PY2-11     1  ..........................C.............................................................C.....................  PY2-11
PY3-1      1  ..........................C.............................................................C.....................  PY3-1
PY3-2      1  ..........................C.............................................................C.....................  PY3-2
PY3-12     1  .......-..................C.............................................................C.....................  PY3-12
PY4-1      1  .........-................C.............................................................C.....................  PY4-1
PY4-2      1  ..........................C.............................................................C.....................  PY4-2
PY4-6      1  ..........................C.............................................................C.....................  PY4-6
DT2S-6     1  ..........................C.............................................................C.....................  DT2S-6
DT2S-7     1  ..........................C.............................................................C.....................  DT2S-7
DT2S-8     1  ..........................C.............................................................C.....................  DT2S-8
DT2S-11    1  ..........................C.............................................................C.....................  DT2S-11
DT2S-12    1  ..........................C.............................................................C.....................  DT2S-12
DT2L-3     1  ..........................C.............................................................C.....................  DT2L-3
DT2L-16    1  ..........................C.............................................................C.....................  DT2L-16
DT3-9      1  ..........................C.............................................................C.....................  DT3-9
DT3-10     1  ..........................C.............................................................C.....................  DT3-10
DT4-3      1  ..........................C.............................................................C.....................  DT4-3
DT4-2      1  ..........................C.............................................................C.....................  DT4-2
```

```
                         120       130       140       150       160       170       180       190       200       210       220
                 ....|....|....|....|....|....|....|....|....|....|....|....|....|....|....|....|....|....|....|....|....|....|
JJ19-26   110    ACCAGTGCTGCTAAGGTG-------------------------AGTCACACTACCAGTGCTGCTAAGGTGAGTC---------------------------ACACTACCA   JJ19-26
JJ19-24   111    ..................-------------------------..--------------------------------------------------------.........   JJ19-24
JJ19-18   109    ..................-------------------------...............................ACACTACCAGTGCTGCCAAAGTGAGTC.........   JJ19-18
ZS29-28   111    ..................-------------------------......--...........A.--........---------------------------.........   ZS29-28
ZS29-15   111    ..................-------------------------...............................ACACTACCAGTGCTGCCAAAGTGAGTC.........   ZS29-15
TH2-6     111    ..................-------------------------...............................---------------------------.........   TH2-6
TH3-8     111    ..................-------------------------...............................ACACTACCAGTGCTGCCAAAGTGAGTC.........   TH3-8
PY1-4     111    ..................CGTCACTACCAGTGCTGCCAAAGTG....................C..........ACACTACCAGTGCTGCTAAGGTGAGTC.........   PY1-4
PY1-9     111    .............G....CGTCACTACCAGTGCTGCCAAAGTG....................C..........ACACTACCAGTGCTGCTAAGGTGAGTC.........   PY1-9
PY1-11    111    ..................CGTCACTACCAGTGCTGCCAAAGTG....................C..........ACACTACCAGTGCTGCTAAGGTGAGTC.........   PY1-11
PY2-1     111    ..................CGTCACTACCAGTGCTGCCAAAGTG....................C..........ACACTACCAGTGCTGCTAAGGTGAGTC.........   PY2-1
PY2-11    111    ..................CGTCACTACCAGTGCTGCCAAAGTG....................C..........ACACTACCAGTGCTGCTAAGGTGAGTC.........   PY2-11
PY3-1     111    ..................CGTCACTACCAGTGCTGCCAAAGTG....................C..........ACACTACCAGTGCTGCTAAGGTGAGTC.........   PY3-1
PY3-2     111    ..................CGTCACTACCAGTGCTGCCAAAGTG....................C..........ACACTACCAGTGCTGCTAAGGTGAGTC.........   PY3-2
PY3-12    110    ..................CGTCACTACCAGTGCTGCCAAAGTG....................C..........ACACTACCAGTGCTGCTAAGGTGAGTC.........   PY3-12
PY4-1     110    ..................CGTCACTACCAGTGCTGCCAAAGTG....................C..........ACACTACCAGTGCTGCTAAGGTGAGTC.........   PY4-1
PY4-2     111    ..................CGTCACTACCAGTGCTGCCAAAGTG....................C..........ACACTACCAGTGCTGCTAAGGTGAGTC.........   PY4-2
PY4-6     111    ..................CGTCACTACCAGTGCTGCCAAAGTG....................C..........ACACTACCAGTGCTGCTAAGGTGAGTC.........   PY4-6
DT2S-6    111    ..................-------------------------...............................---------------------------.........   DT2S-6
DT2S-7    111    ..................-------------------------...............................---------------------------.........   DT2S-7
DT2S-8    111    ..................-------------------------...............................---------------------------.........   DT2S-8
DT2S-11   111    ..................-------------------------...............................---------------------------.........   DT2S-11
DT2S-12   111    ..................-------------------------...............................---------------------------.........   DT2S-12
DT2L-3    111    ..................CGTCACTACCAGTGCTGCCAAAGTG....................C..........ACACTACCAGTGCTGCTAAGGTGAGTC.........   DT2L-3
DT2L-16   111    ..................CGTCACTACCAGTGCTGCCAAAGTG....................C..........ACACTACCAGTGCTGCTAAGGTGAGTC.........   DT2L-16
DT3-9     111    ..................CGTCACTACCAGTGCTGCCAAAGTG....................C..........ACACTACCAGTGCTGCTAAGGTGAGTC.........   DT3-9
DT3-10    111    ..................CGTCACTACCAGTGCTGCCAAAGTG....................C..........ACACTACCAGTGCTGCTAAGGTGAGTC.........   DT3-10
DT4-3     111    ..................CGTCACTACCAGTGCTGCCAAAGTG....................C..........ACACTACCAGTGCTGCTAAGGTGAGTC.........   DT4-3
```

```
DT4-2    111 ..................CGTCACTACCAGTGCTGCCAAAGTG....................C..........ACACTACCAGTGCTGCTAAGGTGAGTC......... DT4-2

                    230       240       250       260       270       280       290       300       310       320       330
             ....|....|....|....|....|....|....|....|....|....|....|....|....|....|....|....|....|....|....|....|....|....|
JJ19-26  168 GTGCTGCCAAAGTGAGTCACACTACCAGTGCTGCTAAGGTGAGTCACACTACCAGTGCTGCTAAGGTGAGTCACACTACCAGTGCTGCTAAGGTGAGTCAC--------- JJ19-26
JJ19-24  140 .......T..G..................................................C..A....................................--------- JJ19-24
JJ19-18  194 .....................................................................................................--------- JJ19-18
ZS29-28  165 ..........G.......................C.....................................................C............A-------- ZS29-28
ZS29-15  196 .....................................................................................................--------- ZS29-15
TH2-6    169 .....................................................................................................--------- TH2-6
TH3-8    196 .......T..G..........................................................................................--------- TH3-8
PY1-4    221 .......T..G.C.................................................................................A......TACCAGTGC PY1-4
PY1-9    221 .......T..G...................................................................................A......TACCAGTGC PY1-9
PY1-11   221 .......T..G...................................................................................A......TACCAGTGC PY1-11
PY2-1    221 .......T..G...................................................................................A......TACCAGTGC PY2-1
PY2-11   221 .......T..G.......................................................C...........................A......TACCAGTGC PY2-11
PY3-1    221 .......T..G...................................................................................A......TACCAGTGC PY3-1
PY3-2    221 .......T..G...................................................................................A......TACCAGTGC PY3-2
PY3-12   220 .......T..G...................................................................................A......TACCAGTGC PY3-12
PY4-1    220 .......T..G...................................................................................A......TACCAGTGC PY4-1
PY4-2    221 .......T..G..........................A........................................................A......TACCAGTGC PY4-2
PY4-6    221 .......T..G...................................................................................A......TACCAGTGC PY4-6
DT2S-6   169 .....................................................................................................--------- DT2S-6
DT2S-7   169 .....................................................................................................--------- DT2S-7
DT2S-8   169 .....................................................................................................--------- DT2S-8
DT2S-11  169 .....................................................................................................--------- DT2S-11
DT2S-12  169 .....................................................................................................--------- DT2S-12
DT2L-3   221 .......T..G...................................................................................A......TACCAGTGC DT2L-3
DT2L-16  221 .......T..G...................................................................................A......TACCAGTGC DT2L-16
DT3-9    221 .......T..G...................................................................................A......TACCAGTGC DT3-9
DT3-10   221 .......T..G...................................................................................A......TACCAGTGC DT3-10
```

```
DT4-3    221 .......T..G...................................................................................A......TACCAGTGC DT4-3
DT4-2    221 .......T..G...................................................................................A......TACCAGTGC DT4-2

                     340       350       360       370       380       390       400       410       420       430       440
             ....|....|....|....|....|....|....|....|....|....|....|....|....|....|....|....|....|....|....|....|....|....|
JJ19-26  268 ----------------------------------------------------------------------TATCAGTGCTGCTAAGGT---------------------- JJ19-26
JJ19-24  240 ----------------------------------------------------------------------..C...............---------------------- JJ19-24
JJ19-18  294 ----------------------------------------------------------------------..................---------------------- JJ19-18
ZS29-28  266 ---------------------------------------------------------------------C..C...............---------------------- ZS29-28
ZS29-15  296 ----------------------------------------------------------------------..................---------------------- ZS29-15
TH2-6    269 ----------------------------------------------------------------------..................---------------------- TH2-6
TH3-8    296 ----------------------------------------------------------------------..................---------------------- TH3-8
PY1-4    331 TGCTAAGGTGCGTCACTACCAGTGCTGCCAAAGTGAGTCACACTACCAGTGCTGCCAAGGTGAGTCACAC..C...............GAGTCACACTACCAGTGCTGCT PY1-4
PY1-9    331 TGCTAAGGTGCGTCACTACCAGTGCTGCCAAAGTGAGCCACACTACCAGTGCTGCCAAGGTGAGTCACAC..C...............GAGTCACACTACCAGTGCTGCT PY1-9
PY1-11   331 TGCTAAGGTGCGTCACTACCAGTGCTGCCAAAGTGAGTCACACTACCAGTGCTGCCAAGGTGAGTCACAC..C...............GAGTCACACTACCAGTGCTGCT PY1-11
PY2-1    331 TGCTAAGGTGCGTCACTACCAGTGCTGCCAAAGTGAGTCACACTACCAGTGCTGCCAAGGTGAGTCACAC..C...............GAGTCACACTACCAGTGCTGCT PY2-1
PY2-11   331 TGCTAAGGTGCGTCACTACCAGTGCTGCCAAAGTGAGTCACACTACCAGTGCTGCCAAGGTGAGTCACAC..C...............GAGTCACACTACCAGTGCTGCT PY2-11
PY3-1    331 TGCTAAGGTG----------------------------------------------------AGTCACAC..C...............---------------------- PY3-1
PY3-2    331 TGCTAAGGTGCGTCACTACCAGTGCTGCCAAAGTGAGTCACACTACCAGTGCTGCCAAGGTGAGTCACAC..C...............GAGTCACACTACCAGTGCTGCT PY3-2
PY3-12   330 TGCTAAGGTGCGTCACTACCAGTGCTGCCAAAGTGAGTCACACTACCAGTGCTGCCAAGGTGAGTCACAC..C...............GAGTCACACTACCAGTGCTGCT PY3-12
PY4-1    330 TGCTAAGGTGCGTCACTACCAGTGCTGCCAAAGTGAGTCACACTACCAGTGCTGCCAAGGTGAGTCACAC..C...............GAGTCACACTACCAGTGCTGCT PY4-1
PY4-2    331 TGCTAAGGTGCGTCACTACCAGTGCTGCCAAAGTGAGTCACACTACCAGTGCTGCCAAGGTGAGTCACAC..C...............GAGTCACACTACCAGTGCTGCT PY4-2
PY4-6    331 TGCTAAGGTGCGTCACTACCAGTGCTGCCAAAGTGAGTCACACTACCAGTGCTGCCAAGGTGAGTCACAC..C...............GAGTCACACTACCAGTGCTGCT PY4-6
DT2S-6   269 ----------------------------------------------------------------------..................---------------------- DT2S-6
DT2S-7   269 ----------------------------------------------------------------------..................---------------------- DT2S-7
DT2S-8   269 ----------------------------------------------------------------------..................---------------------- DT2S-8
DT2S-11  269 ----------------------------------------------------------------------..................---------------------- DT2S-11
DT2S-12  269 ----------------------------------------------------------------------..................---------------------- DT2S-12
DT2L-3   331 TGCTAAGGTGCGTCACTACCAGTGCTGCCAAAGTGAGTCACACTACCAGTGCTGCCAAGGTGAGTCACAC..C...............GAGTCACACTACCAGTGCTGCT DT2L-3
DT2L-16  331 TGCTAAGGTGCGTCACTACCAGTGCTGCCAAAGTGAGTCACACTACCAGTGCTGCCAAGGTGAGTCACAC..C...............GAGTCACACTACCAGTGCTGCT DT2L-16
DT3-9    331 TGCTAAGGTGCGTCACTACCAGTGCTGCCAAAGTGAGTCACACTACCAGTGCTGCTAAGGTGAGTCACAC..C...............AAGTCA--CTACCAGTGCTGCT DT3-9
```

```
DT3-10   331 TGCTAAGGTGCGTCACTACCAGTGCTGCCAAAGTGAGTCACACTACCAGTGCTGCCAAGGTGAGTCACAC..C...............GAGTCACACTACCAGTGCTGCT DT3-10
DT4-3    331 TGCTAAGGTGCGTCACTACCAGTGCTGCCAAAGTGAGTCACACTACCAGTGCTGCCAAGGTGAGTCACAC..C...............GAGTCACACTACCAGTGCTGCT DT4-3
DT4-2    331 TGCTAAGGTGCGTCACTACCAGTGCTGCCAAAGTGAGTCACACTACCAGTGCTGCCAAGGTGAGTCACAC..C...............GAGTCACACTACCAGTGCTGCT DT4-2

                       450       460       470       480       490       500       510       520       530       540       550
             ....|....|....|....|....|....|....|....|....|....|....|....|....|....|....|....|....|....|....|....|....|....|
JJ19-26  286 -------------------------------------------------------------------------------------------------------------- JJ19-26
JJ19-24  258 -------------------------------------------------------------------------------------------------------------- JJ19-24
JJ19-18  312 -------------------------------------------------------------------------------------------------------------- JJ19-18
ZS29-28  285 -------------------------------------------------------------------------------------------------------------- ZS29-28
ZS29-15  314 -------------------------------------------------------------------------------------------------------------- ZS29-15
TH2-6    287 -------------------------------------------------------------------------------------------------------------- TH2-6
TH3-8    314 -------------------------------------------------------------------------------------------------------------- TH3-8
PY1-4    441 AAGGTGAGTCACACTACCAGTGCTGCTAAGGT---------------------------AAGTCACTACCAGTGCTGCTAAGGTGAGTCACACTACCAGTGCTGCTAAGG PY1-4
PY1-9    441 AAGGTGAGTCACACTACCAGTGCTGCTAAGGT---------------------------AAGTCACTACCAGTGCTGCTAAGGTGAGTCACACTACCAGTGCTGCTAAGG PY1-9
PY1-11   441 AAGGTGAGTCACACTACCAGTGCTGCTAAGGT---------------------------AAGTCACCACCAGTGCTGCTAAGGTGAGTCACACTACCAGTGCTGCTAAGG PY1-11
PY2-1    441 AAGGTGAGTCACACTACCAGTGCTGCTAAGGTGAGTCACACTACCAGTGCTGCTAAGGTAAGTCACTACCAGTGCTGCTAAGGTGAGTCACACTACCAGTGCTGCTAAGG PY2-1
PY2-11   441 AAGGTGAGTCACACTACCAGTGCTGCTAAGGTGAGTCACACTACCAGTGCTGCTAAGGTAAGTCACTACCAGTGCTGCTAAGGTGAGTCACACTACCAGTGCTGCTAAGG PY2-11
PY3-1    366 -------------------------------------------------------------------------------------------------------------- PY3-1
PY3-2    441 AAGGTGAGTCACACTACCAGTGCTGCTAAGGTGAGTCACACTACCAGTGCTGCTAAGGTAAGTCACTACCAGTGCTGCTAAGGTGAGTCACACTACCAGTGCTGCTAAGG PY3-2
PY3-12   440 AAGGTGAGTCACACTACCAGTGCTGCTAAGGTGAGTCACACTACCAGTGCTGCTAAGGTAAGTCACTACCAGTGCTGCTAAGGTGAGTCACACTACCAGTGCTGCTAAGG PY3-12
PY4-1    440 AAGGTGAGTCACACTACCAGTGCTGCTAAGGTGAGTCACACTACCAGTGCTGCTAAGGTAAGTCACTACCAGTGCTGCTAAGGTGAGTCACACTACCAGTGCTGCTAAGG PY4-1
PY4-2    441 AAGGTGAGTCACACTACCAGTGCTGCTAAGGTGAGTCACACTACCAGTGCTGCTAAGGTAAGTCACTACCAGTGCTGCTAAGGTGAGTCACACTACCAGTGCTGCTAAGG PY4-2
PY4-6    441 AAGGTGAGTCACACTACCAGTGCTGCTAAGGTGAGTCACACTACCAGTGCTGCTAAGGTAAGTCACTACCAGTGCTGCTAAGGTGAGTCACACTACCAGTGCTGCTAAGG PY4-6
DT2S-6   287 -------------------------------------------------------------------------------------------------------------- DT2S-6
DT2S-7   287 -------------------------------------------------------------------------------------------------------------- DT2S-7
DT2S-8   287 -------------------------------------------------------------------------------------------------------------- DT2S-8
DT2S-11  287 -------------------------------------------------------------------------------------------------------------- DT2S-11
DT2S-12  287 -------------------------------------------------------------------------------------------------------------- DT2S-12
DT2L-3   441 AAGGTGAGTCACACTACCAGTGCTGCTAAGGTGAGTCACACTACCAGTGCTGCTAAGGTAAGTCACTACCAGTGCTGCTAAGGTGAGTCACACTACCAGTGCTGCTAAGG DT2L-3
DT2L-16  441 AAGGTGAGTCACACTACCAGTGCTGCTAAGGTGAGTCACACTACCAGTGCTGCTAAGGTAAGTCACTACCAGTGCTGCTAAGGTGAGTCACACTACCAGTGCTGCTAAGG DT2L-16
```

```
DT3-9      439  AAGGTGAGTCACACTACCAGTGCTGCTAAGGT------------------------------------------------------------------------------ DT3-9
DT3-10     441  AAGGTGAGTCACACTACCAGTGCTGCTAAGGTGAGTCACACTACCAGTGCTGCTAAGGTAAGTCACTACCAGTGCTGCTAAGGTGAGTCACACTACCAGTGCTGCTAAGG DT3-10
DT4-3      441  AAGGTGAGTCACACTACCAGTGCTGCTAAGGT------------------------------------------------------------------------------ DT4-3
DT4-2      441  AAGGTGAATCACACTACCAGTGCTGCTAAGGT------------------------------------------------------------------------------ DT4-2

                        560       570       580       590       600       610       620       630       640       650       660
                ....|....|....|....|....|....|....|....|....|....|....|....|....|....|....|....|....|....|....|....|....|....|
JJ19-26    286  -GAGTCACACTACCAGTGCTGCTAAGGTGAGTCACACTACCAGTGCTGCTAAGGTGAGTCAC-----------------------ACTACCAGTGCTGATGGTGAGTCAC JJ19-26
JJ19-24    258  -..................................----------------------------------------------------....................... JJ19-24
JJ19-18    312  -.............................................................-----------------------......................... JJ19-18
ZS29-28    285  -....................--.T........................C...........-------------------------........................ ZS29-28
ZS29-15    314  -.............................................................-----------------------......................... ZS29-15
TH2-6      287  -.............................................................-----------------------......................... TH2-6
TH3-8      314  -.............................................................-----------------------......................... TH3-8
PY1-4      524  T......................................................A......TACCAGTGCTGCTAAGGTGAGTC......................... PY1-4
PY1-9      524  T......................................................A......TACCAGTGCTGCTAAGGTGAGTC......................... PY1-9
PY1-11     524  T......................................................A......TACCAGTGCTGCTAAGGTGAGTC......................... PY1-11
PY2-1      551  T......................................................A......TACCAGTGCTGCTAAGGTGAGTC......................... PY2-1
PY2-11     551  T......................................................A......TACCAGTGCTGCTAAGGTGAGTC......................... PY2-11
PY3-1      366  -......................................................A......TACCAGTGCTGCTAAGGTGAGTC......................... PY3-1
PY3-2      551  T......................................................A......TACCAGTGCTGCTAAGGTGAGTC......................... PY3-2
PY3-12     550  T......................................................A......TACCAGTGCTGCTAAGGTGAGTC......................... PY3-12
PY4-1      550  T......................................................A......TACCAGTGCTGCTAAGGTGAGTC......................... PY4-1
PY4-2      551  T......................................................A......TACCAGTGCTGCTAAGGTGAGTC......................... PY4-2
PY4-6      551  T......................................................A......TACCAGTGCTGCTAAGGTGAGTC......................... PY4-6
DT2S-6     287  -.............................................................-----------------------......................... DT2S-6
DT2S-7     287  -.............................................................-----------------------......................... DT2S-7
DT2S-8     287  -.............................................................-----------------------......................... DT2S-8
DT2S-11    287  -.............................................................-----------------------......................... DT2S-11
DT2S-12    287  -.............................................................-----------------------......................... DT2S-12
DT2L-3     551  T......................................................A......TACCAGTGCTGCTAAGGTGAGTC......................... DT2L-3
```

```
DT2L-16    551 T......................................................A......TACCAGTGCTGCTAAGGTGAGTC......................... DT2L-16
DT3-9      470 -......................................................A......TACCAGTGCTGCTAAGGTGAGTC......................... DT3-9
DT3-10     551 T......................................................A......TACCAGTGCTGCTAAGGTGAGTC......................... DT3-10
DT4-3      472 -......................................................A......TACCAGTGCTGCTAAGGTGAGTC......................... DT4-3
DT4-2      472 -......................................................A......TACCAGTGCTGCTAAGGTGAGTC......................... DT4-2

                      670       680       690       700       710       720       730       740       750       760       770
               ....|....|....|....|....|....|....|....|....|....|....|....|....|....|....|....|....|....|....|....|....|....|
JJ19-26    373 TATCAGTGCTGCTAGGTGATATTTTAGGGTATCATTTCTTCTACTGTTCTCAACCCATTTTACATTACTGTACTTATTTTAAGACTGTGGCGTGCACCACAGTGTAATGT JJ19-26
JJ19-24    316 .............................................................................................................. JJ19-24
JJ19-18    399 .............................................................................................................. JJ19-18
ZS29-28    368 .............................................................................................................. ZS29-28
ZS29-15    401 .............................................................................................................. ZS29-15
TH2-6      374 .............................................................................................................. TH2-6
TH3-8      401 .............................................................................................................. TH3-8
PY1-4      634 .............................................................................................................. PY1-4
PY1-9      634 .............................................................................................................. PY1-9
PY1-11     634 .............................................................................................................. PY1-11
PY2-1      661 .............................................................................................................. PY2-1
PY2-11     661 ...................................................G.......................................................... PY2-11
PY3-1      476 .........................................................................................................G.... PY3-1
PY3-2      661 .............................................................................................................. PY3-2
PY3-12     660 .............................................................................................................. PY3-12
PY4-1      660 .............................................................................................................. PY4-1
PY4-2      661 .............................................................................................................. PY4-2
PY4-6      661 .............................................................................................................. PY4-6
DT2S-6     374 .............................................................................................................. DT2S-6
DT2S-7     374 .............................................................................................................. DT2S-7
DT2S-8     374 .............................................................................................................. DT2S-8
DT2S-11    374 .............................................................................................................. DT2S-11
DT2S-12    374 .............................................................................................................. DT2S-12
```

```
DT2L-3    661  .........................................C....................................................................  DT2L-3
DT2L-16   661  ..............................................................................................................  DT2L-16
DT3-9     580  ..............................................................................................................  DT3-9
DT3-10    661  ..............................................................................................................  DT3-10
DT4-3     582  ..............................................................................................................  DT4-3
DT4-2     582  ..............................................................................................................  DT4-2

                        780       790       800       810       820       830       840       850       860       870       880
               ....|....|....|....|....|....|....|....|....|....|....|....|....|....|....|....|....|....|....|....|....|....|
JJ19-26   483  ATATTTGAATAAACATGTTTATTGCGTGTGTACATGGTATCATTAAAACAAACCGGTATGCACATTTCTACCTAAAATCACGGTCGTTGACACGTCAATGGTAAAATGCA  JJ19-26
JJ19-24   426  .................C..................................................................T.........................  JJ19-24
JJ19-18   509  .................C............................................................................................  JJ19-18
ZS29-28   478  .................C..................................................................T.........................  ZS29-28
ZS29-15   511  .................C............................................................................................  ZS29-15
TH2-6     484  .................C............................................................................................  TH2-6
TH3-8     511  .................C............................................................................................  TH3-8
PY1-4     744  .................C..................................................................T.........................  PY1-4
PY1-9     744  .................C..................................................................T.........................  PY1-9
PY1-11    744  .................C..................................................................T.........................  PY1-11
PY2-1     771  .................C..................................................................T.........................  PY2-1
PY2-11    771  .................C..................................................................T.........................  PY2-11
PY3-1     586  .................C..................................................................T.........................  PY3-1
PY3-2     771  .................C..................................................................T.........................  PY3-2
PY3-12    770  .................C..................................................................T.........................  PY3-12
PY4-1     770  .................C..............................................C...................T.........................  PY4-1
PY4-2     771  .................C..................................................................T.........................  PY4-2
PY4-6     771  .................C.................................................................GT.........................  PY4-6
DT2S-6    484  .................C............................................................................................  DT2S-6
DT2S-7    484  .................C............................................................................................  DT2S-7
DT2S-8    484  .................C............................................................................................  DT2S-8
DT2S-11   484  .................C............................................................................................  DT2S-11
```

```
DT2S-12  484 .................C.....................................................................C...................... DT2S-12
DT2L-3   771 .................C..................................................................T......................... DT2L-3
DT2L-16  771 .................C..................................................................T......................... DT2L-16
DT3-9    690 .................C..................................................................T......................... DT3-9
DT3-10   771 .................C..................................................................T......................... DT3-10
DT4-3    692 .................C..................................................................T......................... DT4-3
DT4-2    692 .................C..................................................................T......................... DT4-2

                      890       900       910       920       930       940       950       960       970       980       990
             ....|....|....|....|....|....|....|....|....|....|....|....|....|....|....|....|....|....|....|....|....|....|
JJ19-26  593 TACAGTACCACAAATAATGTGGAAATTGTATCTCTCTCTCTGTCTCTCCCTGTCTTTCTGTCTCTGTCTCTGTCTGTCT--GTCTCTGTCTCTCTGTCTCTGTCTCTCTC JJ19-26
JJ19-24  536 .................G............................GT.....................------....--....................--------- JJ19-24
JJ19-18  619 ...............................................................................--....................C........ JJ19-18
ZS29-28  588 ......G........................................................................--....................--------- ZS29-28
ZS29-15  621 ...............................................................................--....................C........ ZS29-15
TH2-6    594 ...............................................................................--............................. TH2-6
TH3-8    621 ...............................................................................--............................. TH3-8
PY1-4    854 ...............................................................................--............G.C.....C........ PY1-4
PY1-9    854 ...............................................................................--............G.C.....C........ PY1-9
PY1-11   854 ...............................................................................--............G.C.....C........ PY1-11
PY2-1    881 ...............................................................................--............G.C.....C........ PY2-1
PY2-11   881 ...............................................................................--............G.C.....C........ PY2-11
PY3-1    696 ...............................................................................--............G.C.....C........ PY3-1
PY3-2    881 ...............................................................................--............G.C.....C........ PY3-2
PY3-12   880 ...............................................................................--............G.C.....C........ PY3-12
PY4-1    880 ...............................................................................--............G.C.....C........ PY4-1
PY4-2    881 ................................C..............................................--............G.C.....C........ PY4-2
PY4-6    881 ...............................................................................--............G.C.....C........ PY4-6
DT2S-6   594 ...............................................................................--............................. DT2S-6
DT2S-7   594 ...............................................................................--............................. DT2S-7
DT2S-8   594 ...............................................................................--............................. DT2S-8
```

```
DT2S-11  594  ...............................................................................--.............................  DT2S-11
DT2S-12  594  ...............................................................................--.............................  DT2S-12
DT2L-3   881  ...............................................................................--............G.C.....C........  DT2L-3
DT2L-16  881  ...............................................................................--............G.C.....C........  DT2L-16
DT3-9    800  ...............................................................................--............G.C.....C........  DT3-9
DT3-10   881  ...............................................................................--............G.C.....C........  DT3-10
DT4-3    802  ...............................................................................CT..............C.....C........  DT4-3
DT4-2    802  ...............................................................................--............G.C.....C........  DT4-2

                      1000      1010      1020      1030      1040      1050      1060      1070      1080      1090
              ....|....|....|....|....|....|....|....|....|....|....|....|....|....|....|....|....|....|....|....|...
JJ19-26  701  TCTCT----------CTCTCTCTCTCTCT------------GTCTCTGTCTCTCAGGTGATTCTGGCTCTAATCATGCTCTTCATCTCCTCCTGGGGCACCAG  JJ19-26
JJ19-24  628  ---------------..G...........------------..............................................................  JJ19-24
JJ19-18  727  .....CTCTCTCT--..............------------.C............................................................  JJ19-18
ZS29-28  686  -------------------..........------------..............................................................  ZS29-28
ZS29-15  729  .....CTCTCTCTCT..............------------..............T...............................................  ZS29-15
TH2-6    702  .....CTCTCTCT--..............------------..............................................................  TH2-6
TH3-8    729  .....CTCTCTCT--..............------------..............................................................  TH3-8
PY1-4    962  .....CTCTCTCTCT.............----------TCT..............................................................  PY1-4
PY1-9    962  .....CTCTCTCTCT..------------------------..............................................................  PY1-9
PY1-11   962  .....CTCTCTCTCT..------------------------..............................................................  PY1-11
PY2-1    989  .....CTCTCTCTCT.............----TCTCTCTCT.................................C............................  PY2-1
PY2-11   989  .....CTCTCTCTCT.............------TCTCTCT..............................................................  PY2-11
PY3-1    804  .....CTCTCTCTCT..............C----TGTCTCT..............................................................  PY3-1
PY3-2    989  .....CTCTCTCTCT.............CC--TCTCTCTCT..............................................................  PY3-2
PY3-12   988  .....CTCTCTCTCT..............CTCTCTCTCTCT..............................................................  PY3-12
PY4-1    988  .....CTCTCTCTCT........------------------..............................................................  PY4-1
PY4-2    989  .....CTCTCTCTCT..........----------------..............................................................  PY4-2
PY4-6    989  .....CTCTCTCTCT........------------------..............................................................  PY4-6
DT2S-6   702  .....CTCTCTCT--..............------------.C............................................................  DT2S-6
DT2S-7   702  .....CTCTCTCT--..............------------..............................................................  DT2S-7
```

```
DT2S-8    702  .....CTCT------..............------------..............................................................  DT2S-8
DT2S-11   702  .....CTCTCT----..............------------..............................................................  DT2S-11
DT2S-12   702  .....CTCTCTCT--..............------------..............................................................  DT2S-12
DT2L-3    989  .....CTCTCTCTCT.............----TCTCTCTCT..............................................................  DT2L-3
DT2L-16   989  .....CTCTCTCTCT.............--------TCTCT..............................................................  DT2L-16
DT3-9     908  .....CTCTCTCTCT..........----------------..............................................................  DT3-9
DT3-10    989  .....CTCTCTCTCT............--------------..............................................................  DT3-10
DT4-3     912  .....CTCTCTCTCT........------------------..............................................................  DT4-3
DT4-2     910  .....CTCTCTCTCT.............----------TCT..............................................................  DT4-2
```
